# Single-cell transcriptomics reveals cellular and genetic mechanisms of alpine adaptation in *Rosa sericea*

**DOI:** 10.3389/fpls.2026.1733247

**Published:** 2026-02-19

**Authors:** Hengning Deng, Jian Ru, Zhenlong Liang, Zhongyu Tang, Yang Wang, Wenqin Yuan, Liangying Li, Yu Feng, Xinfen Gao

**Affiliations:** 1 Mountain Ecological Restoration and Biodiversity Conservation Key Laboratory of Sichuan Province, Chengdu Institute of Biology, Chinese Academy of Sciences, Chengdu, Sichuan, China; 2 China-Croatia Belt and Road Joint Laboratory on Biodiversity and Ecosystem Services, Chengdu Institute of Biology, Chinese Academy of Sciences, Chengdu, Sichuan, China; 3 University of Chinese Academy of Sciences, Beijing, China; 4 Key Laboratory for Regional Plants Conservation and Ecological Restoration of Northeast Jiangxi, College of Life Science, Shangrao Normal University, Shangrao, Jiangxi, China

**Keywords:** gene co-expression modules, high-altitude adaptation, *Rosa sericea*, single-cell RNA sequencing, trichome

## Abstract

**Introduction:**

Plant development is shaped by environmental conditions, and its adaptation to climate change is crucial for biodiversity conservation. The extreme climate of the Qinghai-Tibet Plateau makes it an ideal system for studying plant adaptive strategies. *Rosa sericea*, a dominant alpine shrub, exhibits remarkable morphological plasticity, but its molecular and cellular adaptation mechanisms are still unclear. In this study, we integrated single-nucleus RNA sequencing (snRNA-seq) with high-dimensional weighted gene co-expression network analysis (hdWGCNA), gene ontology (GO) enrichment, gene set enrichment analysis (GSEA), pseudotime trajectory inference, and gene overexpression techniques to profile 31,796 cells from *R. sericea* leaves.

**Methods:**

We constructed a draft single-cell transcriptional atlas with putative annotation of 11 leaf cell types and identified eight co-expression gene modules linked to key cell types.

**Results:**

The leaf development spatiotemporal dynamics uncovered a developmental continuum from cell proliferation to photosynthetically specialized maturation. Furthermore, we identified several developmental and physiological features potentially associated with high-altitude adaptation, including presence of transcriptionally active nuclear-encoded genes involved in chloroplast function in epidermal pavement cells, the potential role of SPL7-mediated copper homeostasis, and a putative *RO6G37307–TTG2–TCP4* regulatory module associated with trichome development.

**Discussion:**

Together, this study provides the first single-cell–resolved transcriptional framework for *R. sericea* leaves and suggests adaptive developmental mechanisms at the cellular and genetic levels, enhancing our understanding of how alpine plants respond to climate change.

## 1 Introduction

Global climate warming presents significant challenges to plant survival (Kumar et al., 2024a; Seth and Sebastian, 2024; Sun et al., 2025), profoundly affecting their growth and development (Li et al., 2019; Saladin et al., 2020; Reich et al., 2022; Wang et al., 2022b; Sigdel et al., 2024). These effects are especially evident in evolutionary and developmental characteristics (Wright et al., 2017; Yuan et al., 2019; Cabon et al., 2022; Gao et al., 2022; Meng et al., 2024). One of the most notable consequences is the advancement of leaf unfolding in spring and delayed senescence during autumn (Fu et al., 2015; Dow et al., 2022; Marques et al., 2023; Sun et al., 2024). While numerous studies have explored plant adaptation strategies to climate change, most focus on large-scale patterns, such as vegetation (Saladin et al., 2020; Antão et al., 2022; Cabon et al., 2022; Mirabel et al., 2023; Pu et al., 2025) and forest phenology (Piao et al., 2019; Shen et al., 2022), with few investigating species-specific adaptability (Weih, 2009; Repo et al., 2021; Martinez Del Castillo et al., 2022; Zlobin et al., 2024). In-depth research at the species level is essential for understanding how plants adjust their survival strategies to rapidly changing climatic conditions, which is crucial for elucidating their survival mechanisms and ensuring biodiversity conservation (Ram, 2024). As a globally recognized biodiversity hotspot, the Qinghai-Tibet Plateau exhibits heightened sensitivity to climate change (Meng et al., 2023; Tang et al., 2023). Ecological adaptation studies in this region not only enrich plateau ecological theories but also provide a scientific foundation for biodiversity conservation in high-altitude ecosystems (Pu et al., 2025; Sun et al., 2025). Key adaptive strategies in alpine plants include specialized leaf morphology and physiology, such as smaller leaf surface areas, thicker cuticles, and modified stomatal patterns, which reduce water loss and mitigate solar radiation (Jin et al., 2024). The development of glandular and non-glandular trichomes on leaves also plays a vital role in enhancing survival, as these structures can enhance the plant’s ability to withstand various stresses (Dong et al., 2023). Additionally, the trade-off between trichome density and chemical defenses may have significant implications for plant survival strategies in resource-limited environments (Xu et al., 2025). These strategies not only ensure plants survival in harsh alpine conditions but also contribute to biodiversity across the diverse habitats of the plateau. Investigating these adaptations provides insights into how plants face climate change challenges and supports high-altitude ecosystem conservation.

As a prominent shrub of the Qinghai-Tibet Plateau (Gao et al., 2015), *Rosa sericea* is widely recognized for its extraordinary phenotypic plasticity (Ullah et al., 2022a; Jiao et al., 2023), a key factor in maintaining the ecological balance of the region. During the spring and summer months, *R. sericea* undergoes vigorous growth, followed by a physiological dormancy phase in autumn. This dormancy acts as an adaptive strategy, shielding the plant from imminent environmental extremes. However, climate change on the Qinghai-Tibet Plateau (Li et al., 2019) has lengthened the period of autumn warmth, disrupting the natural growth patterns of this plant (Wang et al., 2022b; Wu et al., 2022; Sigdel et al., 2024). Recent field studies have shown that *R. sericea* often initiates a second vegetative growth cycle during the warm autumn, sprouting new shoots in the same year. This characteristic makes *R. sericea* a potentially excellent model for studying how climate change modifies plant adaptation strategies. Although initial research has focused on leaf morphology and metabolomics (Ullah et al., 2022b; Jiao et al., 2023), a deeper understanding of its developmental mechanisms at the cellular and molecular levels remains lacking, limiting our knowledge of how *R. sericea* adapts to its environment.

The emergence of single-cell RNA sequencing (scRNA-seq) technology has revolutionized the study of biological phenomena at the resolution of individual cells (Qin and Tape, 2024; Lee et al., 2025). In recent years, scRNA-seq has provided significant insights into the development of complex tissues (Jovic et al., 2022; Wang et al., 2023; Conte et al., 2024; Zhu et al., 2025), greatly enhancing our understanding of cellular identity (Liu et al., 2023; Ye et al., 2024; Guo et al., 2025), organ heterogeneity (Zhang et al., 2023; Marchant and Walbot, 2025), gene regulation (Guo et al., 2025; Liang et al., 2025), and cellular metabolic networks (Li et al., 2023; Wu et al., 2024b). In this study, we applied snRNA-seq to *R. sericea* tender leaves, mapping its transcriptional landscape and uncovering the cellular-genetic mechanisms underlying leaf adaptive development. To harness this potential at single-cell resolution, we defined the core objectives of this study as follows:(1) How is cellular heterogeneity organized in *R. sericea* leaves, and what are the developmental lineages of major cell types? (2) Which transcriptional programs and cell types show signatures of being shaped by or responding to high-altitude stresses? (3) What is the regulatory architecture contributing key adaptive traits, such as trichome development, and how does it integrate developmental cues with potential environmental signals? Through this work, we aim to (i) contribute to a cell-resolved understanding of leaf adaptive development in an alpine shrub, (ii) explore potential links between molecular networks and ecologically relevant phenotypes, and (iii) offer testable hypothesis model for future functional and evolutionary studies.

## 2 Materials and methods

### 2.1 Plant material collection and preparation

**Figure 1:**
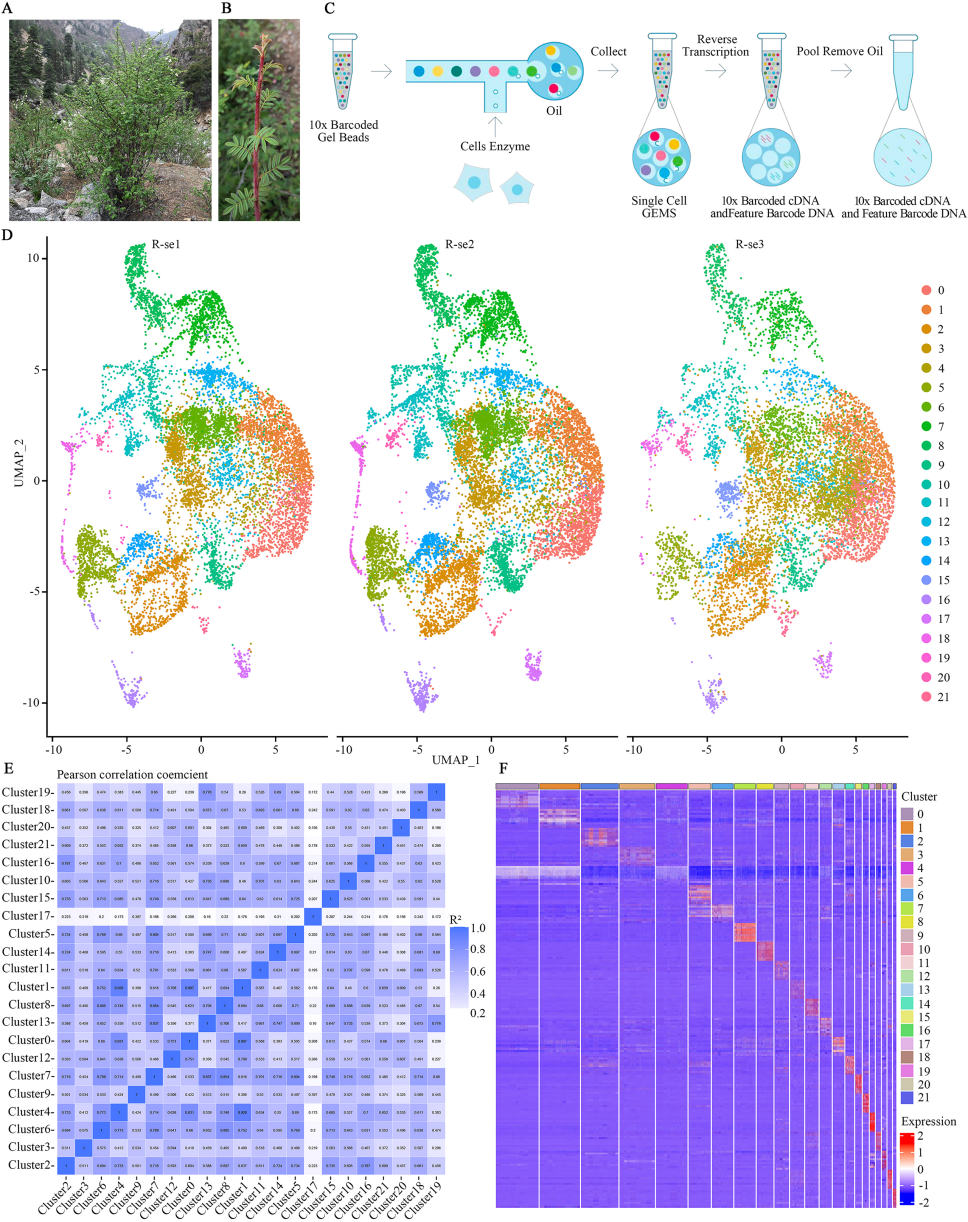
Single-cell sequencing library construction process and cell clustering of *R. sericea*. **(A)** Habitat and flowering plants of *R. sericea*. **(B)** Tender autumn leaves of *R. sericea*. **(C)** Overview of the scRNA-seq library construction process for *R. sericea* leaves (workflow diagram adapted from https://grcf.jhmi.edu/service/10x-single-cell/). **(D)** UMAP plot showing the clustering of leaf cells, with each dot representing an individual cell. The 22 distinct colors correspond to 22 specific cell clusters. Replicates are indicated as R-se1, R-se2, and R-se3. **(E)** Pearson correlation analysis between cell clusters. Each square represents the correlation coefficient between two clusters. Darker colors indicate higher correlation (values closer to 1). **(F)** Heatmap of the top 10 DEGs across 22 cell clusters. The horizontal axis represents cell populations, and the vertical axis denotes the DEGs for each population. Color intensity reflects gene expression levels, with red indicating higher expression and blue indicating lower expression.

Tender leaves of *R. sericea* from the same individual autumn shoots (**Figures 1A, B**), distinct from the spring leaves, were collected in triplicate on September 28, 2023, at 2,518 meters, on Erlang Mountain (Luding County, Sichuan Province, China). The leaves were immediately preserved by rapid freezing in liquid nitrogen to maintain cellular integrity.

### 2.2 Nucleus isolation protocol

The nucleus isolation protocol was adapted from a previously published study (Conde et al., 2021). Frozen tissue was carefully ground into small pieces in liquid nitrogen using a mortar and pestle, and then transferred to a gentleMACS M tube (MiltenyiBiotec) containing 5 mL of Nuclei Isolation Buffer (NIB) (5% Dextran T40, 0.4M sucrose, 10 mM MgCl2, 1 mM DTT, 0.1% Triton X-100, 2U/μl Protector RNase Inhibitor, 100 mM Tris-HCl pH 7.4). The resulting suspension was filtered through a 70-µm mesh strainer and centrifuged at 300g for 1 minute. The pellet was resuspended in 500 µL NIB, filtered again through a 40-µm strainer, and subsequently resuspended in Wash Buffer (10 mM PBS, 1% BSA, 2U/μl Protector RNase Inhibitor). To minimize contamination from organelles such as chloroplasts and to remove cellular debris that could potentially clog microfluidic chips, the nuclei were sorted by gating on the DAPI peaks using a BD FACS Aria III (BD Biosciences). The sorted nuclei were stained with 10 µM DAPI for 5 minutes to assess their integrity and concentration.

### 2.3 Construction and sequencing of snRNA-seq libraries

The snRNA-seq libraries were constructed using the Chromium Next GEM Single Cell 3’ GEM, Library & Gel Bead Kit v.3.1, following the instructions provided in the user manual (**Figure 1C**). Additionally, three separate preparations of single-nucleus suspensions, derived from tender leaves of the *R. sericea* shrub, were sequenced to represent three biological replicates.

After completing the library preparation, quality control was performed. Initial quantification was done using the Qubit 2.0 fluorometer, followed by assessment of the insert DNA size with the Agilent 2100 Bioanalyzer. Once the insert size met the expected criteria, the effective library concentration (2nM) was accurately quantified using qPCR to ensure the library’s quality. After passing quality control, the libraries were sequenced on the Illumina NovaSeq 6000 platform using 150 bp paired-end reads. The sequencing depth of each sample was at least 100G.

### 2.4 SnRNA-seq data preprocessing

Before bioinformatics analysis, the raw sequencing data were assessed for quality using FastQC. The data were then subjected to several quality control and filtering steps to generate clean data for further analysis.

The Cell Ranger software v3.1.0 from 10× Genomics was utilized to align sequencing reads to the reference genome, assess cell numbers, and filter cells (Zheng et al., 2017). Initially, we performed read alignment using STAR (Dobin et al., 2013) to map the sequencing reads to the *R. sericea* reference genome (CNA0146539). The uniquely aligned reads were then quantified to determine UMI types and generate the UMI-barcode expression matrix. Subsequently, cellular and non-cellular barcodes were distinguished by analyzing variations in unique molecular identifiers (UMIs) counts, which are indicative of gene expression levels across individual barcodes, which were assigned as candidate cell identifiers.

To ensure the retention of high-quality cells, the following criteria were applied: cells with fewer than 20% mitochondrial genes, cells expressing more than 200 genes, total expression levels greater than 400, and predicted doublet scores below 0.25, using the Python package Scrublet (Wolock et al., 2019). This filtering process successfully eliminated low-quality cells as well as doublets and multiplets. After applying these quality control criteria, 31,796 high-quality single cells were retained for subsequent analyses.

### 2.5 Identification of highly variable genes, cell clustering, and visualization

The Seurat v3 software (Stuart et al., 2019) was used to perform cell clustering, dimensionality reduction and differential gene expression analysis. We applied “FindIntegrationAnchors” function and “IntegrateData” function to remove the batch effects between samples. The matrix was standardized using the LogNormalize method from the “Normalization” function. To identify HVGs, the “Variance Stabilizing Transformation” method was applied in the “Find Variable Features” function, and the top 2,000 HVGs were selected. Cell clustering was performed using the “Find Clusters” function, with a weighted graph-based method called Shared Nearest Neighbour (SNN). For cell visualization, t-distributed stochastic neighbor embedding (t-SNE) was applied in Seurat (Kobak and Berens, 2019), and the results were concurrently validated using uniform manifold approximation and projection (UMAP) software (Becht et al., 2018).

### 2.6 Tissue-specific marker gene identification and leaf cell-type annotation

Orthologous gene alignments of published leaf-related marker genes from the model plant *Arabidopsis thaliana* were utilized to identify cell-type-specific markers in *R. sericea*. Specifically, the detailed sequences of these marker genes were retrieved from the Plant Cell Marker Database (http://www.tobaccodb.org/pcmdb/) (Jin et al., 2022). Additionally, data from PlantscRNAdb (http://ibi.zju.edu.cn/plantscrnadb/#/) (Chen et al., 2021) and scPlantDB (https://biobigdata.nju.edu.cn/scplantdb/home) (He et al., 2024) were integrated for further supplementation and cross-verification. Using the *A. thaliana* (TAIR10) marker genes as query sequences, homologous genes in *R. sericea* were identified using the BLAST v2.12.0 (Camacho et al., 2009). The top-scoring hits were selected and annotated as corresponding *R. sericea* cell-type-specific genes.

### 2.7 Cross-species mapping to *Arabidopsis* leaf snRNA-seq reference

To assess the robustness of cell type annotation without additional experimental validation, we conducted a cross-species transcriptomic comparison by mapping *R. sericea* leaf snRNA-seq data to a published *A. thaliana* leaf snRNA-seq reference atlas. We used the Rosette S3 dataset from a recent high-resolution study (Guo et al., 2025), which includes well-characterized leaf cell populations comparable to our annotations. Putative orthologs were identified by BLAST, retaining best-hit gene pairs with the highest bit score. Highly variable genes were defined independently in both datasets using Seurat (Stuart et al., 2019). Cross-species similarity was evaluated using Spearman’s rank correlation coefficient and visualized as a heatmap in R.

### 2.8 High-dimensional weighted gene co-expression network analysis

The hdWGCNA package (Morabito et al., 2023) in R (https://smorabit.github.io/hdWGCNA/) was employed for gene co-expression analysis. A weighted adjacency matrix was constructed using an unsupervised hierarchical clustering method (Morabito et al., 2021, 2023), with the optimal soft threshold power (β) was set to be 6 to assess the scale-free topology. Modules were identified based on the following parameters: gene_select = “fraction,” fraction = 0.05, nearest-neighbors parameter (k) = 50, minModuleSize = 50, and max_shared = 10. Significant co-expression modules were defined when the Pearson correlation coefficient was greater than 0.40 and the p-value was below 0.05. Finally, the co-expression network of genes was visualized using Cytoscape software v3.9.1 (Shannon et al., 2003).

### 2.9 Gene ontology enrichment analysis and gene set enrichment analysis

The clusterProfiler R package was employed to perform the enrichment analyses (Yu et al., 2012). GO enrichment analysis (Gene Ontology, 2008) was conducted on differentially expressed genes (DEGs) to identify their potential functional enrichments. Based on Module Eigengene Connectivity (kME), hub genes identified from the hdWGCNA analysis were selected for the GSEA (Subramanian et al., 2005). Reference gene sets, such as the target gene sets (G6) from the PlantGSAD database were utilized (Ma et al., 2022). The parameters for the enrichment analysis were configured as follows: pAdjustMethod = ‘BH’, pvalueCutoff = 1, and qvalueCutoff = 1.

### 2.10 Pseudotime trajectory analysis

Differentiation trajectories of all leaf cells and leaf epidermal cells were analyzed using the Monocle 2 v2.26.0 R package to investigate their pseudotime relationships (Qiu et al., 2017). The “dpFeature” function was used to identify genes that define specific biological processes. Clustering analysis was performed using the “reduce Dimension ()” function, with max_components = 2 and reduction_method = “DDRTree.” The differentiation trajectories were then inferred using the “orderCells” function with default parameters (Qiu et al., 2017). Visualizations of gene expression were generated using the specialized pseudotime trajectory function to track changes in gene expression throughout differentiation stages. This method was also applied to several marker genes relevant to developmental pseudotime. Branch-dependent differentially expressed genes were identified using the BEAM function.

### 2.11 Construction of overexpression vector and plant transformation

Total RNA was isolated from fresh *R. sericea* leaves, and the full-length coding sequence (CDS) of the *RO6G37307* gene was amplified through reverse transcription. The overexpression vector was constructed via homologous recombination. A constitutive 35S promoter-driven overexpression vector was utilized for cloning. After homologous recombination, 5 µL of the recombinant product was introduced into *Escherichia coli*, which was then plated on LB agar containing 100 mg/L spectinomycin for colony selection. Four single colonies were picked for PCR verification, and plasmids were extracted from those with correct sequencing results. Subsequently, the *Agrobacterium*-mediated floral-dip method was applied to introduce the overexpression construct into *A. thaliana* (Columbia ecotype, Col-0) at the bolting stage. Plants were dipped three times at 5–7 day intervals to improve transformation efficiency. Seeds collected from the T0 generation were sown on kanamycin-containing medium to select positive transformants.

### 2.12 Selection of high-expression lines and phenotypic evaluation

After obtaining the resistant lines through selection for kanamycin resistance, total DNA and RNA were extracted from the candidate plants using the BGMG Fast Total RNA/DNA Co-Extraction Kit. The resistant lines were then subjected to PCR and RT-qPCR validation. Stable *RO6G37307* overexpression lines were established in the T2 generation. The leaf surface trichome of the *RO6G37307*-OE lines were imaged in three biological replicates using a digital camera (Nikon D750) and scanning electron microscopy (SEM) at three positions (base, mid, apex) on the 3rd and 5th rosette leaves, and the 1st cauline leaf. Each leaf section measured 5 mm × 5 mm, and the trichome density was compared to that of the Col-0 ecotype.

### 2.13 Protein functional analysis

Homologous amino acid sequences corresponding to the RO6G37307 protein were retrieved from the NCBI database and filtered according to the following thresholds: E-value ≤ 1 × 10^-5^, sequence identity ≥ 50%, and query coverage ≥ 70%. A phylogenetic relationship was inferred using the neighbor-joining (NJ) method implemented in MEGA v12.0 under the p-distance model, with 5,000 bootstrap replicates to assess node support; all other parameters were retained at their default values (Kumar et al., 2024b). Signal peptide prediction was conducted using SignalP v6.0 under default settings (Teufel et al., 2022), while transmembrane domain topology was assessed via DeepTMHMM v1.0 with default configurations (Hallgren et al., 2022).

For experimental subcellular localization, the full-length CDS of the *RO6G37307* gene was fused in-frame to the enhanced green fluorescent protein (EGFP) within the pBI121 vector. The recombinant construct was introduced into one-month-old *Nicotiana benthamiana* leaves through *Agrobacterium*-mediated transient transformation (strain GV3101 carrying the pSoup-p19 helper plasmid). The infiltrated leaves were cultured at room temperature in darkness for 36 to 48 h. Leaf segments were then excised and mounted on glass slides. To increase the visibility of the plasma membrane and cell wall interface, samples were treated with 1 M mannitol solution for approximately 10 minutes to induce plasmolysis. The intracellular distribution of the protein was observed by observing EGFP fluorescence using a confocal laser scanning microscope.

## 3 Results

### 3.1 Construction of a high-resolution single-cell transcriptomic atlas of *R. sericea* leaves

To establish a comprehensive single-cell atlas capturing the developmental dynamics of autumn leaves in *R. sericea* for reflecting climate-induced secondary growth. we collected tender leaves from autumn shoots (**Figures 1A, B**), isolated nuclei, and performed snRNA-seq using the 10× Genomics Chromium platform. Following the standard snRNA-seq workflow (**Figure 1C**), we generated transcriptomic profiles from 9,368, 10,434, and 12,156 single cells across three independent biological replicates, which were subjected to rigorous quality control. On average, approximately 1,100 genes and 1,900 UMIs were detected per cell (**Supplementary Table S1**). After filtering out low-quality cells and potential doublets, 9,365, 10,357, and 12,074 high-quality single-cell transcriptomes were retained, yielding a final dataset of 31,796 cells for downstream analysis (**Supplementary Table S2**).

For cell population identification, we performed batch effect correction and integrated the three replicates. This analysis resolved 22 distinct cell clusters, which were visualized by UMAP (**Figure 1D**) and validated with t-SNE (**Supplementary Figure S1**). The clustering results were highly reproducible among replicates, demonstrating minimal batch effects and robust data quality. Furthermore, differential gene expression analysis and inter-cluster Pearson’s correlation further confirmed the reliability and biological relevance of the clustering results (**Figures 1E, F**).

### 3.2 Classification and identity annotation of leaf cells

**Figure 2:**
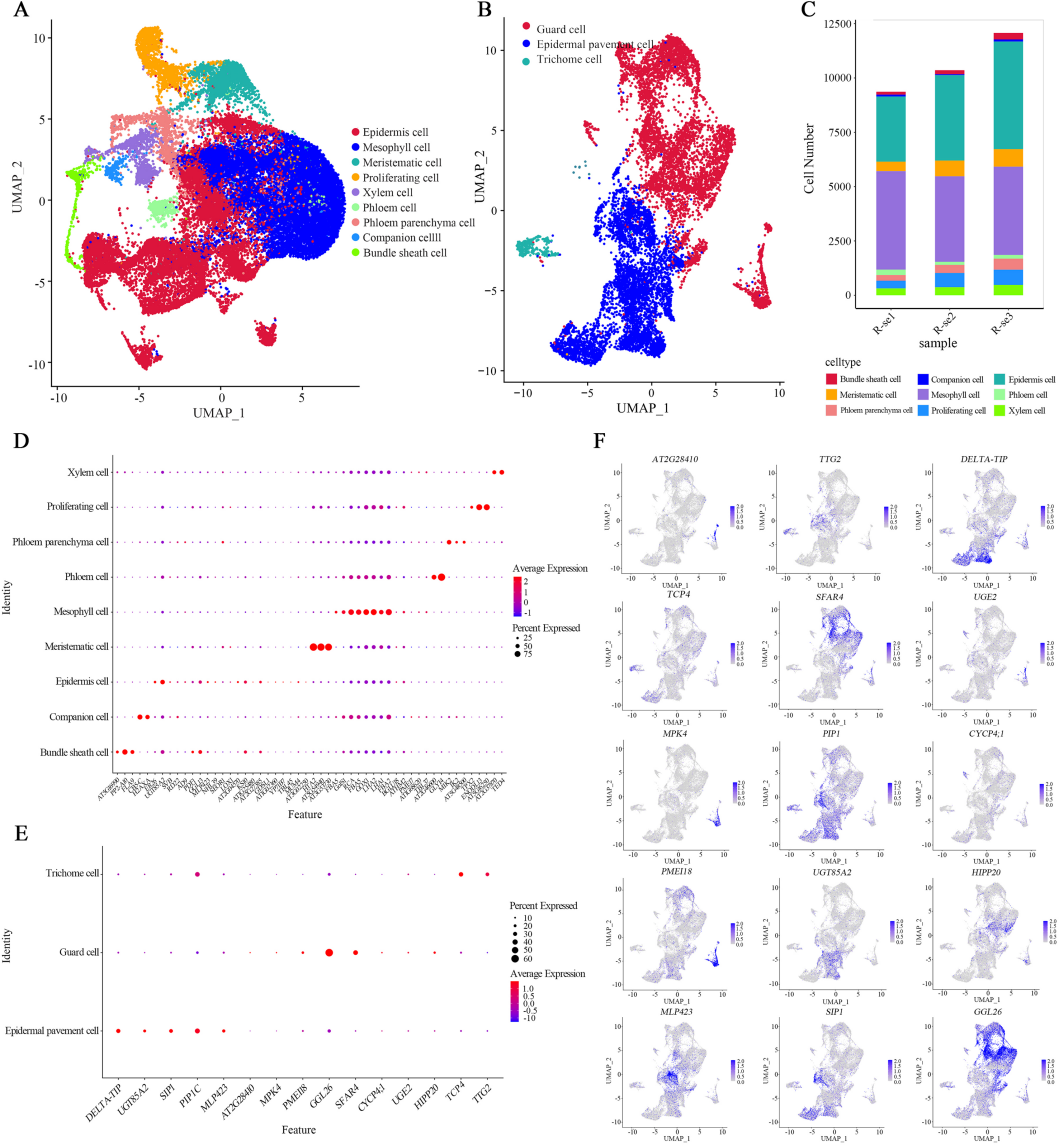
Cell-type annotation of *R. sericea* leaves. **(A, B)** UMAP embeddings of all single cells; each point represents one cell and is colored by the assigned cell type. **(C)** Bar chart showing cell counts per type; colors match the palette in panels. **(D, E)** Dot plots of canonical marker genes across cell types; dot size indicates the fraction of cells expressing each gene, and color encodes the average scaled expression (warmer colors indicate higher expression). **(F)** Subclustering and re-annotation of the epidermal lineage by 15 marker genes, highlighting pavement, guard, and trichome subtypes on UMAP.

To obtain reliable annotations, we first performed GO enrichment analysis on each cell cluster, aiming to infer their potential cellular identities based on biological functions. For instance, Clusters 0, 1, 4, 6, and 12 were predominantly enriched for chloroplast-related and photosynthesis functions (**Supplementary Figure S2**), suggesting their potential identity as mesophyll cell. Subsequently, we examined the top 50 cluster-specific genes and compared them with previously reported marker genes in *A. thaliana* (**Table 1**; **Supplementary Table S3**, **Supplementary Figure S3**). Furthermore, we analyzed the spatial expression patterns of 52 specific marker genesacross 22 cell clusters to more intuitively assess gene-specific expression and aid in leaf celltype annotation. (**Supplementary Figure S4**) This approach enabled the preliminary annotation of nine major cell types: mesophyll, epidermis, meristematic, proliferating, phloem, phloem parenchyma, xylem, bundle sheath, and companion cells (**Figures 2A, C, D**; **Supplementary Figure S5**).

**Table 1:** Marker genes for celltypes annotation of *R. sericea* leaf.

Cell types	Marker genes	References
Mesophyll cell	*FBA5, GolS1, GOX1, LHCA2, LHCA1, LHB1B2, FBA2, RCA, BGLU18, ATHM2, PME17, AT1G68620, TBL37*	(Kim et al., 2021; Xu et al., 2021; Zhang et al., 2021; Liu et al., 2022a, b; Procko et al., 2022; Tenorio Berrio et al., 2022)
Epidermis cell	*CER26, SVB, UGT85A2, RD22, CAD9, PDF1, GGL13, MLP423, NHL39, SEOR1, LOX1, GGL14, KCS10, ALMT4, AT2G27385, GDSL1, AT1G02360, CYP71B7, TBL45, MUM4, RRT2*	(Liu et al., 2020; Kim et al., 2021; Lopez-Anido et al., 2021; Zhang et al., 2021; Procko et al., 2022; Tenorio Berrio et al., 2022; Xia et al., 2022)
Bundle sheath cell	*AT5G16990, FLA9, CB5-C*	(Kim et al., 2021; Xu et al., 2021; Procko et al., 2022; Tenorio Berrio et al., 2022)
Phloem parenchyma cell	*MIK2, CIK2, NOI9*	(Kim et al., 2021; Liu et al., 2022b; Procko et al., 2022; Tenorio Berrio et al., 2022)
Phloem cell	*AT2G46600, GLYI4*	(Liu et al., 2022b)
Xylem cell	*AT2G37870, TED4*	(Liu et al., 2022a, b)
Companion cell	*PP2-A10, AT5G54940*	(Kim et al., 2021; Zhang et al., 2021; Procko et al., 2022)
Meristematic cell	*HTA2, H2AXA, AT3G53730*	(Zhang et al., 2021; Liu et al., 2022a, b)
Proliferating cell	*TPX2, ENODL13, AT5G16250*	(Lopez-Anido et al., 2021; Zhang et al., 2021; Procko et al., 2022; Tenorio Berrio et al., 2022)
Guard cell	*AT2G28410, MPK4, PMEI18, GGL26, SFAR4, CYCP4;1, UGE2, HIPP20*	(Kim et al., 2021; Lopez-Anido et al., 2021; Xu et al., 2021; Zhang et al., 2021; Procko et al., 2022; Tenorio Berrio et al., 2022; Xia et al., 2022)
Epidermal pavement cell	*DELTA-TIP, UGT85A2, SIP1, PIP1C, MLP423*	(Kim et al., 2021; Zhang et al., 2021; Procko et al., 2022; Tenorio Berrio et al., 2022)
Trichome cell	*TCP4, TTG2*	(Johnson et al., 2002; Pesch et al., 2014; Vadde et al., 2019; Wang et al., 2019; Wei et al., 2019)

As the outermost interface between plants and the aerial environment, the leaf epidermis provides a more sensitive indicator of environmental changes. However, the epidermal cell populations remained insufficiently resolved based on the first round of annotation results, which limits our understanding of the plateau adaptation of *R. sericea*. Therefore, we reanalyzed this subset to achieve higher resolution. A total of 11,888 epidermal cells were reclustered into 23 subclusters, which were subsequently putatively annotated as epidermal pavement, guard, and trichome cells (**Figures 2B, E, F**).

Integrating the results from both rounds of annotation, we identified 11 distinct leaf cell types: mesophyll, meristematic, proliferating, phloem, phloem parenchyma, xylem, bundle sheath, companion, epidermal pavement, guard, and trichome cells. This refined classification highlights the cellular diversity of *R. sericea* leaves and serves as a framework for downstream functional analyses.

### 3.3 Cross-species comparison supports the overall plausibility of cell type annotation

To evaluate the consistency of our cell type annotations, we compared the *R. sericea* leaf snRNA-seq dataset with a published *A. thaliana* leaf snRNA-seq reference atlas using a cross-species transcriptomic similarity analysis. Based on orthologous highly variable genes, average expression profiles were calculated for each annotated cell type, and absolute Spearman rank correlation coefficients (r_s_) were computed between different cell types of two species. Overall, several biologically corresponding cell type pairs showed modest but statistically significant expression similarity across species (r_s_ ≥ 0.24, p < 0.01), including companion, bundle sheath, xylem, mesophyll, and epidermis-related cells. Although the strength of correlation varied among cell types, the overall correspondence pattern was broadly consistent with expectations derived from marker-based annotations. Notably, certain cell populations displayed partial overlap with multiple *A. thaliana* reference types or relatively weaker similarity signals (**Supplementary Figure S6**). The relatively modest absolute correlation values observed here highlight both species-specific transcriptional programs, evolutionary divergence, and developmental context between the two datasets. Together, these results provide additional, transcriptome-level support for the plausibility of the assigned cell identities, while acknowledging inherent limitations associated with cross-species comparisons.

### 3.4 Gene ontology analysis reveals functional diversity across cell types

Beyond marker gene annotation, functional enrichment provides an additional layer of evidence for cell identity. To examine the classification and explore biological specialization, we performed GO enrichment analysis on the DEGs from each cell type. The enriched GO terms aligned closely with expected functional roles, with results consistent with the annotation and informative for adaptive processes (**Supplementary Figure S7**).

Specifically, mesophyll cells were enriched for photosynthesis-related categories such as “photosynthetic process” (GO:0015979) and “chloroplast thylakoid membrane” (GO:0009535). Meristematic and proliferating cells were associated with GO terms related to cell cycle progression and chromatin organization (e.g., GO:0000786, GO:0046982, GO:0007067, GO:0051301). Vascular cell types showed enrichment in hormone signaling (GO:0009734), solute transport (GO:0006820, GO:0008509, GO:0080161), and secondary cell wall biogenesis (GO:0045492). Trichome cells were characterized by terms related to responses to biotic and abiotic stresses, including oxidative stress and defense responses (GO:0031640, GO:0006970, GO:0006805). Guard cells exhibited enrichment in fatty acid biosynthesis (GO:0006633), carboxylic ester hydrolase activity (GO:0052689), and lipid metabolic processes (GO:0044255). Notably, epidermal pavement cells displayed enrichment in photosynthesis-associated categories, including “thylakoid membrane” (GO:0042651), “chloroplast thylakoid membrane” (GO:0009535), “photosynthesis, light harvesting” (GO:0009765), and “response to far-red light” (GO:0010218), in addition to cell wall organization (GO:0009505). This strongly suggests that presence of transcriptionally active nuclear-encoded genes involved in chloroplast function in epidermal pavement cells of *R. sericea.*

### 3.5 Gene co-expression network analysis identifies key regulatory modules

**Figure 3:**
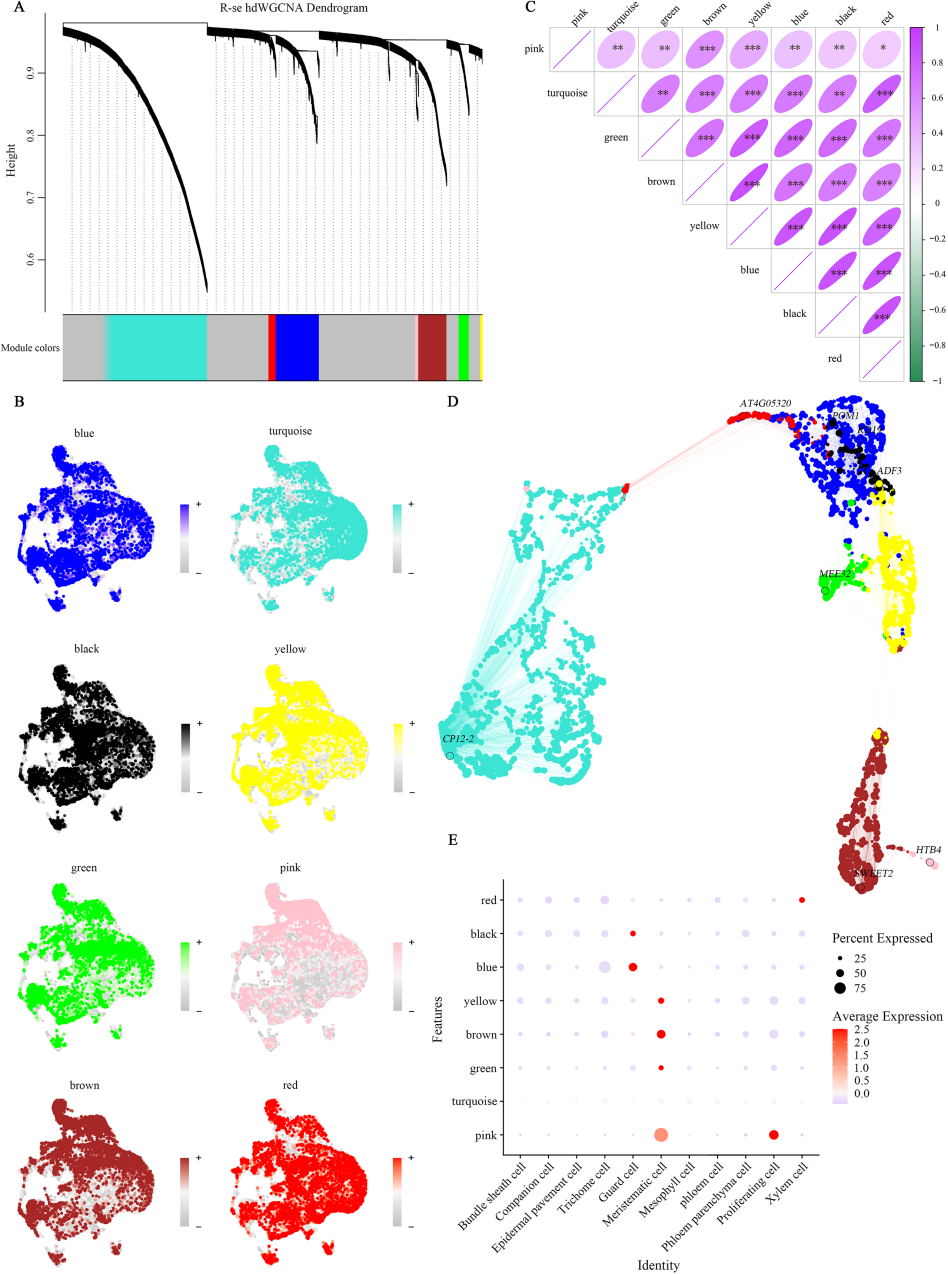
The hdWGCNA analysis of *R. sericea* leaf cells. **(A)** Dendrogram of gene clustering. Each branch represents an individual gene, with color coding at the bottom indicating the gene’s association with specific co-expression modules. The “grey” module represents genes that were not assigned to any specific co-expression module. **(B)** UMAP projections of the harmonized module eigengenes (hMEs) for the eight modules. **(C)** Correlation analysis between the eight modules (*p < 0.05, **p < 0.01, ***p < 0.001). **(D)** UMAP plot displaying the top hub gene from each module. **(E)** Bubble plots showing the performance of hMEs from each module across different cell types. The dot plot illustrates the average expression of module-specific Module Eigengenes (ME) across different cell clusters, ranked by their kME values within each module.

hdWGCNA was applied to systematically uncover coordinated gene expression programs across all cell types. Using 7,574 DEGs, we identified eight distinct co-expression modules (R-se1 to R-se8) characterized by unique expression profiles (**Figures 3A–D**). Among these, the gray module comprised unassigned genes, whereas the turquoise module was the largest (1,687 genes) and the pink module was the smallest (53 genes). Correlation analysis between modules and cell-type abundance revealed specific associations. For instance, the yellow, brown, green, and pink modules were strongly enriched in meristematic cells, whereas black and blue modules correlated with guard cells, and the red module was specific to xylem cells. Interestingly, the pink module was simultaneously associated with both meristematic and proliferating cells, suggesting overlapping transcriptional programs regulating early growth. In contrast, the turquoise module exhibited no strong correlation with any single cell type (**Figure 3E**), which may indicate that these are housekeeping or universally regulated genes. These results suggest the presence of both cell-type-specific and cross-lineage regulatory modules. Modules enriched in proliferative or stress-related cell types may represent key transcriptional programs driving adaptive leaf development in *R. sericea*.

### 3.6 GSEA suggests a potential association between SPL7-related transcriptional programs and copper homeostasis

**Figure 4:**
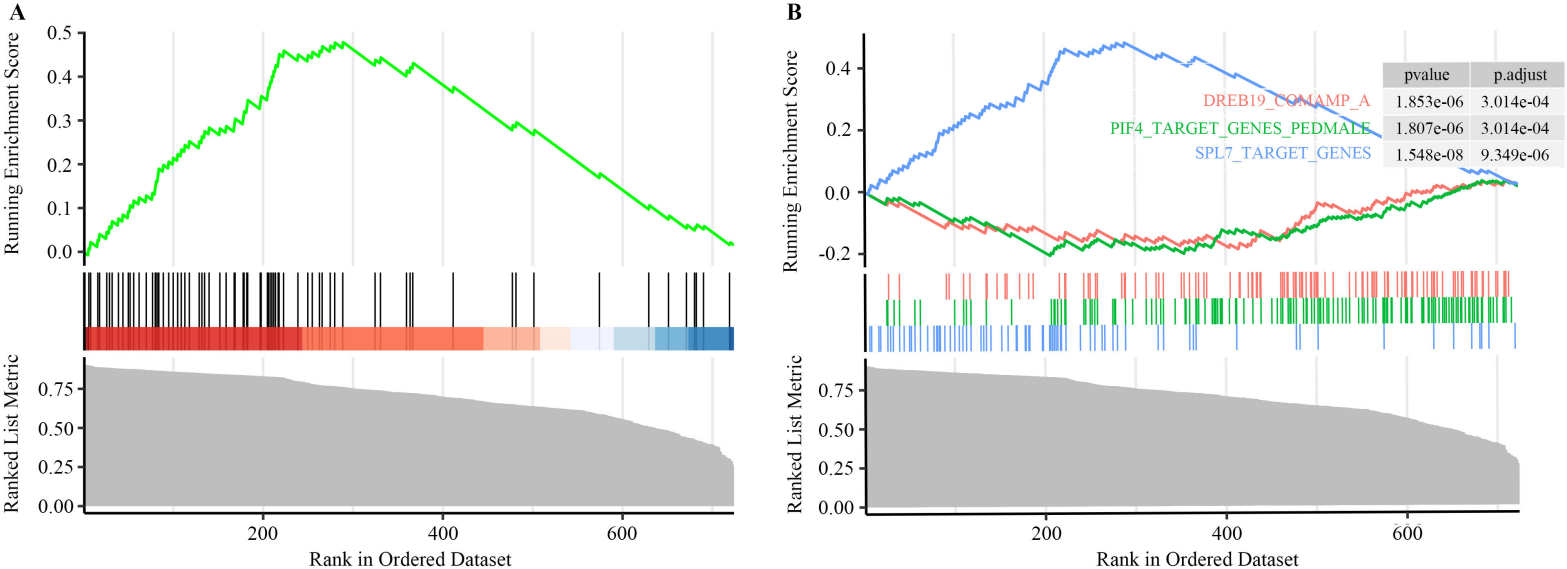
GSEA analysis based on target gene sets during *R. sericea* leaf development. **(A)** Gene set enrichment plot. The upper section displays the enrichment score. Vertical lines in the middle indicate the position of each gene within the ranked gene set list. The lower section shows the distribution of gene ranks, represented as a gray area plot. **(B)** The top 3 gene sets with the highest enrichment scores. Gene set names, p-values, and adjusted p-values are shown in the upper right corner.

To further elucidate regulatory processes at the species level, we performed GSEA on hub genes identified from the hdWGCNA modules. A total of 724 hub genes were analyzed (**Supplementary Table S4**). The analysis revealed a significant enrichment of the SPL7_TARGET_GENES set (p-value = 1.548e-08), with an enrichment score (ES) of 0.45 and a normalized enrichment score (NES) of 2.94. This set contained 64 genes, including 57 core-enriched members that contributed strongly to the signal (**Figures 4A, B**). Squamosa promoter-binding protein-like 7 (SPL7), a member of the SBP transcription factor family, is a pivotal regulator of copper homeostasis in plants (Yan et al., 2017; Mermod et al., 2019).

### 3.7 Global pseudotime analysis of leaf development

**Figure 5:**
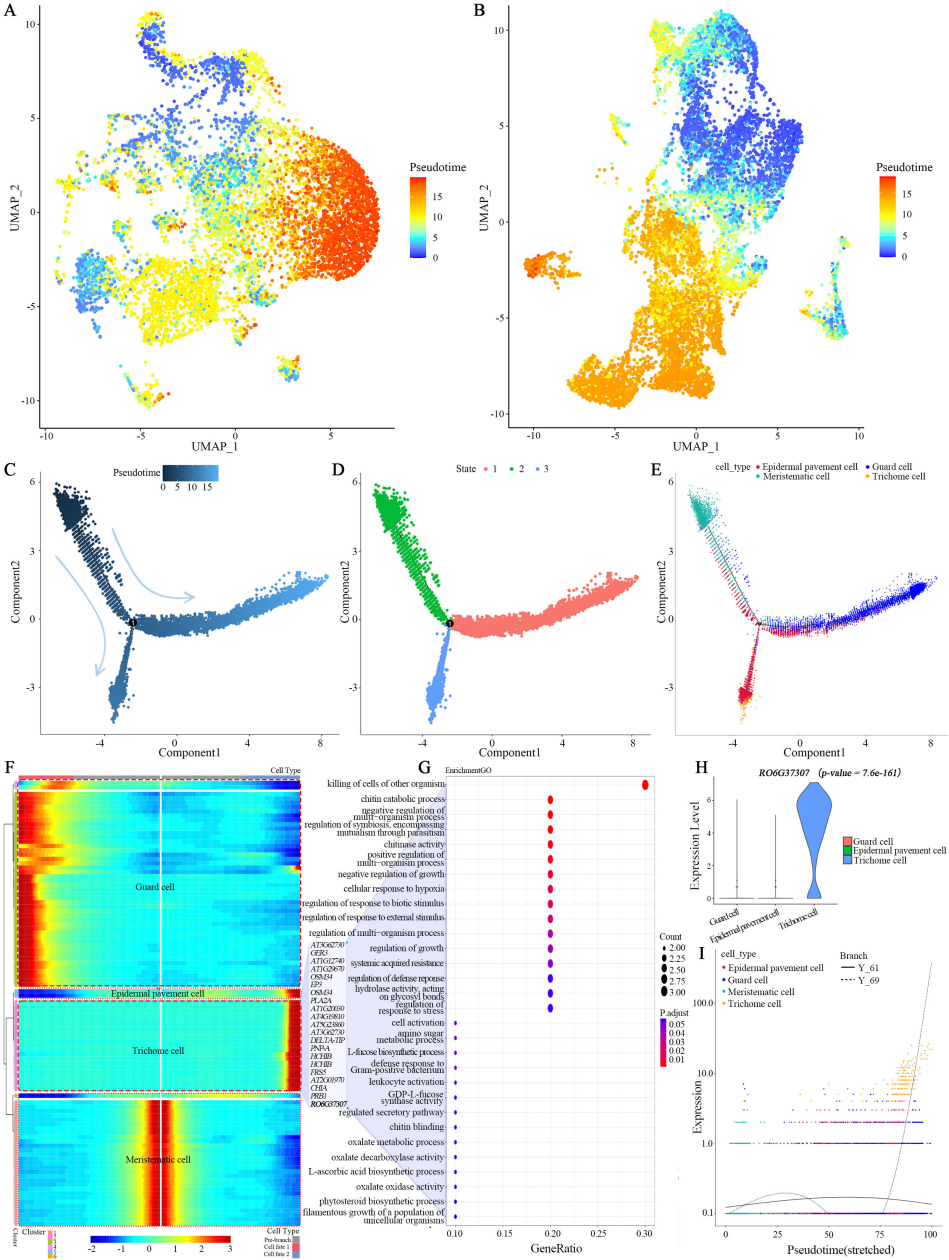
Pseudotemporal differentiation and development of *R. sericea* leaves. **(A)** Global pseudotemporal annotation of leaf cells, mapping the “pseudo-time” of Monocle 2 onto the two-dimensional UMAP space. **(B)** Pseudotemporal annotation of leaf epidermal cells, mapping the “pseudo-time” of Monocle 2 onto the two-dimensional UMAP space. **(C)** Ordering of leaf epidermal cells along pseudotemporal differentiation trajectories. **(D)** States of leaf epidermal cells along a pseudotemporal differentiation trajectory. **(E)** Distribution of four cell types in the leaf along pseudotemporal differentiation trajectories. **(F)** Heatmap showing the top 100 genes with the most significant changes along the differentiation trajectory. The branch point in the middle indicates the start of pseudo-time. The topology used to visualize pseudotemporal development in the four cell types is outlined with red dashed line in the figure. Key genes involved in trichome differentiation are shown on the right, with genes highlighted in bold being the focus of this study. **(G)** GO functional enrichment of trichome fate-determining genes among the top 100 genes along the leaf epidermal differentiation trajectory. **(H)** The violin plot of *RO6G37307* gene expression in *R. sericea* leaf epidermal cell subtypes. **(I)** Expression trends of *RO6G37307* in relation to cell fate determination. The dotted line indicates the expression level changes of *RO6G37307* along the pseudo-temporal differentiation trajectory of trichomes.

To reconstruct the developmental trajectory of leaf cells, we performed pseudotime analysis across all captured cell types by Monocle 2. Meristematic cells were designated as the root of the trajectory, representing the least differentiated state. Differentiation was visualized along a continuum from initiation (blue zone) to completion (red zone) (**Figure 5A**).

Our analysis revealed two distinct regions of differentiation initiation: one originating from the meristematic and proliferating cell clusters, and the other from the epidermal cell population. These dual initiation zones suggest that both internal tissues and epidermal lineages harbor high developmental potential. A striking finding was that the initiation zone of differentiation within the epidermis corresponded to the guard cell cluster (**Figure 5B**). This observation may suggests the presence of a lineage within guard cells sharing characteristics with protodermal or guard mother cells, exhibiting high developmental potential. Moreover, mesophyll cells were predominantly localized at the terminal end of the trajectory, consistent with their status as fully differentiated, photosynthetically specialized cells.

### 3.8 Pseudotime trajectory analysis of trichome differentiation

To investigate the developmental dynamics of trichome lineages, we conducted pseudotime analysis of epidermal cells, including pavement, guard, and trichome cells. we designated meristematic cells as the initial differentiation state and reconstructed the epidermal developmental trajectory. The results showed that the pseudotime trajectory contained a single branch point that separated the epidermal population into three distinct states (**Figures 5C, D**). As expected, meristematic cells population dominated early differentiation and were enriched in the pre-branch state (state 2). After bifurcation, guard cells and epidermal pavement cells diverged into two terminal fates, occupying states 1 and 3, respectively. Interestingly, trichome cells shared a differentiation trajectory with epidermal pavement cells, but were only temporally later than the latter (**Figures 5D, E**).

Branch-dependent analysis revealed that the top 100 DEGs across the three states could be grouped into six expression clusters (**Figure 5F**). Genes in cluster 2 were predominantly associated with trichome differentiation and enriched in defense-related processes, such as “killing of cells of other organisms” (GO:0031640) and “chitin catabolic process” (GO:0006032) (**Figure 5G**). These results suggest that trichome commitment is accompanied by the activation of defense-associated transcriptional programs, reflecting the dual role of trichomes in morphogenesis and stress adaptation (Viterbo et al., 2002).

### 3.9 RO6G37307 positively regulates trichome development in leaf epidermis

Among the top 100 genes showing the most pronounced transcriptional changes along the epidermal differentiation trajectory, we focused on RO6G37307 (a 495-bp gene encoding a 164-amino-acid protein, **Supplementary Table S5**) as a candidate regulator because it showed markedly specific and elevated expression in the trichome cells cluster (**Figure 5H**, p-value = 7.6e-161). Furthermore, during trichome differentiation, its expression pattern was highly coordinated with gene sets associated with trichome biological functions identified based on homologous genes(**Figure 5F**), and its expression timing closely aligned with the differentiation trajectory of trichome cells (**Figure 5I**), suggesting its potential involvement in the trichome development process.

**Figure 6:**
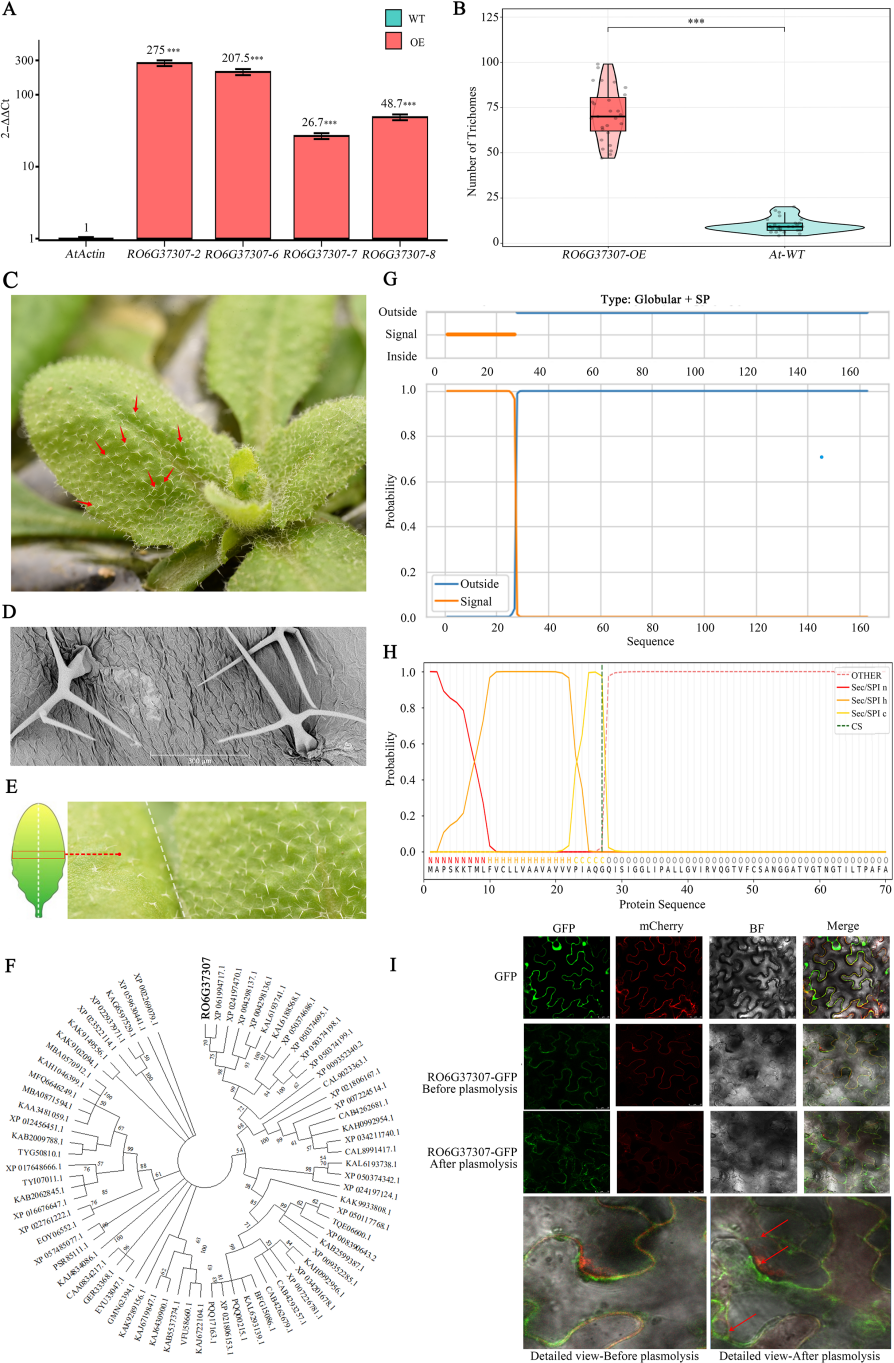
Functional validation of the *RO6G37307* gene. **(A)** Relative expression levels of *RO6G37307* in overexpression (OE) lines. AtActin was used as the internal reference gene for RT-qPCR analysis. Data represent the mean ± SD from three biological replicates, and the relative expression levels are shown above the bars (***p < 0.001). **(B)** Comparison of trichome density on leaf surfaces between the OE line RO6G37307–2 and wild-type (Wt). Data represent the mean ± SD from three biological replicates, and the gray dots represent the recorded data points. The inset shows the three counting areas per leaf (boxed regions) (***p < 0.001). **(C)** Trichome phenotypes on rosette leaves of the OE line RO6G37307-2. Red arrows indicate four-branched trichomes. **(D)** Representative trichome types on adaxial leaf surfaces of OE line RO6G37307–2 under scanning electron microscope. **(E)** Comparative visualization of adaxial leaf trichomes under a digital camera: Wt (left) and RO6G37307-2 (right). **(F)** Phylogenetic analysis of RO6G37307 homologs. Bootstrap support values >50% (from 5,000 replicates) are shown on the branches of the Neighbor-Joining tree. **(G)** Transmembrane domain prediction for RO6G37307. Top: Amino acid sequence of transmembrane helices (orange) and extracellular regions (blue). Bottom: Confidence scores. **(H)** Signal peptide prediction. The blue dotted line indicates the cleavage site at the N-terminus. **(I)** Subcellular localization prediction. In the control, free GFP displayed a uniform distribution in the cell periphery and nucleus. In contrast, RO6G37307-GFP localized to the cell periphery prior to plasmolysis. After plasmolysis, the GFP signal remained at the cell wall (gree) rather than retracting with the plasma membrane (red, as indicated by the arrow), supporting its extracellular secretion and cell wall localization.

To explore its potential biological function, we ectopically overexpressed *RO6G37307* in *A. thaliana*. Overexpression markedly increased trichome density on leaf surfaces—approximately sevenfold higher than that of Col-0 wild type (71 vs. 10 trichomes per unit area)—and also altered trichome morphology, with a subset exhibiting four-branched structures rather than the typical three-branched form (**Figures 6A–E**).

Phylogenetic analysis based on homologous amino acid sequences placed RO6G37307 within a well-supported clade (bootstrap = 98%) alongside Phylloplanin homologs from *Rosa* and *Fragaria*, supporting its membership in the Phylloplanin family (**Figure 6F**). Signal peptide prediction indicated a high-confidence N-terminal cleavage site between residues 27 and 28 (probability = 0.98; **Figure 6H**). Transmembrane topology analysis suggested a “Globular + SP” architecture, lacking transmembrane helices and carrying a cleavable signal peptide typical of secreted, soluble proteins (**Figure 6G**). Finally, subcellular localization assays through GFP fusion experiments showed that RO6G37307 protein is targeted to the extracellular space, and its fluorescence signal is found on the cell wall (**Figure 6I**), consistent with its classification as a secreted phylloplanin-like protein. Together, these results support the role of RO6G37307 as a novel regulator of trichome morphogenesis in *R. sericea* and point to its potential contribution to epidermal defense adaptation.

## 4 Discussion

### 4.1 SnRNA-seq establishes a cellular developmental atlas of a plateau plant in autumn

Elucidating plant developmental programs is essential for understanding evolutionary processes and adaptive strategies (Li et al., 2024). SnRNA-seq provides unprecedented resolution for analyzing cellular heterogeneity during growth and development (Zhu et al., 2022; Ye et al., 2023; Conte et al., 2024), and has revolutionized developmental biology (Nakayama et al., 2022). Applying snRNA-seq to autumn leaves of *R. sericea* offers a unique opportunity to reveal the cellular and molecular mechanisms underlying plant responses to plateau environmental warming.

In this study, we leveraged prior knowledge of *Arabidopsis* marker genes and required that each cluster be annotated with multiple validated marker genes whenever possible, in order to reduce the risk of misannotation through cross-validation. Additionally, we integrated data on the biological functional profiles of the cell clusters and types, and cross-species transcriptomic comparison to provide complementary support for cell type annotation. Using this strategy, we provisionally identified and annotated 11 distinct cell types in *R. sericea* leaves, resulting in a draft cell atlas encompassing major tissues, including leaf epidermis, mesophyll, and vascular systems. The consistency of our annotations with previous single-cell studies further supports their biological plausibility and demonstrates the feasibility of scRNA-seq in non-model alpine plants (Wang et al., 2022a; Zang et al., 2023; Guo et al., 2025). Importantly, this work fills a gap by extending single-cell technologies to autumn shoots of *R. sericea*, thereby providing a valuable resource for exploring adaptive mechanisms in alpine ecosystems. However, Cell type annotation in non-model plant species remains challenging due to the scarcity of species-specific markers and high-quality reference atlases. In this study, cell identities in *R. sericea* were inferred primarily through conserved orthologous markers from *A. thaliana*, complemented by functional enrichment analyses. Although this integrative strategy reduces misannotation risk through cross-validation, it is inherently indirect and inference-based. In addition, snRNA-seq may underrepresent cytoplasmic transcripts, potentially limiting the detection of certain cell-type-specific expression features, while cross-species marker transfer introduces uncertainty due to evolutionary divergence in gene regulation. Therefore, cross-species correlation analysis should be viewed as an auxiliary consistency check rather than a quantitative measure of annotation accuracy. Accordingly, the cell type assignments presented here should be regarded as putative rather than definitive. Future studies combining reference-based cross-species mapping approaches with independent spatial validation methods, such as RNA fluorescence *in situ* hybridization (FISH), will be important for further improving annotation confidence and resolution in *R. sericea* and other non-model plant systems.

### 4.2 Functional heterogeneity and physiological homeostasis of leaf cells underpin adaptability in *R. sericea*

Each plant cell type is not only distinct in identity but also exhibits functional heterogeneity and intricate interconnectedness (Mathe et al., 2021; Thibivilliers and Libault, 2021; Amini et al., 2023). These attributes are fundamental to a plant’s ability to adapt to diverse environmental challenges, sustain normal physiological and developmental processes, and coordinate complex biological activities. Examining the functional dynamics of cell populations under specific environmental conditions provides critical insights into adaptive strategies (Mackenzie and Mullineaux, 2022; Roman et al., 2022; Yu et al., 2024).

*R. sericea* initiates autumnal shoot growth at favorable times, thereby undergoing a secondary phase of vegetative development that effectively extends the growing season. Functional enrichment analyses revealed that autumn leaves of *R. sericea* retained diverse and indispensable biological functions (such as cell cycle progression, photosynthesis, hormone signaling, metabolic processes and defense responses) necessary for maintaining physiological homeostasis, while also exhibiting robust differentiation dynamics (**Supplementary Figure S4**). Previous studies suggested that delayed senescence increases root biomass, though not branch biomass (Zohner et al., 2021). However, it remains uncertain whether extended autumnal growth contributes to greater assimilate accumulation in this species. We speculate that, as a deciduous shrub, *R. sericea* may allocate assimilates gained from autumnal shoots into root storage, potentially forming a reservoir that supports germination and growth in the following spring.

Remarkably, epidermal pavement cells of *R. sericea* harbor transcriptionally active nuclear-encoded genes involved in chloroplast function. Prior studies have demonstrated that chloroplasts in pavement cells not only perform photosynthesis (Barton et al., 2016), but also contribute to immune responses (Irieda and Takano, 2021; Irieda, 2022). Thus, we propose that epidermal pavement cells may represent an adaptive strategy to optimize both light utilization and defense in the high-altitude environment. By extending light capture and primary photochemistry to the leaf surface, epidermal pavement cells can supplement mesophyll carbon gain during periods of low temperature and limited stomatal conductance, when mesophyll photosynthesis is constrained. However, we still lack direct evidence to prove whether epidermal pavement cells of *R. sericea* contain functional chloroplasts. Visualization is recommended in future work, such as chlorophyll autofluorescence, confocal imaging and subcellular localization.

While traditional gene expression studies often focus on individual genes, overlooking their coordinated interactions, we employed co-expression network analysis by hdWGCNA to delineate regulatory relationships (Langfelder and Horvath, 2008; Cao et al., 2023). Cell type–phenotype associations across eight defined modules revealed robust correlations between specific clusters and transcriptional programs, and cleanly onto specific biological processes. These functional hubs may reflect key transcriptional landscape that are associated with leaf developmental patterns potentially relevant to alpine adaptation in *R. sericea*. Collectively, these modules suggest coordinated biological programs rather than isolated gene effects. To link modules to ecological outcomes, targeted validation is needed in the future, such as cell-specific perturbations (RNAi/CRISPR), spatially resolved transcriptomics/metabolomics, and physiological assays.

At the gene-set scale, we further identified physiological adaptation mechanisms. Notably, enrichment of SPL7 target genes—SPL7 being a central regulator of copper deficiency responses—indicates that copper homeostasis plays a potential role in autumn leaf growth of *R. sericea* under copper-deficient soil conditions in its habitat (Zhang et al., 2002; Bing et al., 2016). Mechanistically, SPL7 enhances copper uptake and utilization by activating copper-responsive genes under deficiency, thereby maintaining micronutrient equilibrium. Our data suggest that the activation of the SPL7-mediated copper homeostasis network is associated with the adaptive response in *R. sericea*, and may represents a promising candidate mechanism. Although this study suggests a potential key role for the SPL7 module in the leaf development of *R. sericea*, its direct physiological function and causal relationship require further confirmation through future genetic experiments combined with physiological phenotype measurements.

### 4.3 Developmental trajectories provide insights into plateau plant adaptation

Analyzing leaf developmental dynamics allows us to elucidate how plants regulate morphogenetic programs to generate specialized functional structures. Such analyses not only deepen our understanding of fundamental developmental mechanisms but also reveal how plants adapt to environmental stress.

Previous pseudotime studies suggested that leaf primordia represent the main point of origin, although the timing of terminal differentiation varies among species (Bai et al., 2022; Wang et al., 2022a; Zang et al., 2023; Liang et al., 2025). Interestingly, work on peanut leaf morphogenesis revealed that epidermal cell fate can be specified even prior to primordium formation (Liu et al., 2021). In our study, the developmental trajectory began with clusters of protoblasts exhibiting strong proliferative and differentiative potential—namely, meristematic and proliferating cells—and culminated in the differentiation of mesophyll cells (**Figure 5A**). We infer that a hierarchical program in which differentiation potential is progressively restricted and metabolic specialization is sequentially acquired. The positioning of mesophyll cells at the terminal state may imply that their differentiation requires extended developmental input, particularly the coordination of chloroplast biogenesis, cell expansion, and establishment of photosynthetic machinery. This temporal delay likely ensures that mesophyll maturation is synchronized with leaf expansion, thereby optimizing carbon assimilation once the photosynthetic surface has fully developed. Together, these findings describe the diversity of developmental programs shaping leaf formation and suggest that *R. sericea* may exhibit a trajectory optimized for energy acquisition in the alpine environment, pending further validation.

### 4.4 *RO6G37307* regulates morphogenesis and functional acquisition of leaf epidermal trichomes

As key adaptive structures on leaf surfaces, epidermal trichomes serve as a valuable model system for studying plant development, with significance in both fundamental research and commercial applications (Dong et al., 2023), and are evolutionarily conserved across diverse plateau plant taxa (Wu et al., 2024a). During leaf development of *R. sericea*, epidermal trichomes contribute substantially to resistance against biotic and abiotic stresses. Their presence is associated with the species’ adaptability to high-altitude conditions, reflected by function analyses in cell fate differentiation programs. Based on the temporal regulation of development, trichome cells are closely related to epidermal pavement cells at the late stage of leaf epidermal differentiation. This discovery not only reveals cell fate transitions during epidermal development, thus expanding our understanding of leaf epidermal differentiation, but also suggests that the spatiotemporal initiation of trichome morphogenesis is closely related to environmental adaptation.

Concurrently, our analysis further identified that *RO6G37307* is a gene within the Phylloplanin family. Phylloplanins are cysteine-rich, secreted proteins first characterized in *Nicotiana tabacum* (Shepherd et al., 2005), where they mediate chemical defense on phylloplane surfaces by inhibiting airborne pathogens (Kroumova et al., 2007, 2013; Sahoo et al., 2014; Freire et al., 2017). Beyond this canonical role, our findings suggest the potential functional scope of phylloplanin-like gene by linking the tissue-specific expression of *RO6G37307* to trichome morphogenesis. It is important to note, however, that while phylogenetic, structural prediction, and subcellular localization analyses converge to support its identification as a secreted phylloplanin-like protein, they do not constitute definitive proof. A conclusive functional assignment will therefore require targeted biochemical or genetic validation.

**Figure 7:**
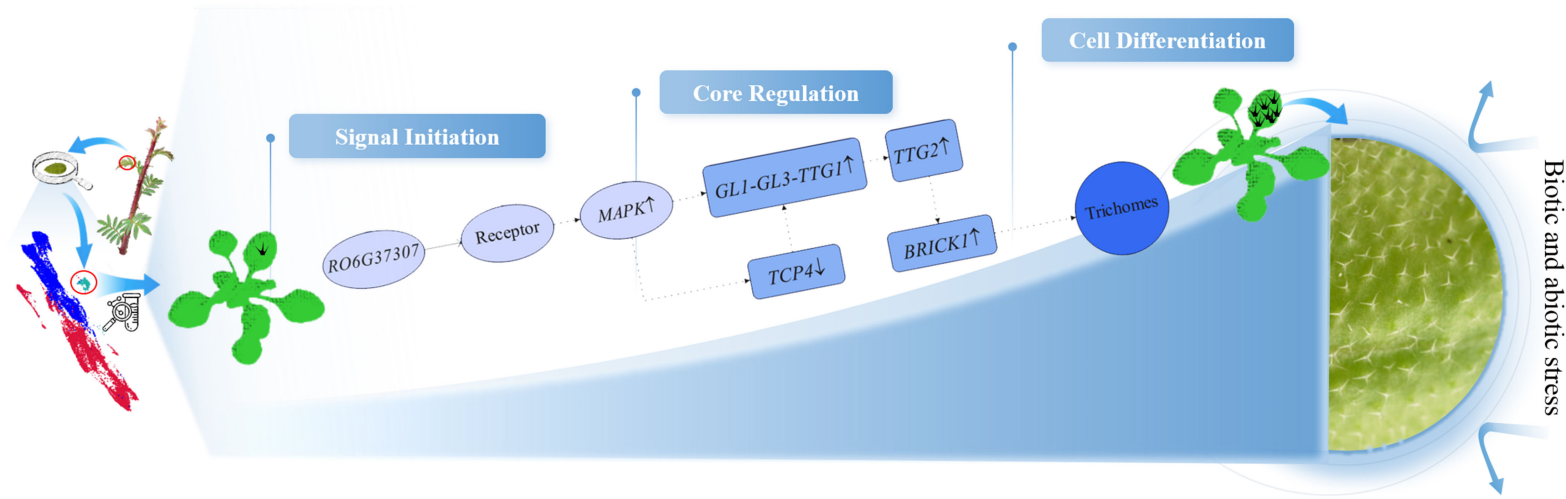
A proposed mechanism for *R. sericea* leaf trichome differentiation. RO6G37307-mediated regulation of epidermal trichome development and functional acquisition (↑/↓: up/down-regulation of expression).

In addition, *TCP4* and *TTG2*, known as trichome-specific markers (p-value = 4.4e-29, p-value = 3.6e-19), exhibit unique regulatory timing and high expression levels in *R. sericea* trichome clusters (**Supplementary Figure S8**-**S11**). Previous studies have shown that *TCP4* acts as a negative regulator of trichome development: *tcp4* mutants display increased trichome density and four-branched morphology (Vadde et al., 2018; Wang et al., 2022c). *TTG2* expression in late-stage trichome morphogenesis is co-regulated by the TTG1-GL1-GL3 complex and (mitogen-activated protein kinase) MAPK signaling (Pattanaik et al., 2014; Ma et al., 2024). Upon induction, *TTG2* directly activates cytoskeleton-associated genes (e.g., *BRICK1*), remodeling microtubule and actin networks to promote morphogenesis (Liu et al., 2024). These established roles align with the trichome phenotypes observed upon ectopic overexpression of *RO6G37307*. Integrative analysis leads us to propose one speculative model wherein *RO6G37307* may be associated with leaf epidermal trichome development via interactions with the *TTG2* and *TCP4* regulatory networks (**Figure 7**). In this model, *RO6G37307* might affect extracellular ROS homeostasis, potentially influencing the MAPK cascade to modulate *TTG2* expression and *TCP4* activity (Xu et al., 2013; Taj et al., 2014; Rayapuram et al., 2018), thereby creating a permissive environment for trichome morphogenesis (Ma et al., 2024). However, this proposed pathway is inferred from transcriptomic correlations and published literature rather than demonstrated causal relationships. Accordingly, further experimental evidence will be required to test and refine this hypothetical regulatory framework. At present, our findings suggest a potential connection between *RO6G37307* expression, leaf epidermal trichome cell differentiation, and stress-related signaling processes, which may contribute to adaptive traits in *R. sericea* under alpine environments, but such adaptive significance remains to be directly demonstrated.

## 5 Conclusion

This study used single-cell transcriptomics to construct the cell atlas and characterize the temporal program of autumn leaves development in *R. sericea*. Our analyses highlight several features that may be relevant to alpine adaptation, including presence of transcriptionally active nuclear-encoded genes involved in chloroplast function in epidermal pavement cells, transcriptomic signatures consistent with *SPL7*-linked copper homeostasis, and a putative *RO6G37307–TTG2–TCP4* regulatory module associated with trichome development. These strategies could collectively contribute to the plant’s resilience to plateau stress. Overall, our findings offer insights into potential molecular mechanisms, involving developmental and physiological adjustments, that may contribute to the adaptation of *R. sericea* to high-altitude environments. These observations advance our understanding of alpine plant adaptation and establish a foundation for future research into adaptive strategies in other species, with potential implications for efforts to improve plant resilience under climate change.

## Data Availability

The datasets presented in this study can be found in online repositories. The names of the repository/repositories and accession number(s) can be found in the article/**Supplementary Material**.

## References

[B1] Amini S. Doyle J. J. Libault M. (2023). The evolving definition of plant cell type. Front. Plant Sci. 14. doi: 10.3389/fpls.2023.1271070, PMID: 37692436 PMC10485272

[B2] Antão L. H. Weigel B. Strona G. Hällfors M. Kaarlejärvi E. Dallas T. . (2022). Climate change reshuffles northern species within their niches. Nat. Climate Change 12, 587–592. doi: 10.1038/s41558-022-01381-x

[B3] Bai Y. Liu H. Lyu H. Su L. Xiong J. Cheng Z. M. (2022). Development of a single-cell atlas for woodland strawberry (*Fragaria vesca*) leaves during early Botrytis cinerea infection using single cell RNA-seq. Hortic. Res. 9, uhab055. doi: 10.1093/hr/uhab055, PMID: 35043166 PMC8969069

[B4] Barton K. A. Schattat M. H. Jakob T. Hause G. Wilhelm C. Mckenna J. F. . (2016). Epidermal pavement cells of Arabidopsis have chloroplasts. Plant Physiol. 171, 723–726. doi: 10.1104/pp.16.00608, PMID: 27288524 PMC4902630

[B5] Becht E. McInnes L. Healy J. Dutertre C. A. Kwok I. W. H. Ng L. G. . (2018). Dimensionality reduction for visualizing single-cell data using UMAP. Nat. Biotechnol. 37, 38–44. doi: 10.1038/nbt.4314, PMID: 30531897

[B6] Bing H. Wu Y. Zhou J. Li R. Luo J. Yu D. (2016). Vegetation and cold trapping modulating elevation-dependent distribution of trace metals in soils of a high mountain in eastern Tibetan Plateau. Sci. Rep. 6, 24081. doi: 10.1038/srep24081, PMID: 27052807 PMC4823730

[B7] Cabon A. Kannenberg S. A. Arain A. Babst F. Baldocchi D. Belmecheri S. . (2022). Cross-biome synthesis of source versus sink limits to tree growth. Science 376, 758–761. doi: 10.1126/science.abm4875, PMID: 35549405

[B8] Camacho C. Coulouris G. Avagyan V. Ma N. Papadopoulos J. Bealer K. . (2009). BLAST+: architecture and applications. BMC Bioinf. 10, 421. doi: 10.1186/1471-2105-10-421, PMID: 20003500 PMC2803857

[B9] Cao Y. Ma J. Han S. Hou M. Wei X. Zhang X. . (2023). Single-cell RNA sequencing profiles reveal cell type-specific transcriptional regulation networks conditioning fungal invasion in maize roots. Plant Biotechnol. J. 21, 1839–1859. doi: 10.1111/pbi.14097, PMID: 37349934 PMC10440994

[B10] Chen H. Yin X. Guo L. Yao J. Ding Y. Xu X. . (2021). PlantscRNAdb: A database for plant single-cell RNA analysis. Mol. Plant 14, 855–857. doi: 10.1016/j.molp.2021.05.002, PMID: 33962062

[B11] Conde D. Triozzi P. M. Balmant K. M. Doty A. L. Miranda M. Boullosa A. . (2021). A robust method of nuclei isolation for single-cell RNA sequencing of solid tissues from the plant genus *Populus*. PLoS One 16, e0251149. doi: 10.1371/journal.pone.0251149, PMID: 33974645 PMC8112699

[B12] Conte M. I. Fuentes-Trillo A. Domínguez Conde C. (2024). Opportunities and tradeoffs in single-cell transcriptomic technologies. Trends Genet. 40, 83–93. doi: 10.1016/j.tig.2023.10.003, PMID: 37953195

[B13] Dobin A. Davis C. A. Schlesinger F. Drenkow J. Zaleski C. Jha S. . (2013). STAR: ultrafast universal RNA-seq aligner. Bioinformatics 29, 15–21. doi: 10.1093/bioinformatics/bts635, PMID: 23104886 PMC3530905

[B14] Dong Y. Li S. Wu H. Gao Y. Feng Z. Zhao X. . (2023). Advances in understanding epigenetic regulation of plant trichome development: a comprehensive review. Horticulture Res. 10, uhad145. doi: 10.1093/hr/uhad145, PMID: 37691965 PMC10483894

[B15] Dow C. Kim A. Y. D’Orangeville L. Gonzalez-Akre E. B. Helcoski R. Herrmann V. . (2022). Warm springs alter timing but not total growth of temperate deciduous trees. Nature 608, 552–557. doi: 10.1038/s41586-022-05092-3, PMID: 35948636

[B16] Freire L. Santana J. O. Oliveira de Sousa A. Bispo dos Santos J. Barbosa de Oliveira I. Alvim F. C. . (2017). Tc PHYLL, a cacao phylloplanin expressed in young tissues and glandular trichomes. Physiol. Mol. Plant Pathol. 100, 126–135. doi: 10.1016/j.pmpp.2017.06.002

[B17] Fu Y. H. Zhao H. Piao S. Peaucelle M. Peng S. Zhou G. . (2015). Declining global warming effects on the phenology of spring leaf unfolding. Nature 526, 104–107. doi: 10.1038/nature15402, PMID: 26416746

[B18] Gao S. Liang E. Liu R. Babst F. Camarero J. J. Fu Y. H. . (2022). An earlier start of the thermal growing season enhances tree growth in cold humid areas but not in dry areas. Nat. Ecol. Evol. 6, 397–404. doi: 10.1038/s41559-022-01668-4, PMID: 35228669

[B19] Gao Y. D. Zhang Y. Gao X. F. Zhu Z. M. (2015). Pleistocene glaciations, demographic expansion and subsequent isolation promoted morphological heterogeneity: A phylogeographic study of the alpine Rosa sericea complex (Rosaceae). Sci. Rep. 5, 11698. doi: 10.1038/srep11698, PMID: 26123942 PMC5155592

[B20] Gene Ontology, C (2008). The gene ontology project in 2008. Nucleic Acids Res. 36, D440–D444. doi: 10.1093/nar/gkm883, PMID: 17984083 PMC2238979

[B21] Guo X. Wang Y. Zhao C. Tan C. Yan W. Xiang S. . (2025). An *Arabidopsis* single-nucleus atlas decodes leaf senescence and nutrient allocation. Cell. 188, 2856–2871. doi: 10.1016/j.cell.2025.03.024, PMID: 40220755

[B22] Hallgren J. Tsirigos K. D. Pedersen M. D. Almagro Armenteros J. J. Marcatili P. Nielsen H. . (2022). DeepTMHMM predicts alpha and beta transmembrane proteins using deep neural networks. bioRxiv. doi: 10.1101/2022.04.08.487609

[B23] He Z. Luo Y. Zhou X. Zhu T. Lan Y. Chen D. (2024). scPlantDB: a comprehensive database for exploring cell types and markers of plant cell atlases. Nucleic Acids Res. 52, D1629–D1638. doi: 10.1093/nar/gkad706, PMID: 37638765 PMC10767885

[B24] Irieda H. (2022). Emerging roles of motile epidermal chloroplasts in plant immunity. Int. J. Mol. Sci. 23, 4043. doi: 10.3390/ijms23074043, PMID: 35409402 PMC8999904

[B25] Irieda H. Takano Y. (2021). Epidermal chloroplasts are defense-related motile organelles equipped with plant immune components. Nat. Commun. 12, 2739. doi: 10.1038/s41467-021-22977-5, PMID: 34016974 PMC8137707

[B26] Jiao R. Wu B. Liang Z. Gao P. Gao X. (2023). GLV reveal species differences and responses to environment in alpine shrub *Rosa sericea* complex. Sci. Total Environ. 896, 166146. doi: 10.1016/j.scitotenv.2023.166146, PMID: 37595914

[B27] Jin J. J. Lu P. Xu Y. L. Tao J. M. Li Z. F. Wang S. B. . (2022). PCMDB: a curated and comprehensive resource of plant cell markers. Nucleic Acids Res. 50, D1448–D1455. doi: 10.1093/nar/gkab949, PMID: 34718712 PMC8728192

[B28] Jin Y. Liao M. Hou Y. Wang H. Xia H. Xia J. . (2024). Spatial patterns and variations in leaf traits of alpine plants on the interior Tibetan Plateau. Global Ecol. Conserv. 53, e03037. doi: 10.1016/j.gecco.2024.e03037

[B29] Johnson C. S. Kolevski B. Smyth D. R. (2002). TRANSPARENT TESTA GLABRA2, a trichome and seed coat development gene of *Arabidopsis*, encodes a WRKY transcription factor. Plant Cell 14, 1359–1375. doi: 10.1105/tpc.001404, PMID: 12084832 PMC150785

[B30] Jovic D. Liang X. Zeng H. Lin L. Xu F. Luo Y. (2022). Single-cell RNA sequencing technologies and applications: A brief overview. Clin. Transl. Med. 12, e694. doi: 10.1002/ctm2.694, PMID: 35352511 PMC8964935

[B31] Kim J. Y. Symeonidi E. Pang T. Y. Denyer T. Weidauer D. Bezrutczyk M. . (2021). Distinct identities of leaf phloem cells revealed by single cell transcriptomics. Plant Cell 33, 511–530. doi: 10.1093/plcell/koaa060, PMID: 33955487 PMC8136902

[B32] Kobak D. Berens P. (2019). The art of using t-SNE for single-cell transcriptomics. Nat. Commun. 10, 5416. doi: 10.1038/s41467-019-13056-x, PMID: 31780648 PMC6882829

[B33] Kroumova A. B. Sahoo D. K. Raha S. Goodin M. Maiti I. B. Wagner G. J. (2013). Expression of an apoplast-directed, T-phylloplanin-GFP fusion gene confers resistance against *Peronospora tabacina* disease in a susceptible tobacco. Plant Cell Rep. 32, 1771–1782. doi: 10.1007/s00299-013-1490-6, PMID: 23942845

[B34] Kroumova A. B. Shepherd R. W. Wagner G. J. (2007). Impacts of T-Phylloplanin gene knockdown and of Helianthus and Datura phylloplanins on *Peronospora tabacina* spore germination and disease potential. Plant Physiol. 144, 1843–1851. doi: 10.1104/pp.107.097584, PMID: 17573541 PMC1949898

[B35] Kumar S. Chatterjee U. David Raj A. Sooryamol K. (2024a). “ Global warming and climate crisis/extreme events,” in Climate crisis: Adaptive approaches and sustainability (Switzerland: Springer), 3–18. doi: 10.1007/978-3-031-44397-8_1

[B36] Kumar S. Stecher G. Suleski M. Sanderford M. Sharma S. Tamura K. (2024b). MEGA12: molecular evolutionary genetic analysis version 12 for adaptive and green computing. Mol. Biol. Evol. 41, msae263. doi: 10.1093/molbev/msae263, PMID: 39708372 PMC11683415

[B37] Langfelder P. Horvath S. (2008). WGCNA: an R package for weighted correlation network analysis. BMC Bioinf. 9, 559. doi: 10.1186/1471-2105-9-559, PMID: 19114008 PMC2631488

[B38] Lee T. A. Illouz-Eliaz N. Nobori T. Xu J. Jow B. Nery J. R. . (2025). A single-cell, spatial transcriptomic atlas of the *Arabidopsis* life cycle. Nat. Plants. 11, 1960–1975. doi: 10.1038/s41477-025-02072-z, PMID: 40830271 PMC12416547

[B39] Li C. Wood J. C. Vu A. H. Hamilton J. P. Rodriguez Lopez C. E. Payne R. M. E. . (2023). Single-cell multi-omics in the medicinal plant Catharanthus roseus. Nat. Chem. Biol. 19, 1031–1041. doi: 10.1038/s41589-023-01327-0, PMID: 37188960 PMC10374443

[B40] Li P. Zhu Q. Peng C. Zhang J. Wang M. Zhang J. . (2019). Change in autumn vegetation phenology and the climate controls from 1982 to 2012 on the Qinghai-Tibet Plateau. Front. Plant Sci. 10. doi: 10.3389/fpls.2019.01677, PMID: 32010162 PMC6977410

[B41] Li Y. Ma H. Wu Y. Ma Y. Yang J. Li Y. . (2024). Single-cell transcriptome atlas and regulatory dynamics in developing cotton anthers. Adv. Sci. (Weinh) 11, e2304017. doi: 10.1002/advs.202304017, PMID: 37974530 PMC10797427

[B42] Liang J.-h. Wu Z.-q. Zhang Y.-X. Yang Y.-B. Wang S.-Y. Gai M.-Y. . (2025). Single-cell RNA sequencing of shoot apex reveals the mechanism of cyclin regulating cell division via auxin signaling pathway in *Populus alba*. Front. Plant Sci. 16. doi: 10.3389/fpls.2025.1555388, PMID: 40104035 PMC11913855

[B43] Liu H. Hu D. Du P. Wang L. Liang X. Li H. . (2021). Single-cell RNA-seq describes the transcriptome landscape and identifies critical transcription factors in the leaf blade of the allotetraploid peanut (*Arachis hypogaea* L.). Plant Biotechnol. J. 19, 2261–2276. doi: 10.1111/pbi.13656, PMID: 34174007 PMC8541777

[B44] Liu L. Wang Y. Cao W. Yang L. Zhang C. Yuan L. . (2024). TRANSPARENT TESTA GLABRA2 defines trichome cell shape by modulating actin cytoskeleton in *Arabidopsis thaliana*. Plant Physiol. 195, 1256–1276. doi: 10.1093/plphys/kiae091, PMID: 38391271

[B45] Liu W. Zhang Y. Fang X. Tran S. Zhai N. Yang Z. . (2022a). Transcriptional landscapes of *de novo* root regeneration from detached *Arabidopsis* leaves revealed by time-lapse and single-cell RNA sequencing analyses. Plant Commun. 3, 100306. doi: 10.1016/j.xplc.2022.100306, PMID: 35605192 PMC9284295

[B46] Liu Z. Kong X. Long Y. Liu S. Zhang H. Jia J. . (2023). Integrated single-nucleus and spatial transcriptomics captures transitional states in soybean nodule maturation. Nat. Plants 9, 515–524. doi: 10.1038/s41477-023-01387-z, PMID: 37055554

[B47] Liu Z. Wang J. Zhou Y. Zhang Y. Qin A. Yu X. . (2022b). Identification of novel regulators required for early development of vein pattern in the cotyledons by single-cell RNA-sequencing. Plant J. 110, 7–22. doi: 10.1111/tpj.15719, PMID: 35218590 PMC9310732

[B48] Liu Z. Zhou Y. Guo J. Li J. Tian Z. Zhu Z. . (2020). Global dynamic molecular profiling of stomatal lineage cell development by single-cell RNA sequencing. Mol. Plant 13, 1178–1193. doi: 10.1016/j.molp.2020.06.010, PMID: 32592820

[B49] Lopez-Anido C. B. Vaten A. Smoot N. K. Sharma N. Guo V. Gong Y. . (2021). Single-cell resolution of lineage trajectories in the *Arabidopsis* stomatal lineage and developing leaf. Dev. Cell 56, 1043–1055.e1044. doi: 10.1016/j.devcel.2021.03.014, PMID: 33823130 PMC8054824

[B50] Ma Y. Flückiger I. Nicolet J. Pang J. Dickinson J. B. De Bellis D. . (2024). Comparisons of two receptor-MAPK pathways in a single cell-type reveal mechanisms of signalling specificity. Nat. Plants 10, 1343–1362. doi: 10.1038/s41477-024-01768-y, PMID: 39256564 PMC11410668

[B51] Ma X. Yan H. Yang J. Liu Y. Li Z. Sheng M. . (2022). PlantGSAD: a comprehensive gene set annotation database for plant species. Nucleic Acids Res. 50, D1456–D1467. doi: 10.1093/nar/gkab794, PMID: 34534340 PMC8728169

[B52] Mackenzie S. A. Mullineaux P. M. (2022). Plant environmental sensing relies on specialized plastids. J. Exp. Bot. 73, 7155–7164. doi: 10.1093/jxb/erac334, PMID: 35994779 PMC12104512

[B53] Marchant D. B. Walbot V. (2025). The establishment of the anther somatic niche with single-cell sequencing. Dev. Biol. 518, 37–47. doi: 10.1016/j.ydbio.2024.11.004, PMID: 39547468

[B54] Marques L. Hufkens K. Bigler C. Crowther T. W. Zohner C. M. Stocker B. D. (2023). Acclimation of phenology relieves leaf longevity constraints in deciduous forests. Nat. Ecol. Evol. 7, 198–204. doi: 10.1038/s41559-022-01946-1, PMID: 36635342

[B55] Martinez Del Castillo E. Zang C. S. Buras A. Hacket-Pain A. Esper J. Serrano-Notivoli R. . (2022). Climate-change-driven growth decline of European beech forests. Commun. Biol. 5, 163. doi: 10.1038/s42003-022-03107-3, PMID: 35273334 PMC8913685

[B56] Mathe C. Nick P. Pasternak T. P. (2021). Editorial: how cells build plants: regulatory mechanisms for integrated functioning of plant cells and the whole plant body. Front. Plant Sci. 12. doi: 10.3389/fpls.2021.706892, PMID: 34295348 PMC8291082

[B57] Meng F. Felton A. J. Mao J. Cong N. Smith W. K. Körner C. . (2024). Consistent time allocation fraction to vegetation green-up versus senescence across northern ecosystems despite recent climate change. Sci. Adv. 10, eadn2487. doi: 10.1126/sciadv.adn2487, PMID: 38848369 PMC11160464

[B58] Meng Y. Duan K. Shi P. Shang W. Li S. Cheng Y. . (2023). Sensitive temperature changes on the Tibetan Plateau in response to global warming. Atmospheric Res. 294, 106948. doi: 10.1016/j.atmosres.2023.106948

[B59] Mermod M. Takusagawa M. Kurata T. Kamiya T. Fujiwara T. Shikanai T. (2019). SQUAMOSA promoter-binding protein-like 7 mediates copper deficiency response in the presence of high nitrogen in Arabidopsis thaliana. Plant Cell Rep. 38, 835–846. doi: 10.1007/s00299-019-02422-0, PMID: 31093688

[B60] Mirabel A. Girardin M. P. Metsaranta J. Way D. Reich P. B. (2023). Increasing atmospheric dryness reduces boreal forest tree growth. Nat. Commun. 14, 6901. doi: 10.1038/s41467-023-42466-1, PMID: 37903759 PMC10616230

[B61] Morabito S. Miyoshi E. Michael N. Shahin S. Martini A. C. Head E. . (2021). Single-nucleus chromatin accessibility and transcriptomic characterization of Alzheimer’s disease. Nat. Genet. 53, 1143–1155. doi: 10.1038/s41588-021-00894-z, PMID: 34239132 PMC8766217

[B62] Morabito S. Reese F. Rahimzadeh N. Miyoshi E. Swarup V. (2023). hdWGCNA identifies co-expression networks in high-dimensional transcriptomics data. Cell Rep. Methods 3, 100498. doi: 10.1016/j.crmeth.2023.100498, PMID: 37426759 PMC10326379

[B63] Nakayama H. Leichty A. R. Sinha N. R. (2022). Molecular mechanisms underlying leaf development, morphological diversification, and beyond. Plant Cell 34, 2534–2548. doi: 10.1093/plcell/koac118, PMID: 35441681 PMC9252486

[B64] Pattanaik S. Patra B. Singh S. K. Yuan L. (2014). An overview of the gene regulatory network controlling trichome development in the model plant, *Arabidopsis*. Front. Plant Sci. 5. doi: 10.3389/fpls.2014.00259, PMID: 25018756 PMC4071814

[B65] Pesch M. Dartan B. Birkenbihl R. Somssich I. E. Hulskamp M. (2014). *Arabidopsis* TTG2 regulates TRY expression through enhancement of activator complex-triggered activation. Plant Cell 26, 4067–4083. doi: 10.1105/tpc.114.129379, PMID: 25304203 PMC4247571

[B66] Piao S. Liu Q. Chen A. Janssens I. A. Fu Y. Dai J. . (2019). Plant phenology and global climate change: Current progresses and challenges. Global Change Biol. 25, 1922–1940. doi: 10.1111/gcb.14619, PMID: 30884039

[B67] Procko C. Lee T. Borsuk A. Bargmann B. O. R. Dabi T. Nery J. R. . (2022). Leaf cell-specific and single-cell transcriptional profiling reveals a role for the palisade layer in UV light protection. Plant Cell 34, 3261–3279. doi: 10.1093/plcell/koac167, PMID: 35666176 PMC9421592

[B68] Pu G. Han L. Chen L. Wan D. Teng H. (2025). Elevational dynamics of vegetation changes in response to climate change on the Tibetan plateau. Sci. Rep. 15, 9813. doi: 10.1038/s41598-025-94896-0, PMID: 40119160 PMC11928499

[B69] Qin X. Tape C. J. (2024). Functional analysis of cell plasticity using single-cell technologies. Trends Cell Biol. 34, 854–864. doi: 10.1016/j.tcb.2024.01.006, PMID: 38355348

[B70] Qiu X. Mao Q. Tang Y. Wang L. Chawla R. Pliner H. A. . (2017). Reversed graph embedding resolves complex single-cell trajectories. Nat. Methods 14, 979–982. doi: 10.1038/nmeth.4402, PMID: 28825705 PMC5764547

[B71] Ram H. (2024). Adaptation strategies of plants to climate change: mechanisms and implications. Res. Rev. Int. J. Multidiscip. 9, 312–319. doi: 10.31305/rrijm.2024.v09.n03.036

[B72] Rayapuram N. Bigeard J. Alhoraibi H. Bonhomme L. Hesse A.-M. Vinh J. . (2018). Quantitative phosphoproteomic analysis reveals shared and specific targets of *Arabidopsis* mitogen-activated protein kinases (MAPKs) MPK3, MPK4, and MPK6. Mol. Cell. Proteomics 17, 61–80. doi: 10.1074/mcp.RA117.000135, PMID: 29167316 PMC5750851

[B73] Reich P. B. Bermudez R. Montgomery R. A. Rich R. L. Rice K. E. Hobbie S. E. . (2022). Even modest climate change may lead to major transitions in boreal forests. Nature 608, 540–545. doi: 10.1038/s41586-022-05076-3, PMID: 35948640

[B74] Repo T. Wu D. Hänninen H. (2021). Autumn cold acclimation of shoots does not explain the northern distribution limit of three southern exotic tree species in Finland. Environ. Exp. Bot. 188, 104526. doi: 10.1016/j.envexpbot.2021.104526

[B75] Roman A. O. Jimenez-Sandoval P. Augustin S. Broyart C. Hothorn L. A. Santiago J. (2022). HSL1 and BAM1/2 impact epidermal cell development by sensing distinct signaling peptides. Nat. Commun. 13, 876. doi: 10.1038/s41467-022-28558-4, PMID: 35169143 PMC8847575

[B76] Sahoo D. K. Raha S. Hall J. T. Maiti I. B. (2014). Overexpression of the synthetic chimeric native-T-phylloplanin-GFP genes optimized for monocot and dicot plants renders enhanced resistance to blue mold disease in tobacco (*N. tabacum* L.). Sci. World J. 2014, 1–12. doi: 10.1155/2014/601314, PMID: 24778589 PMC3980785

[B77] Saladin B. Pellissier L. Graham C. H. Nobis M. P. Salamin N. Zimmermann N. E. (2020). Rapid climate change results in long-lasting spatial homogenization of phylogenetic diversity. Nat. Commun. 11, 4663. doi: 10.1038/s41467-020-18343-6, PMID: 32938914 PMC7495423

[B78] Seth P. Sebastian J. (2024). Plants and global warming: challenges and strategies for a warming world. Plant Cell Rep. 43, 27. doi: 10.1007/s00299-023-03083-w, PMID: 38163826

[B79] Shannon P. Markiel A. Ozier O. Baliga N. S. Wang J. T. Ramage D. . (2003). Cytoscape: a software environment for integrated models of biomolecular interaction networks. Genome Res. 13, 2498–2504. doi: 10.1101/gr.1239303, PMID: 14597658 PMC403769

[B80] Shen M. Wang S. Jiang N. Sun J. Cao R. Ling X. . (2022). Plant phenology changes and drivers on the Qinghai–Tibetan Plateau. Nat. Rev. Earth Environ. 3, 633–651. doi: 10.1038/s43017-022-00317-5

[B81] Shepherd R. W. Bass W. T. Houtz R. L. Wagner G. J. (2005). Phylloplanins of tobacco are defensive proteins deployed on aerial surfaces by short glandular trichomes. Plant Cell 17, 1851–1861. doi: 10.1105/tpc.105.031559, PMID: 15894716 PMC1143082

[B82] Sigdel S. R. Zheng X. Babst F. Camarero J. J. Gao S. Li X. . (2024). Accelerated succession in Himalayan alpine treelines under climatic warming. Nat. Plants 10, 1909–1918. doi: 10.1038/s41477-024-01855-0, PMID: 39558135

[B83] Stuart T. Butler A. Hoffman P. Hafemeister C. Papalexi E. Mauck W. M. . (2019). Comprehensive integration of single-cell data. Cell 177, 1888–1902 e1821. doi: 10.1016/j.cell.2019.05.031, PMID: 31178118 PMC6687398

[B84] Subramanian A. Tamayo P. Mootha V. K. Mukherjee S. Ebert B. L. Gillette M. A. . (2005). Gene set enrichment analysis: a knowledge-based approach for interpreting genome-wide expression profiles. Proc. Natl. Acad. Sci. 102, 15545–15550. doi: 10.1073/pnas.0506580102, PMID: 16199517 PMC1239896

[B85] Sun P. Hao R. Fan F. Wang Y. Zhu F. (2025). Adaptation of high-altitude plants to plateau abiotic stresses: A case study of the Qinghai-Tibet Plateau. Int. J. Mol. Sci. 26, 2292. doi: 10.3390/ijms26052292, PMID: 40076909 PMC11900590

[B86] Sun Q. Zhu J. Li B. Zhu S. Huang J. Chen X. . (2024). Drier August and colder September slow down the delaying trend of leaf senescence in herbaceous plants on the Qinghai-Tibetan Plateau. Sci. Total Environ. 908, 168504. doi: 10.1016/j.scitotenv.2023.168504, PMID: 37952658

[B87] Taj G. Agarwal P. Grant M. Kumar A. (2014). MAPK machinery in plants. Plant Signaling Behav. 5, 1370–1378. doi: 10.4161/psb.5.11.13020, PMID: 20980831 PMC3115236

[B88] Tang S. Vlug A. Piao S. Li F. Wang T. Krinner G. . (2023). Regional and tele-connected impacts of the Tibetan Plateau surface darkening. Nat. Commun. 14, 32. doi: 10.1038/s41467-022-35672-w, PMID: 36596797 PMC9810690

[B89] Tenorio Berrio R. Verstaen K. Vandamme N. Pevernagie J. Achon I. Van Duyse J. . (2022). Single-cell transcriptomics sheds light on the identity and metabolism of developing leaf cells. Plant Physiol. 188, 898–918. doi: 10.1093/plphys/kiab489, PMID: 34687312 PMC8825278

[B90] Teufel F. Almagro Armenteros J. J. Johansen A. R. Gislason M. H. Pihl S. I. Tsirigos K. D. . (2022). SignalP 6.0 predicts all five types of signal peptides using protein language models. Nat. Biotechnol. 40, 1023–1025. doi: 10.1038/s41587-021-01156-3, PMID: 34980915 PMC9287161

[B91] Thibivilliers S. Libault M. (2021). Enhancing our understanding of plant cell-to-cell interactions using single-cell omics. Front. Plant Sci. 12. doi: 10.3389/fpls.2021.696811, PMID: 34421948 PMC8375048

[B92] Ullah F. Gao Y. Sari İ. Jiao R.-F. Saqib S. Gao X.-F. (2022b). Macro-morphological and ecological variation in *Rosa sericea* complex. Agronomy 12, 1078. doi: 10.3390/agronomy12051078

[B93] Ullah F. Gao Y.-D. Zaman W. Gao X.-F. (2022a). Pollen morphology of *Rosa sericea* complex and their taxonomic contribution. Diversity 14, 705. doi: 10.3390/d14090705

[B94] Vadde B. V. L. Challa K. R. Nath U. (2018). The TCP4 transcription factor regulates trichome cell differentiation by directly activating GLABROUS INFLORESCENCE STEMS in *Arabidopsis thaliana*. Plant J. 93, 259–269. doi: 10.1111/tpj.13772, PMID: 29165850

[B95] Vadde B. V. L. Challa K. R. Sunkara P. Hegde A. S. Nath U. (2019). The TCP4 transcription factor directly activates TRICHOMELESS1 and 2 and suppresses trichome initiation. Plant Physiol. 181, 1587–1599. doi: 10.1104/pp.19.00197, PMID: 31575625 PMC6878003

[B96] Viterbo A. Ramot O. Chernin L. Chet I. (2002). Significance of lytic enzymes from Trichoderma spp. in the biocontrol of fungal plant pathogens. Antonie Van Leeuwenhoek 81, 549–556. doi: 10.1023/A:1020553421740, PMID: 12448750

[B97] Wang Q. Wu Y. Peng A. Cui J. Zhao M. Pan Y. . (2022a). Single-cell transcriptome atlas reveals developmental trajectories and a novel metabolic pathway of catechin esters in tea leaves. Plant Biotechnol. J. 20, 2089–2106. doi: 10.1111/pbi.13891, PMID: 35810348 PMC9616531

[B98] Wang S. Sun S. T. Zhang X. Y. Ding H. R. Yuan Y. He J. J. . (2023). The evolution of single-cell RNA sequencing technology and application: progress and perspectives. Int. J. Mol. Sci. 24, 2943. doi: 10.3390/ijms24032943, PMID: 36769267 PMC9918030

[B99] Wang X. Wang T. Xu J. Shen Z. Yang Y. Chen A. . (2022b). Enhanced habitat loss of the Himalayan endemic flora driven by warming-forced upslope tree expansion. Nat. Ecol. Evol. 6, 890–899. doi: 10.1038/s41559-022-01774-3, PMID: 35654898

[B100] Wang Y. Zhou Q. Meng Z. Abid M. A. Wang Y. Wei Y. . (2022c). Multi-dimensional molecular regulation of trichome development in *Arabidopsis* and cotton. Front. Plant Sci. 13. doi: 10.3389/fpls.2022.892381, PMID: 35463426 PMC9021843

[B101] Wang Z. Yang Z. Li F. (2019). Updates on molecular mechanisms in the development of branched trichome in *Arabidopsis* and nonbranched in cotton. Plant Biotechnol. J. 17, 1706–1722. doi: 10.1111/pbi.13167, PMID: 31111642 PMC6686129

[B102] Weih M. (2009). Genetic and environmental variation in spring and autumn phenology of biomass willows (Salix spp.): effects on shoot growth and nitrogen economy. Tree Physiol. 29, 1479–1490. doi: 10.1093/treephys/tpp081, PMID: 19793729

[B103] Wei Z. Cheng Y. Zhou C. Li D. Gao X. Zhang S. . (2019). Genome-wide identification of direct targets of the TTG1-bHLH-MYB complex in regulating trichome formation and flavonoid accumulation in *Arabidopsis thaliana*. Int. J. Mol. Sci. 20, 5014. doi: 10.3390/ijms20205014, PMID: 31658678 PMC6829465

[B104] Wolock S. L. Lopez R. Klein A. M. (2019). Scrublet: computational identification of cell doublets in single-cell transcriptomic data. Cell Syst. 8, 281–291.e289. doi: 10.1016/j.cels.2018.11.005, PMID: 30954476 PMC6625319

[B105] Wright I. J. Dong N. Maire V. Prentice I. C. Westoby M. Díaz S. . (2017). Global climatic drivers of leaf size. Science 357, 917–921. doi: 10.1126/science.aal4760, PMID: 28860384

[B106] Wu R. Cun S. Gao Y.-Q. Ma R. Zhang L. Lev-Yadun S. . (2024a). Distribution patterns of glandular trichomes in the flora of the Hengduan Mountains, southwestern China. Botanical J. Linn. Soc. 207, 83–94. doi: 10.1093/botlinnean/boae035

[B107] Wu S. Morotti A. L. M. Yang J. Wang E. Tatsis E. C. (2024b). Single-cell RNA sequencing facilitates the elucidation of the complete biosynthesis of the antidepressant hyperforin in St. John’s wort. Mol. Plant 17, 1439–1457. doi: 10.1016/j.molp.2024.08.003, PMID: 39135343

[B108] Wu C. Peng J. Ciais P. Peñuelas J. Wang H. Beguería S. . (2022). Increased drought effects on the phenology of autumn leaf senescence. Nat. Climate Change 12, 943–949. doi: 10.1038/s41558-022-01464-9

[B109] Xia K. Sun H. X. Li J. Li J. Zhao Y. Chen L. . (2022). The single-cell stereo-seq reveals region-specific cell subtypes and transcriptome profiling in Arabidopsis leaves. Dev. Cell 57, 1299–1310 e1294. doi: 10.1016/j.devcel.2022.04.011, PMID: 35512702

[B110] Xu Y. W. Cun S. Ma Z. L. He R. Sun H. Song B. (2025). Elevational variation in trichomes of the alpine subnival woolly plant Eriophyton wallichii: abiotic and biotic correlates and impacts on other traits. Ann. Bot. 136, 167–177. doi: 10.1093/aob/mcaf071, PMID: 40245118 PMC12401881

[B111] Xu J. Xie J. Yan C. Zou X. Ren D. Zhang S. (2013). A chemical genetic approach demonstrates that MPK3/MPK6 activation and NADPH oxidase-mediated oxidative burst are two independent signaling events in plant immunity. Plant J. 77, 222–234. doi: 10.1111/tpj.12382, PMID: 24245741 PMC4017028

[B112] Xu m.x. Du q.W. Tian c.h. Wang y. Jiao l.y. (2021). Stochastic gene expression drives mesophyll protoplast regeneration. Sci. Adv. 7, eabg8466. doi: 10.1126/sciadv.abg8466, PMID: 34380624 PMC8357238

[B113] Yan J. Chia J. C. Sheng H. Jung H. I. Zavodna T. O. Zhang L. . (2017). Arabidopsis pollen fertility requires the transcription factors CITF1 and SPL7 that regulate copper delivery to anthers and jasmonic acid synthesis. Plant Cell 29, 3012–3029. doi: 10.1105/tpc.17.00363, PMID: 29114014 PMC5757271

[B114] Ye F. Wang J. Li J. Mei Y. Guo G. (2023). Mapping cell atlases at the single-cell level. Advanced Sci. 11, 2305449. doi: 10.1002/advs.202305449, PMID: 38145338 PMC10885669

[B115] Ye K. Bu F. Zhong L. Dong Z. Ma Z. Tang Z. . (2024). Mapping the molecular landscape of Lotus japonicus nodule organogenesis through spatiotemporal transcriptomics. Nat. Commun. 15, 6387. doi: 10.1038/s41467-024-50737-8, PMID: 39080318 PMC11289483

[B116] Yu B. Chao D. Y. Zhao Y. (2024). How plants sense and respond to osmotic stress. J. Integr. Plant Biol. 66, 394–423. doi: 10.1111/jipb.13622, PMID: 38329193

[B117] Yu G. Wang L. G. Han Y. He Q. Y. (2012). clusterProfiler: an R package for comparing biological themes among gene clusters. OMICS 16, 284–287. doi: 10.1089/omi.2011.0118, PMID: 22455463 PMC3339379

[B118] Yuan W. Zheng Y. Piao S. Ciais P. Lombardozzi D. Wang Y. . (2019). Increased atmospheric vapor pressure deficit reduces global vegetation growth. Sci. Adv. 5, eaax1396. doi: 10.1126/sciadv.aax1396, PMID: 31453338 PMC6693914

[B119] Zhang T. Q. Chen Y. Wang J. W. (2021). A single-cell analysis of the *Arabidopsis* vegetative shoot apex. Dev. Cell 56, 1056–1074 e1058. doi: 10.1016/j.devcel.2021.02.021, PMID: 33725481

[B120] Zhang X. P. Yan X. Y. Yang X. M. (2002). Copper content and its distribution in soils of Tibet. J. Geographical Sci. 12, 343–347. doi: 10.1007/BF02837555

[B121] Zang Y. Pei Y. Cong X. Ran F. Liu L. Wang C. . (2023). Single-cell RNA-sequencing profiles reveal the developmental landscape of the *Manihot esculenta* Crantz leaves. Plant Physiol. 194, 456–474. doi: 10.1093/plphys/kiad500, PMID: 37706525 PMC10756766

[B122] Zhang S. Zhu C. Zhang X. Liu M. Xue X. Lai C. . (2023). Single-cell RNA sequencing analysis of the embryogenic callus clarifies the spatiotemporal developmental trajectories of the early somatic embryo in *Dimocarpus longan*. Plant J. 115, 1277–1297. doi: 10.1111/tpj.16319, PMID: 37235696

[B123] Zheng G. X. Terry J. M. Belgrader P. Ryvkin P. Bent Z. W. Wilson R. . (2017). Massively parallel digital transcriptional profiling of single cells. Nat. Commun. 8, 14049. doi: 10.1038/ncomms14049, PMID: 28091601 PMC5241818

[B124] Zhu M. Hsu C. W. Peralta Ogorek L. L. Taylor I. W. La Cavera S. Oliveira D. M. . (2025). Single-cell transcriptomics reveal how root tissues adapt to soil stress. Nature. 642, 721–729. doi: 10.1038/s41586-025-08941-z, PMID: 40307555 PMC12176638

[B125] Zhu M. Taylor I. W. Benfey P. N. (2022). Single-cell genomics revolutionizes plant development studies across scales. Development 149, dev200179. doi: 10.1242/dev.200179, PMID: 35285482 PMC8977093

[B126] Zlobin I. E. Kartashov A. V. Ivanov Y. V. Ivanova A. I. Pashkovskiy P. P. Gorshkova E. N. . (2024). Auxins differentially affect growth in Scots pine and Norway spruce in spring and autumn. Environ. Exp. Bot. 225, 105848. doi: 10.1016/j.envexpbot.2024.105848

[B127] Zohner C. M. Renner S. S. Sebald V. Crowther T. W. (2021). How changes in spring and autumn phenology translate into growth-experimental evidence of asymmetric effects. J. Ecol. 109, 2717–2728. doi: 10.1111/1365-2745.13682

