# Supplementary material for: Single-cell transcriptomics reveals cellular and genetic mechanisms of alpine adaptation in Rosa sericea
Source: Front Plant Sci. 2026 Feb 19;17:1733247. doi: 10.3389/fpls.2026.1733247 (PMC12960625; doi:10.3389/fpls.2026.1733247)
Supplement: Supplementary file 1 [file DataSheet1.pdf]

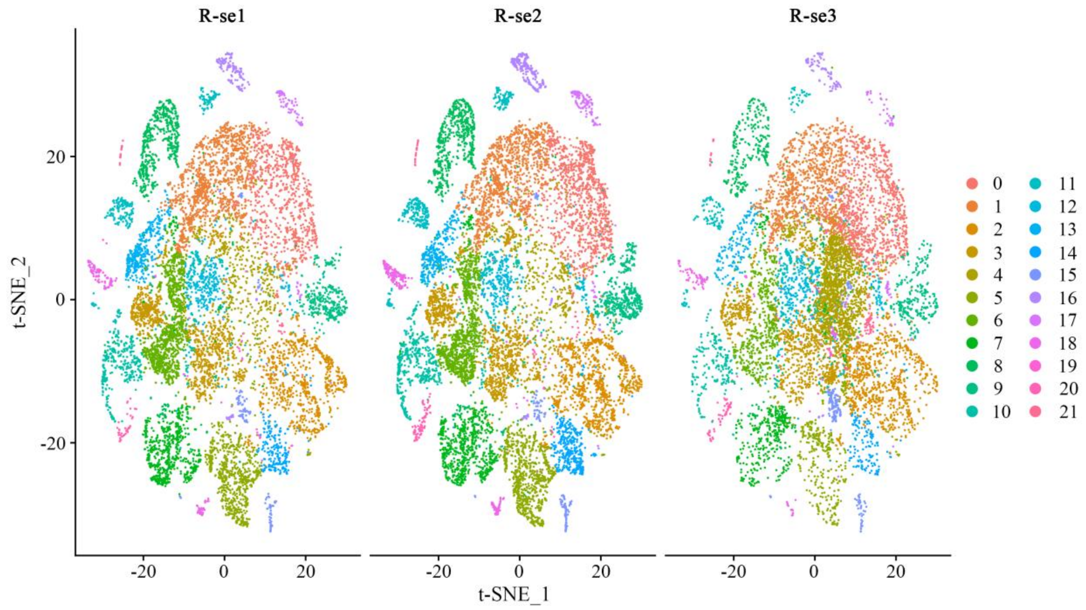

Figure S1 The t-SNE plot illustrated the clustering of *Rosa sericea* leaf cells, where each dot corresponded to an individual cell. The 22 distinct colors represented 22 specific cell clusters. The designations R-se1, R-se2, and R-se3 refered to the three biological replicates, respectively.

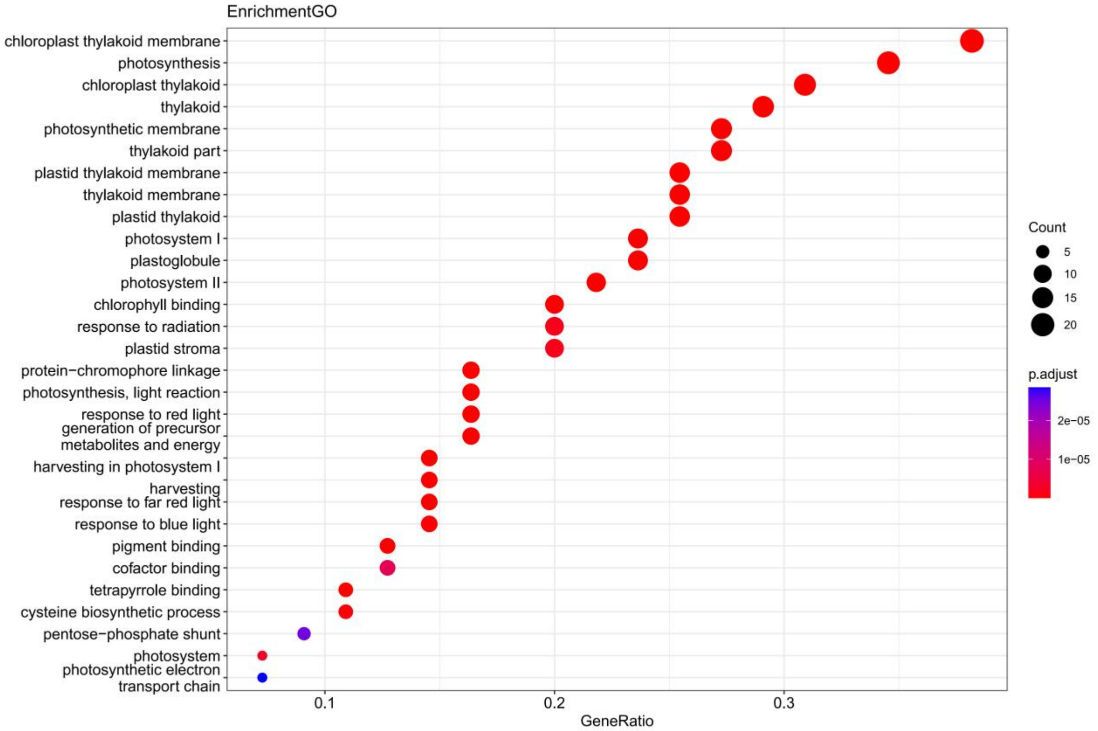

Figure S2-1 The GO enrichment dotpot of cell cluster 0 of *R. sericea*

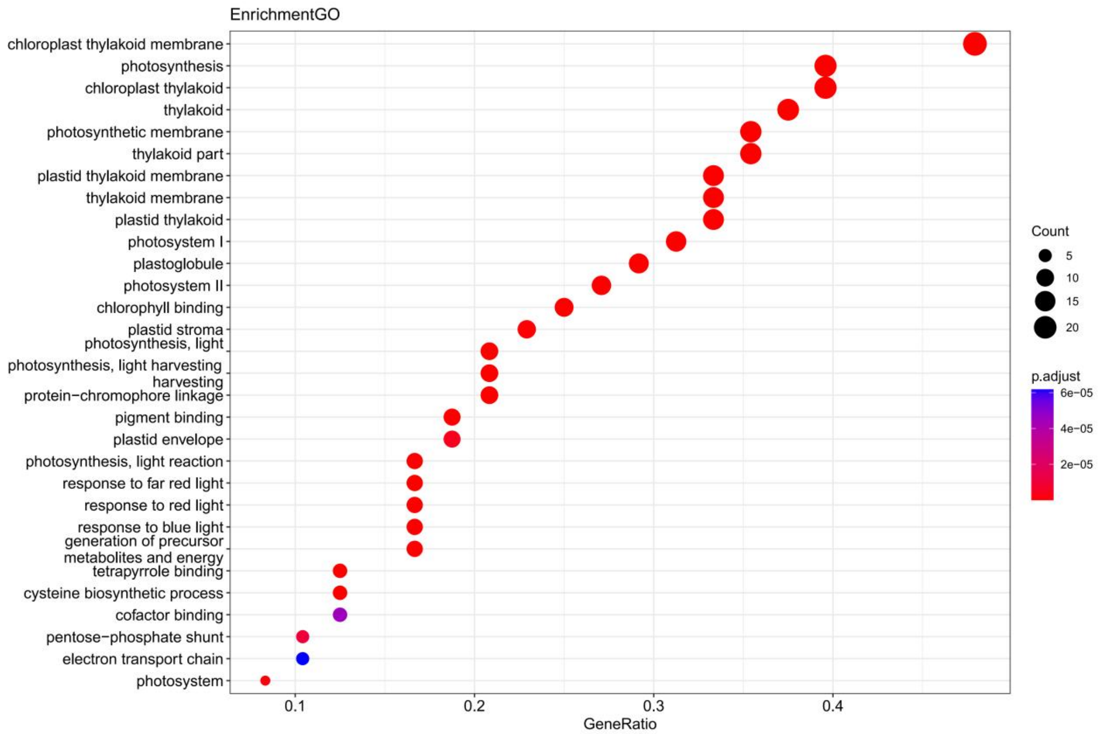

Figure S2-2 The GO enrichment dotpot of cell cluster 1 of *R. sericea*

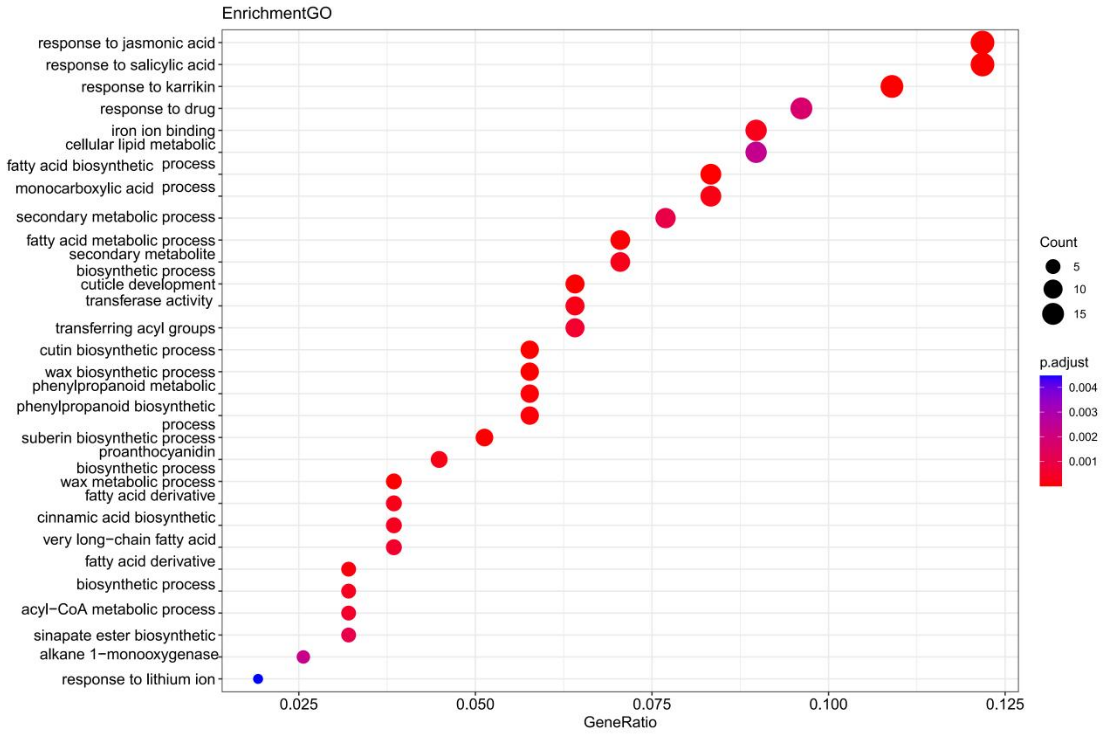

Figure S2-3 The GO enrichment dotpot of cell cluster 2 of *R. sericea*

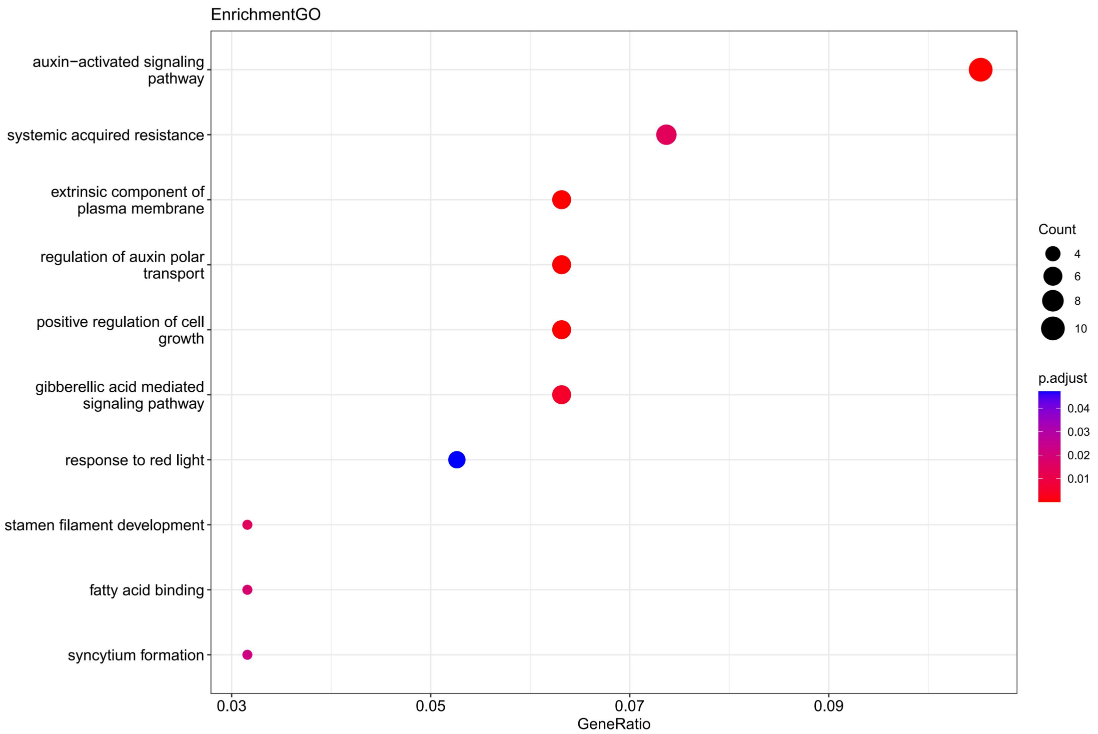

Figure S2-4 The GO enrichment dotpot of cell cluster 3 of *R. sericea*

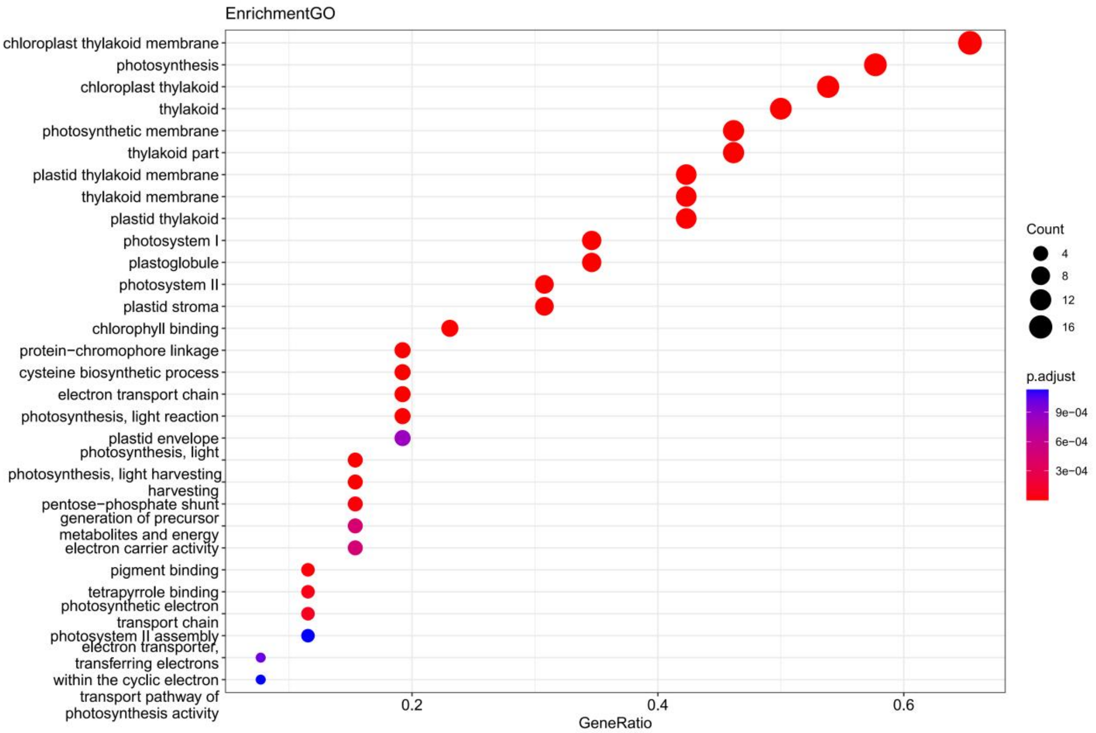

Figure S2-5 The GO enrichment dotpot of cell cluster 4 of *R. sericea*

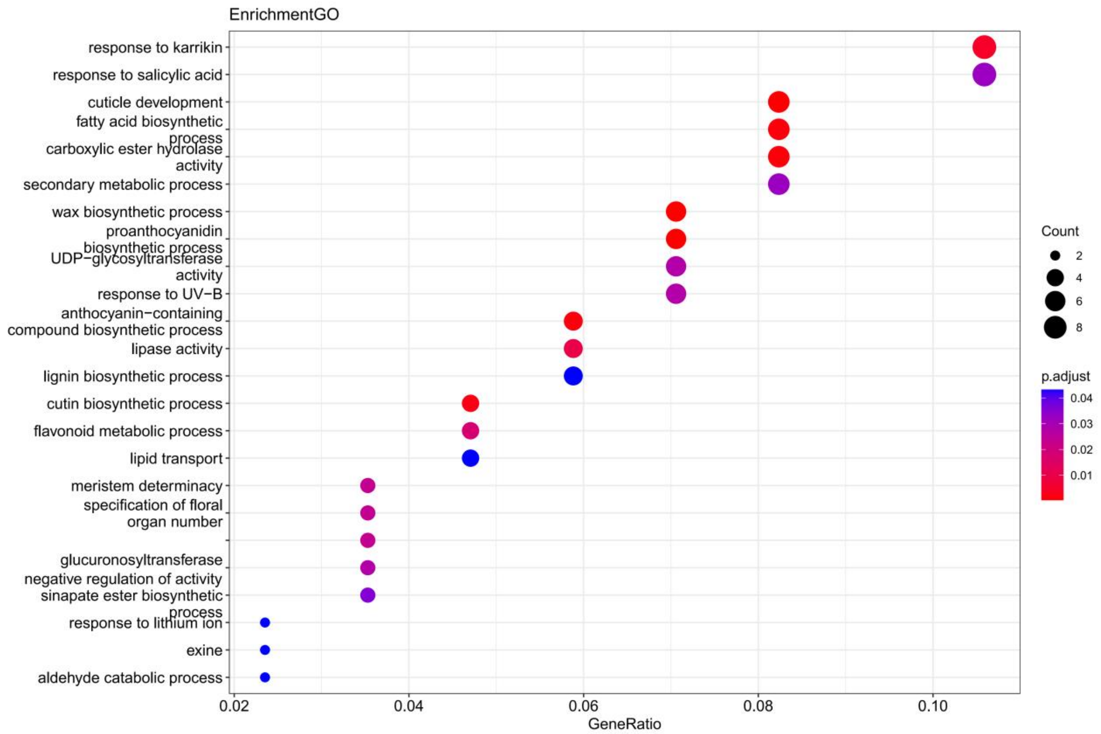

Figure S2-6 The GO enrichment dotpot of cell cluster 5 of *R. sericea*

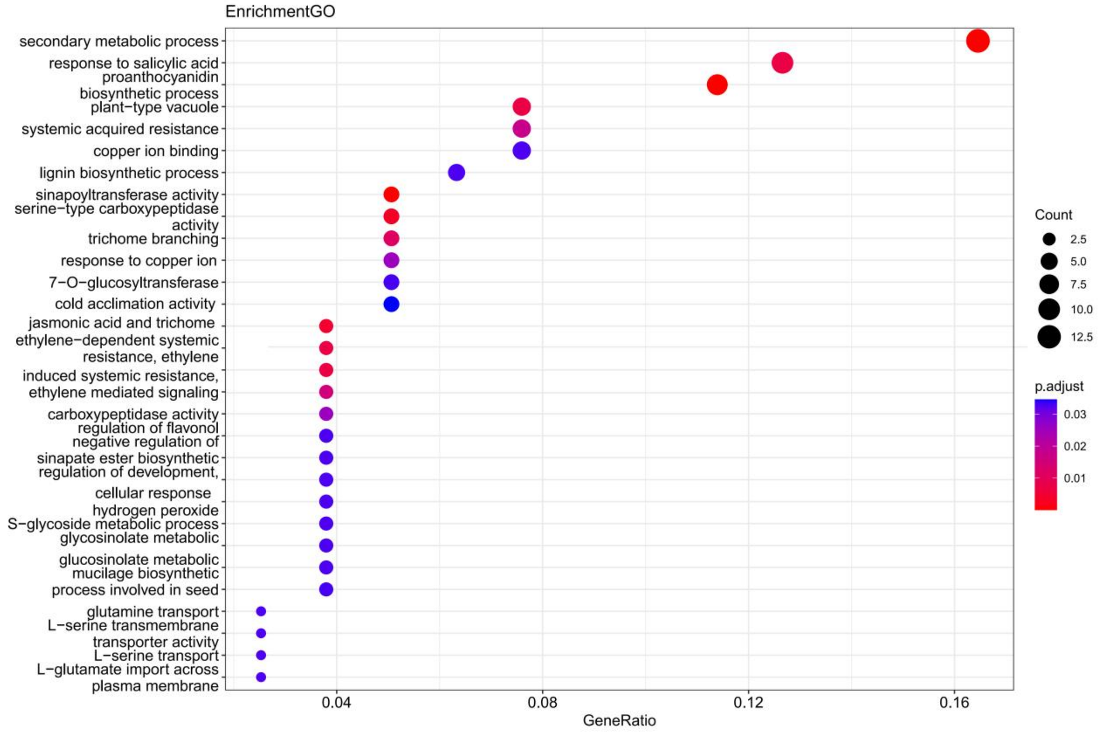

Figure S2-7 The GO enrichment dotpot of cell cluster 6 of *R. sericea*

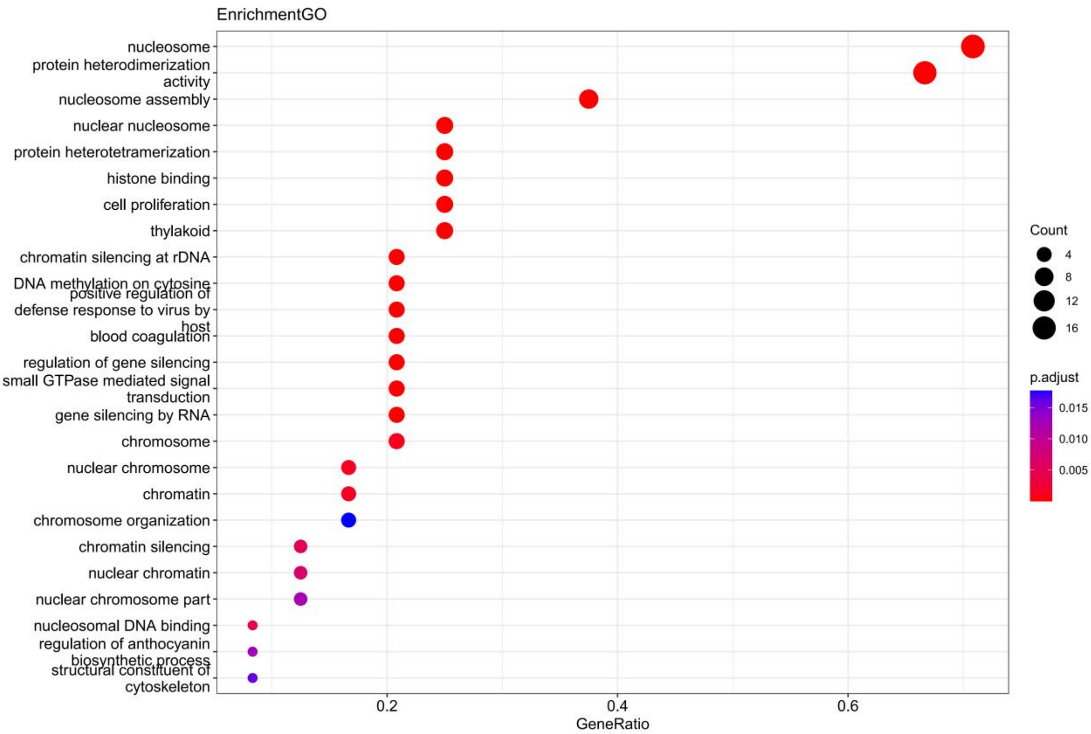

Figure S2-8 The GO enrichment dotpot of cell cluster 7 of *R. sericea*

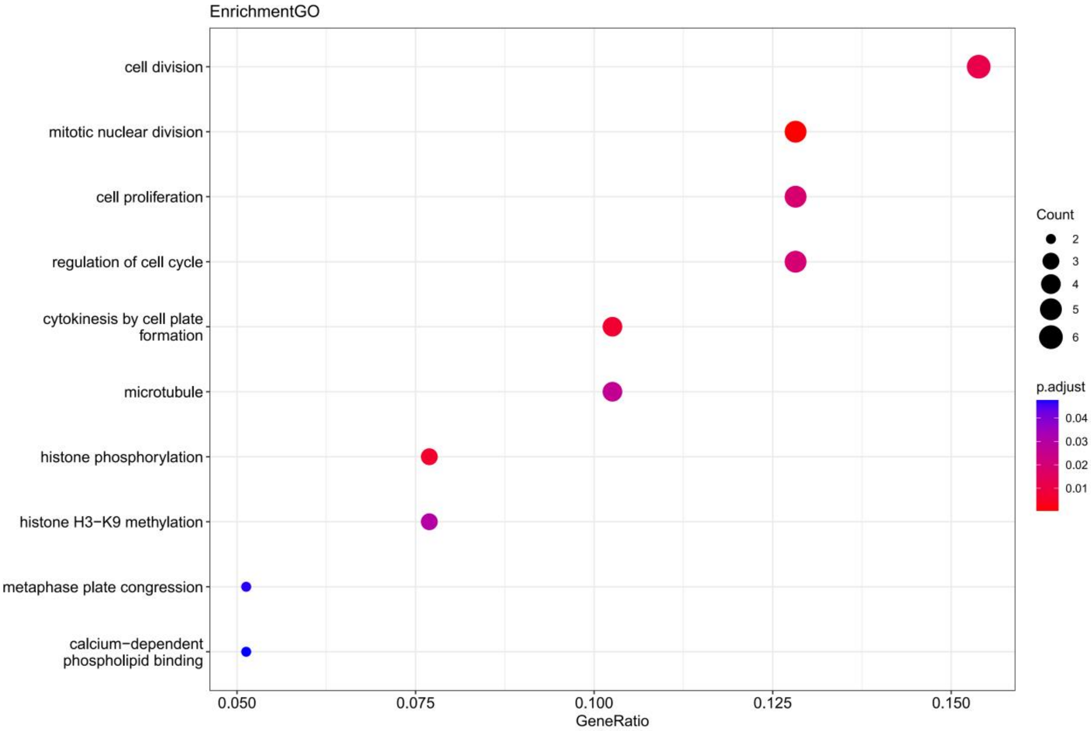

Figure S2-9 The GO enrichment dotpot of cell cluster 8 of *R. sericea*

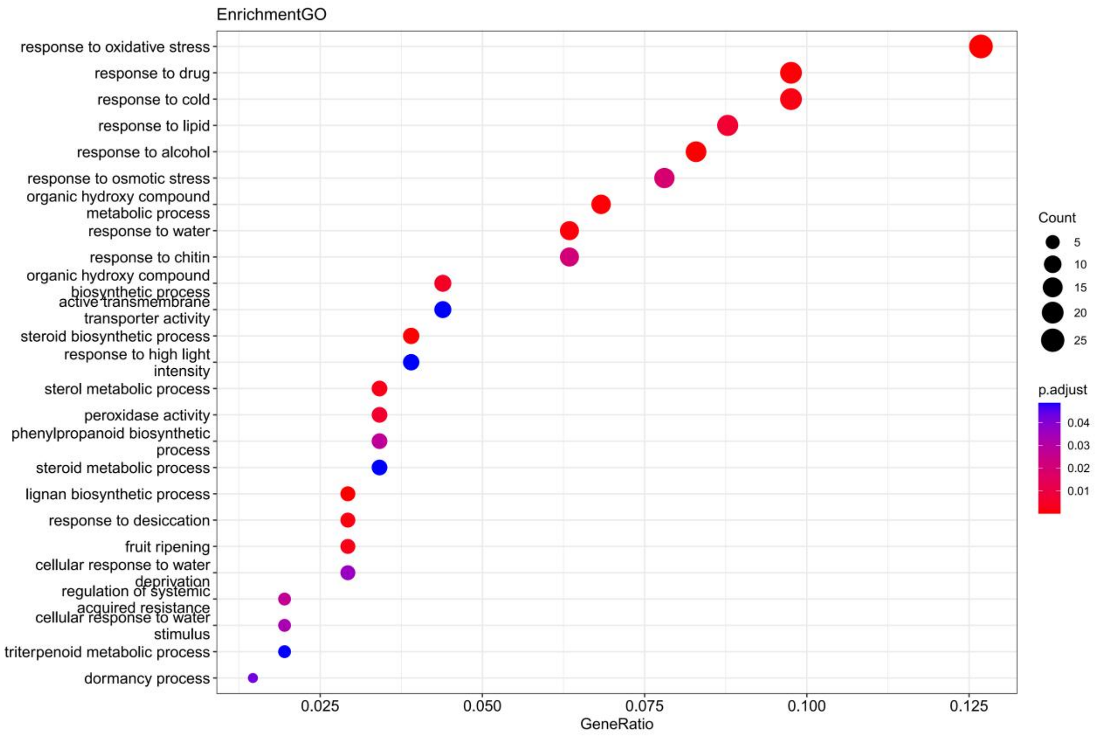

Figure S2-10 The GO enrichment dotpot of cell cluster 9 of *R. sericea*

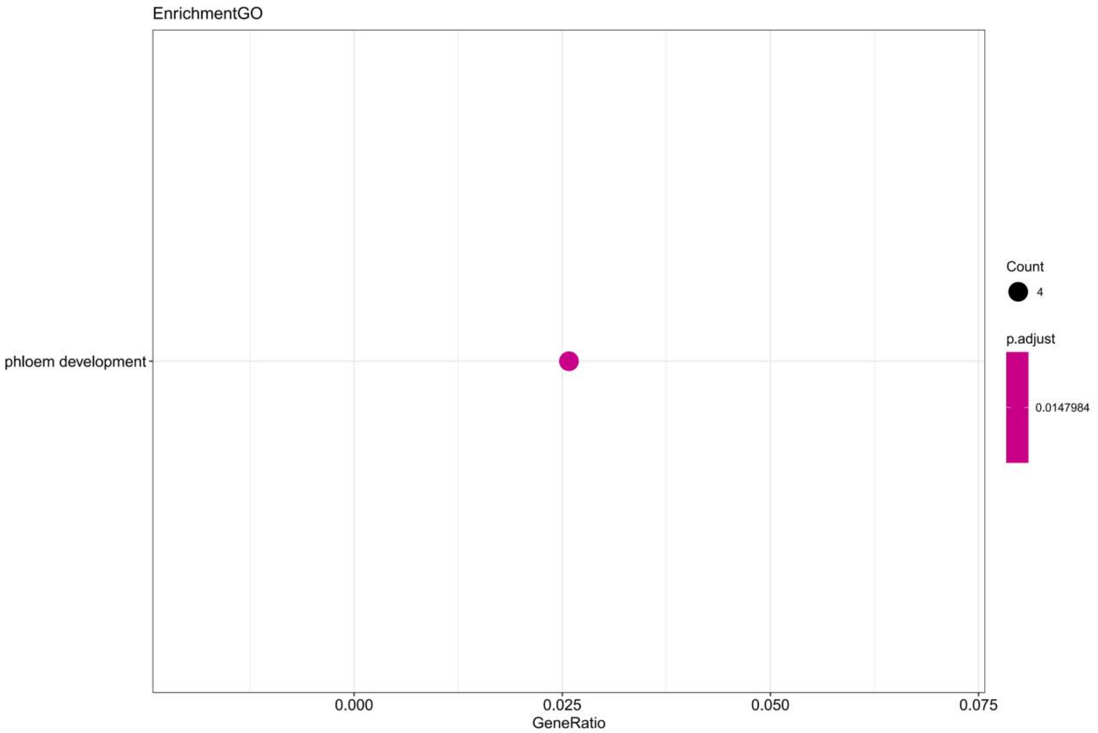

Figure S2-11 The GO enrichment dotpot of cell cluster 10 of *R. sericea*

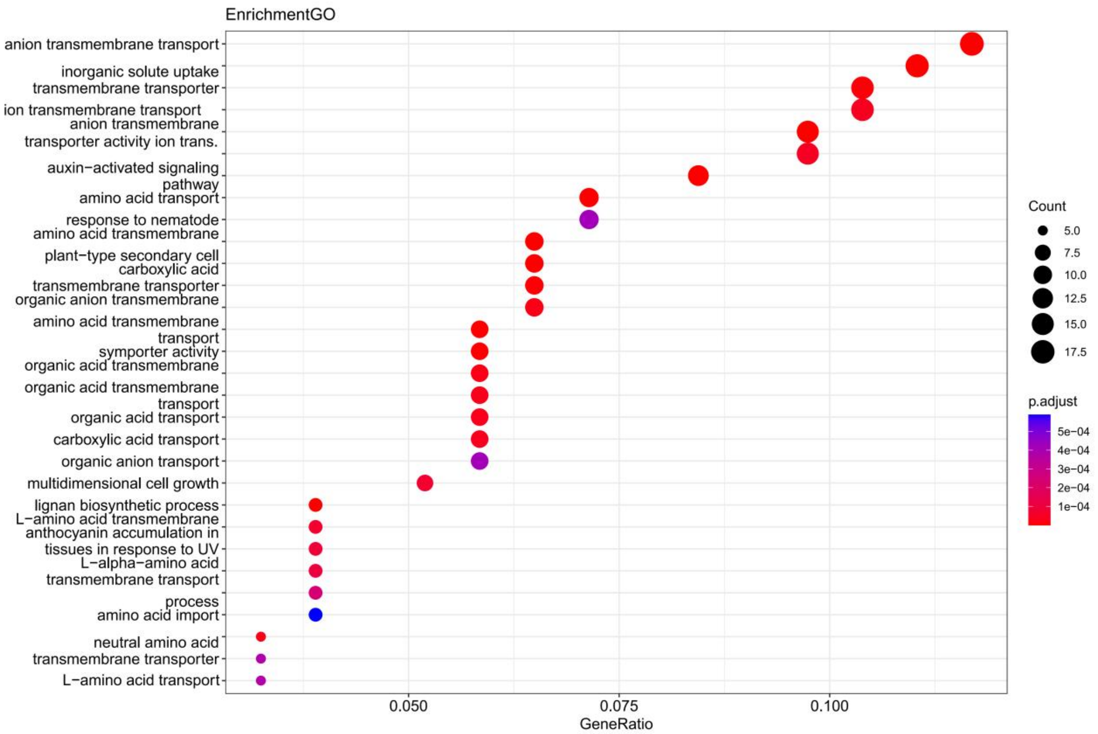

Figure S2-12 The GO enrichment dotpot of cell cluster 11 of *R. sericea*

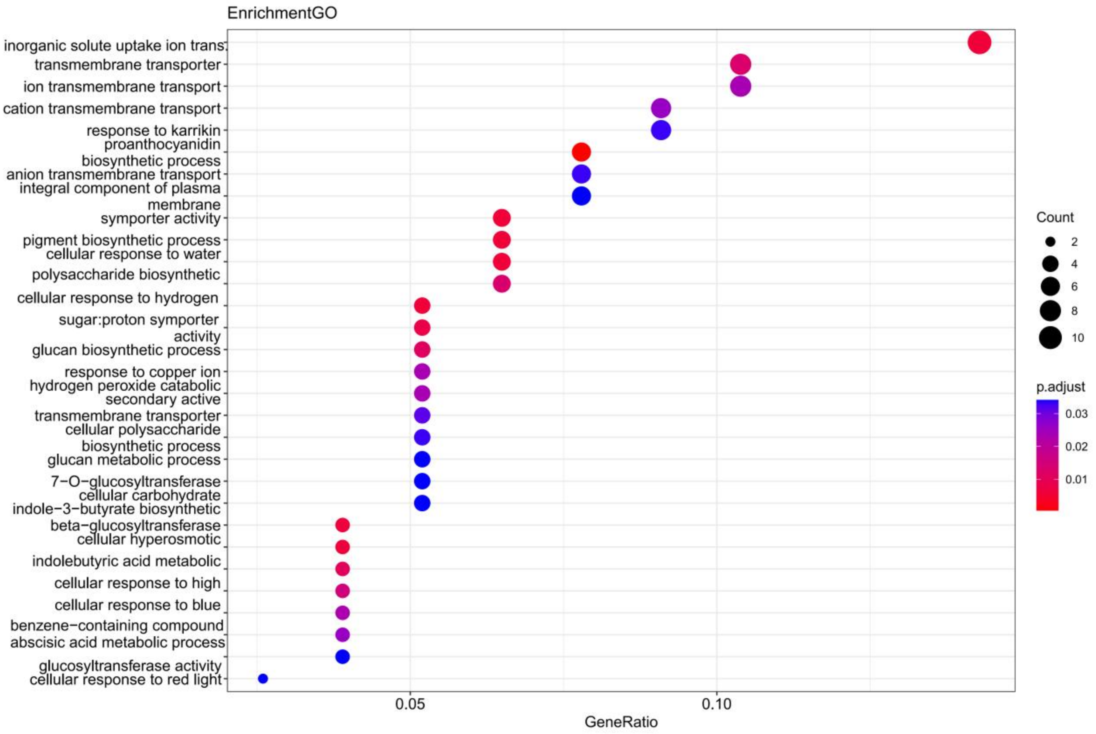

Figure S2-13 The GO enrichment dotpot of cell cluster 12 of *R. sericea*

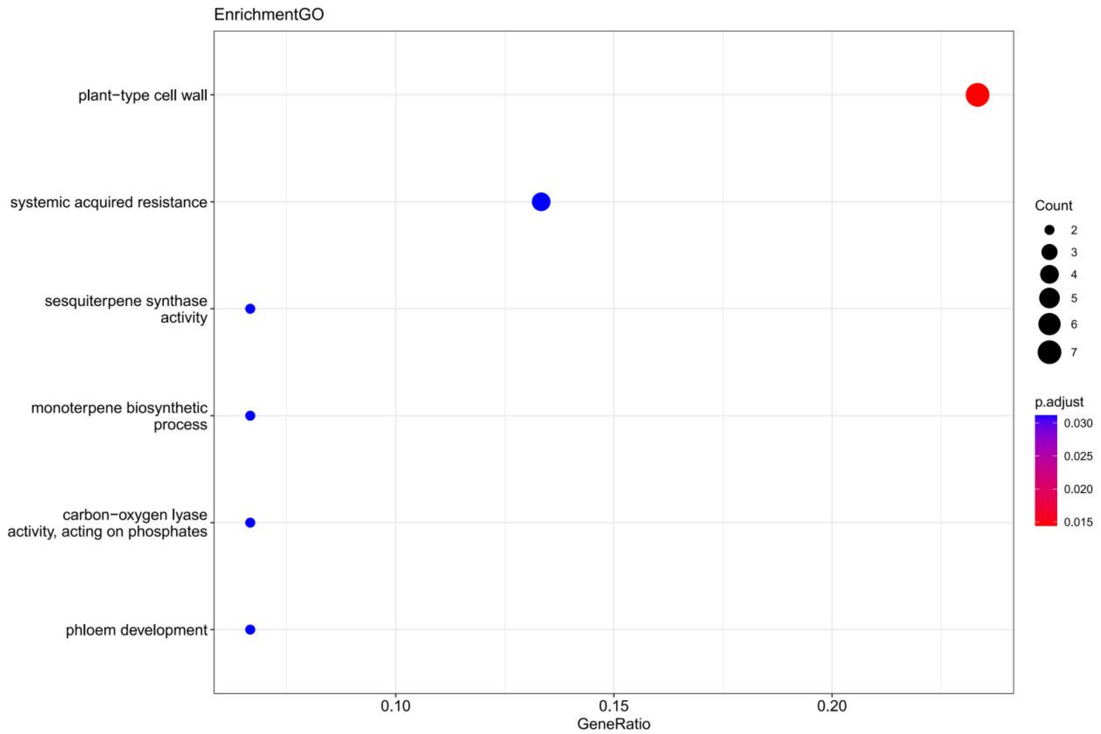

Figure S2-14 The GO enrichment dotpot of cell cluster 13 of *R. sericea*

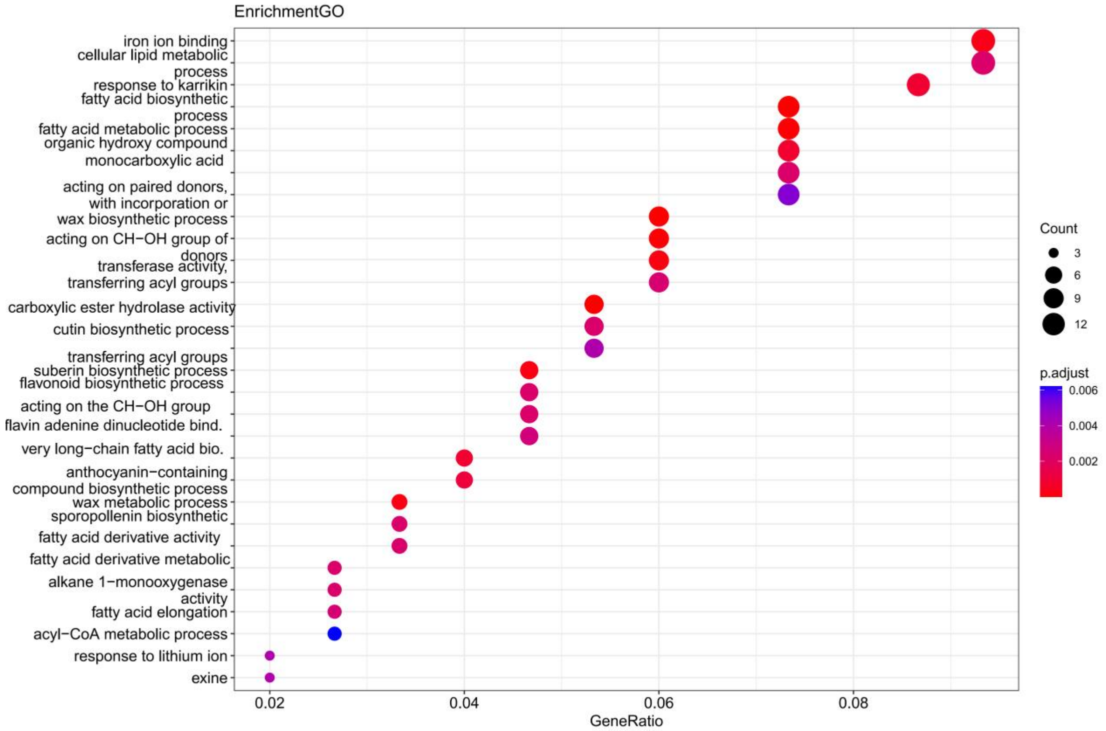

Figure S2-15 The GO enrichment dotpot of cell cluster 14 of *R. sericea*

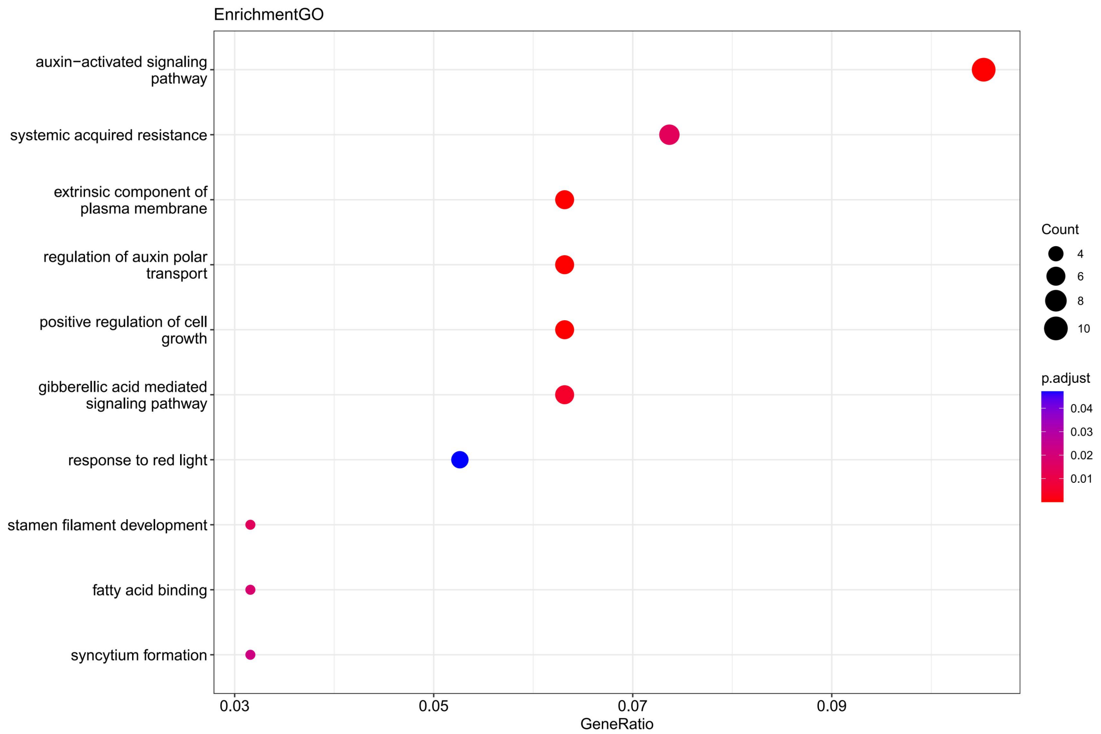

Figure S2-16 The GO enrichment dotpot of cell cluster 15 of *R. sericea*

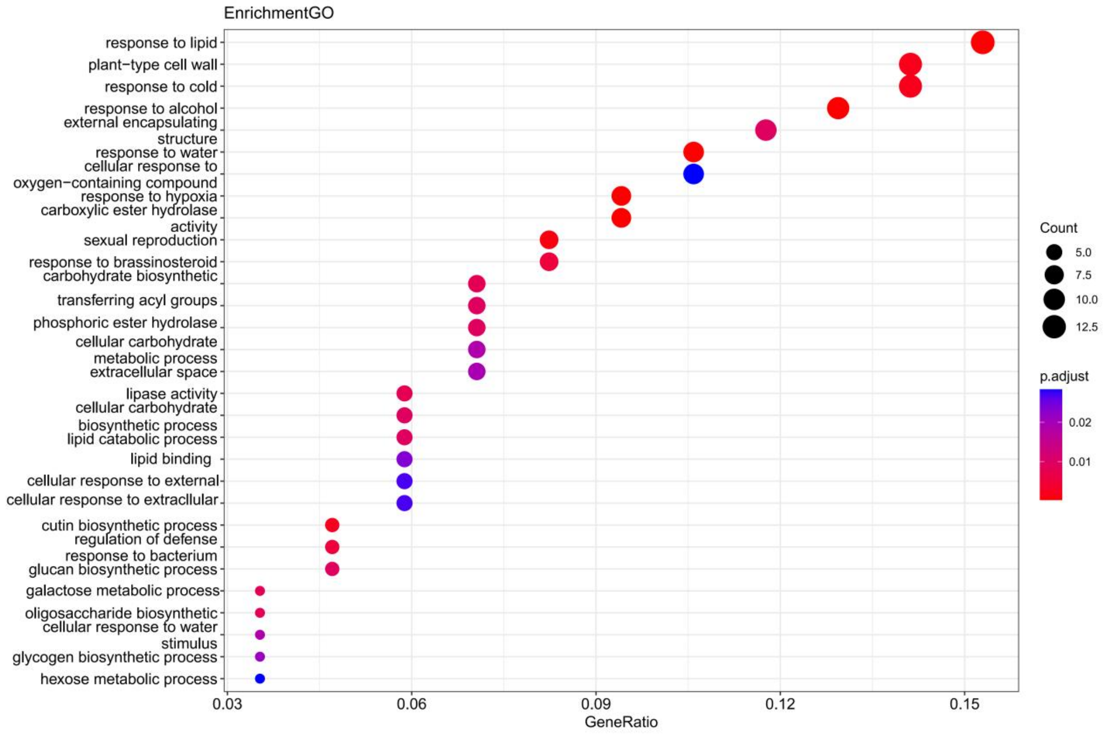

Figure S2-17 The GO enrichment dotpot of cell cluster 16 of *R. sericea*

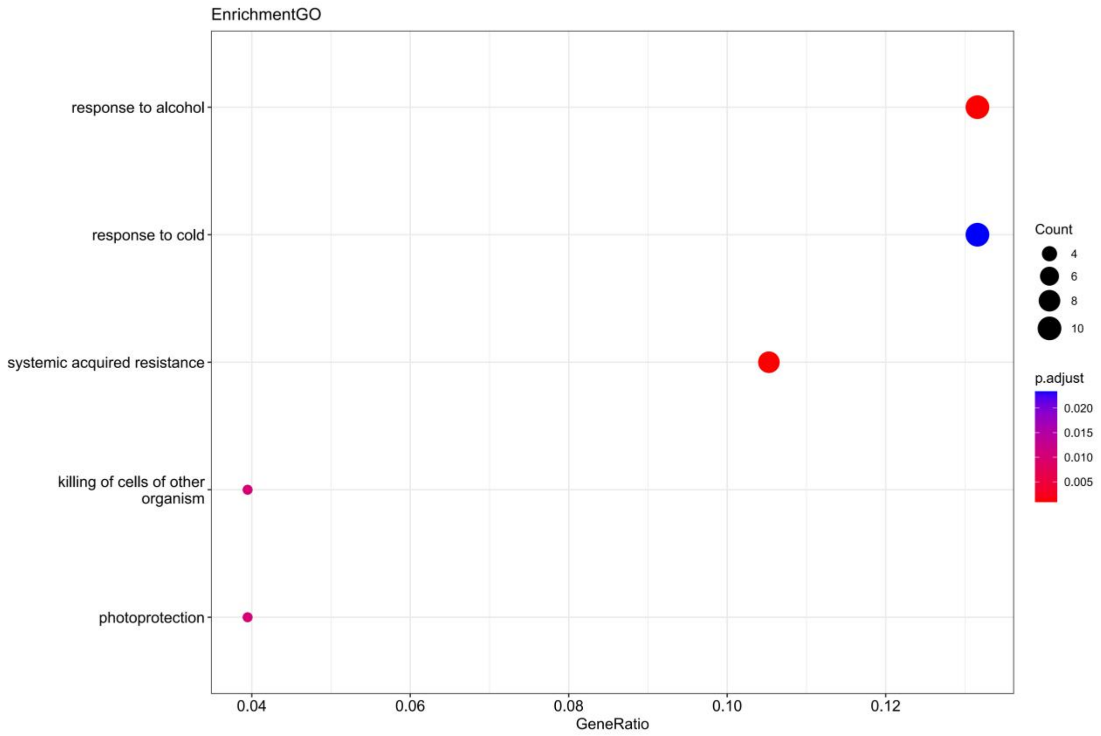

Figure S2-18 The GO enrichment dotpot of cell cluster 17 of *R. sericea*

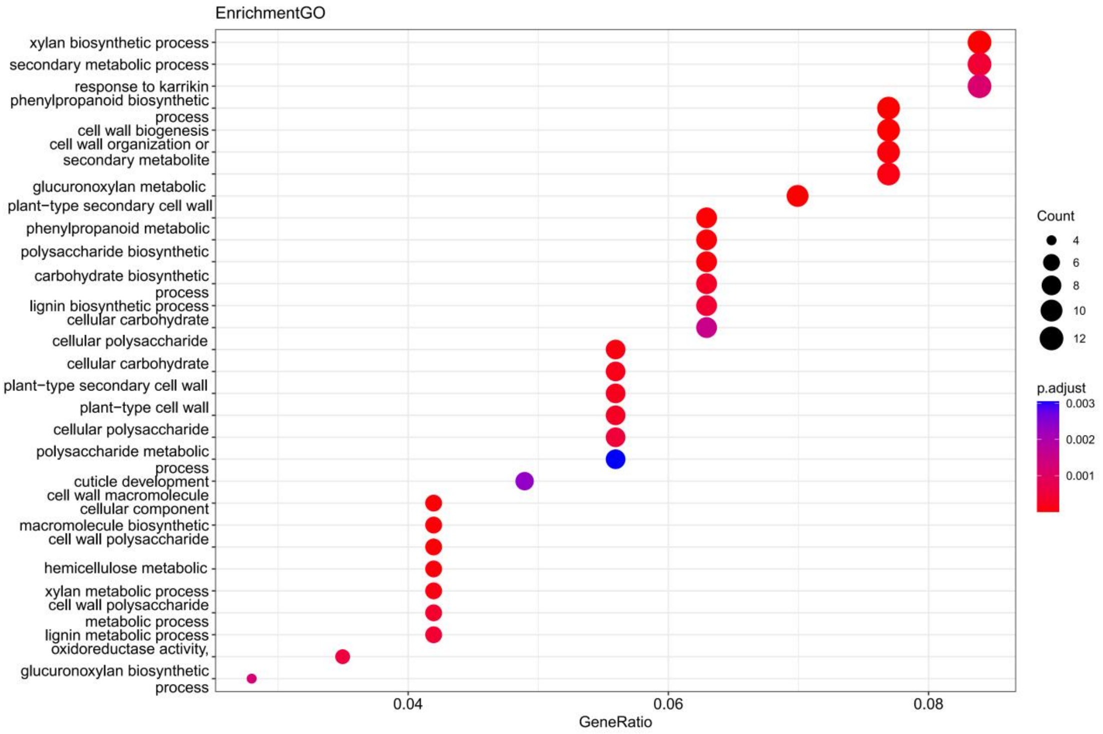

Figure S2-19 The GO enrichment dotpot of cell cluster 18 of *R. sericea*

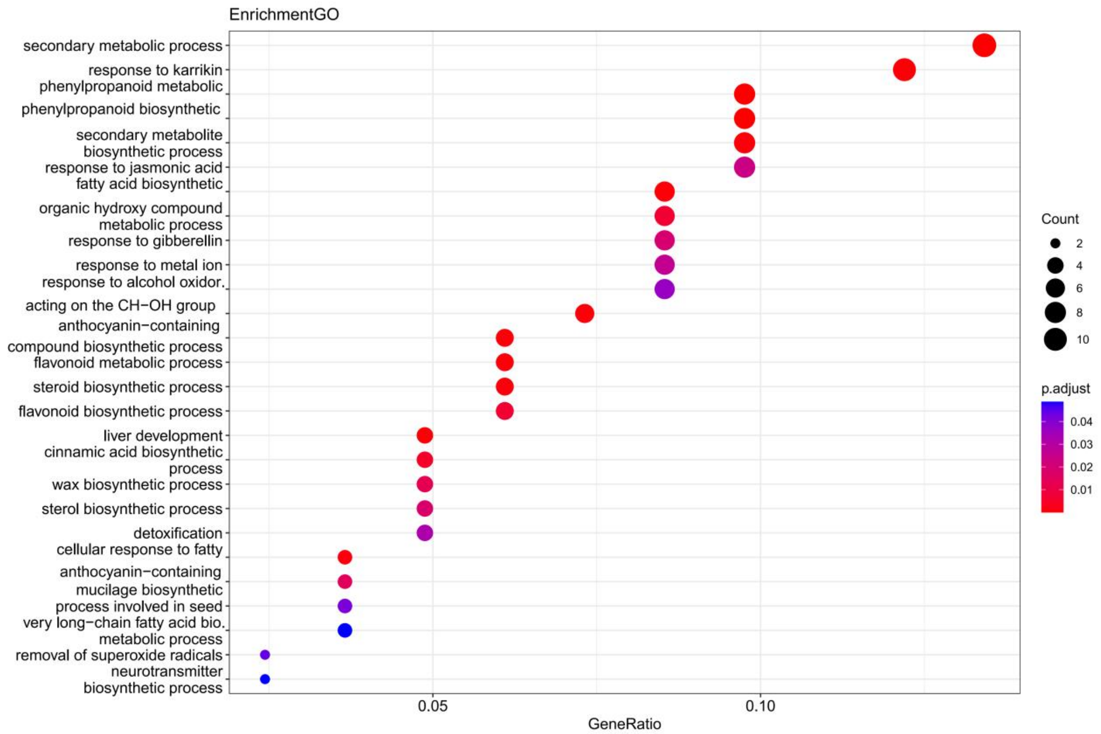

Figure S2-20 The GO enrichment dotpot of cell cluster 20 of *R. sericea*

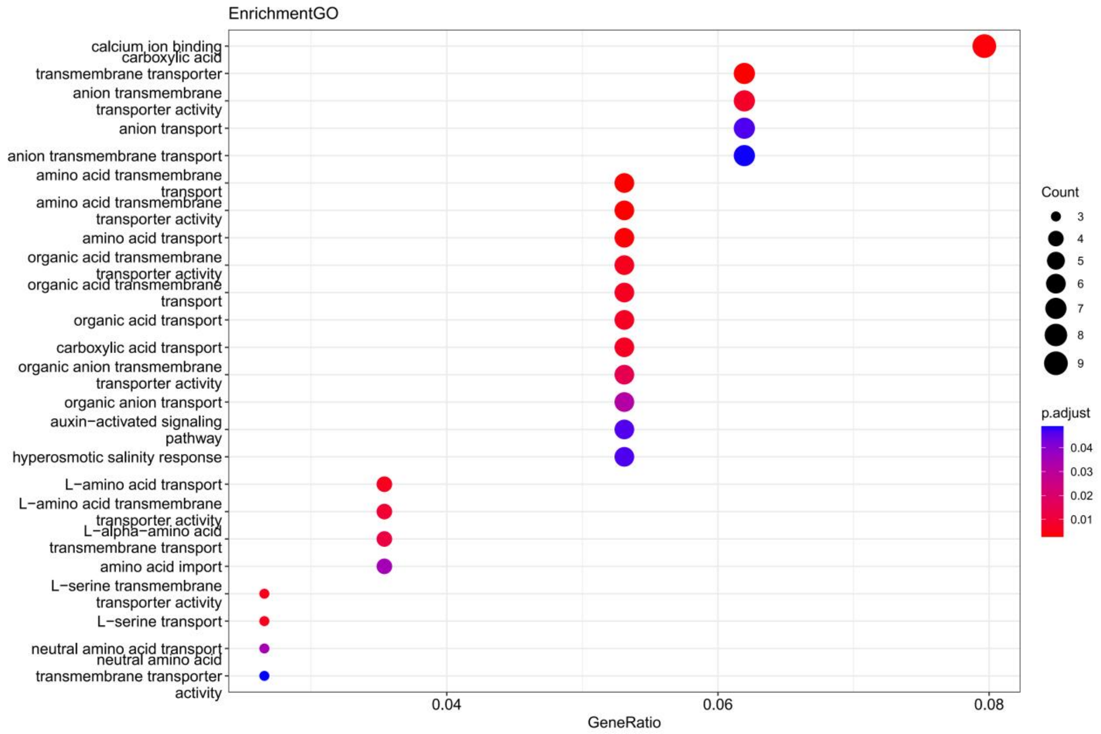

Figure S2-21 The GO enrichment dotpot of cell cluster 20 of *R. sericea*

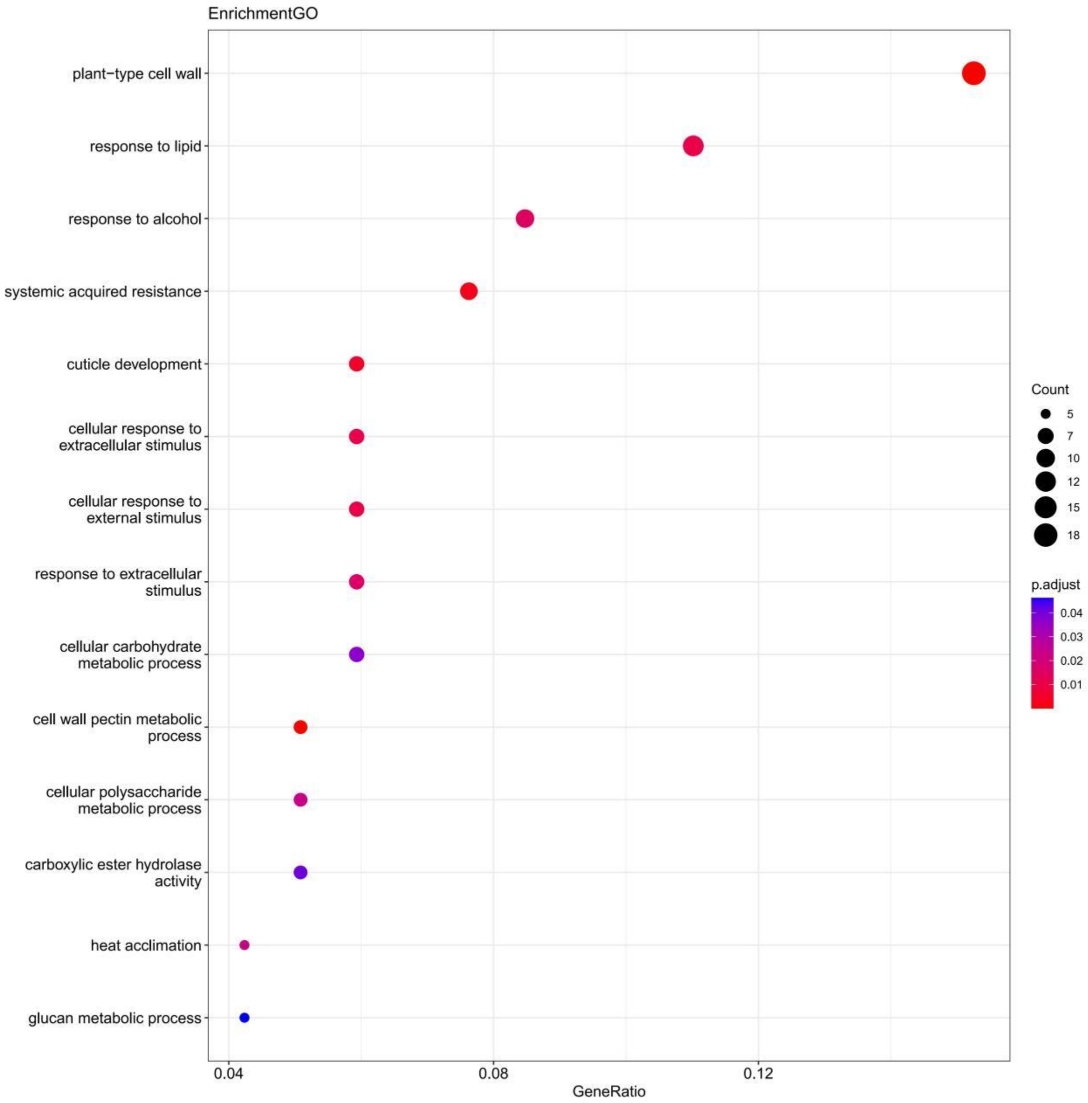

Figure S2-22 The GO enrichment dotpot of cell cluster 21 of *R. sericea*

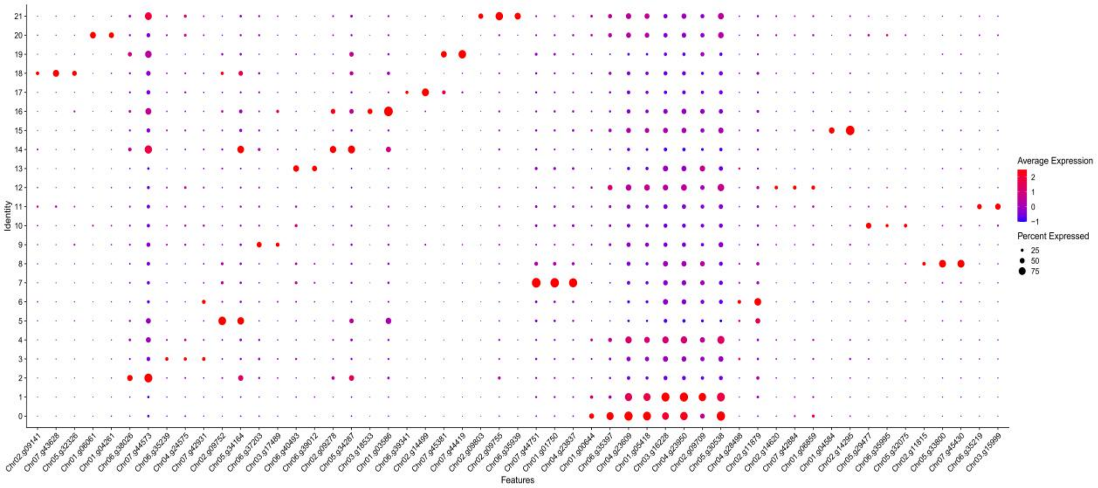

Figure S3-1 The expression of known marker genes across each cell cluster was shown, with dot size corresponding to the percentage of cells expressing the gene. The color of each dot reflected the relative expression level of the gene within each cell type, with warmer colors indicating higher expression levels

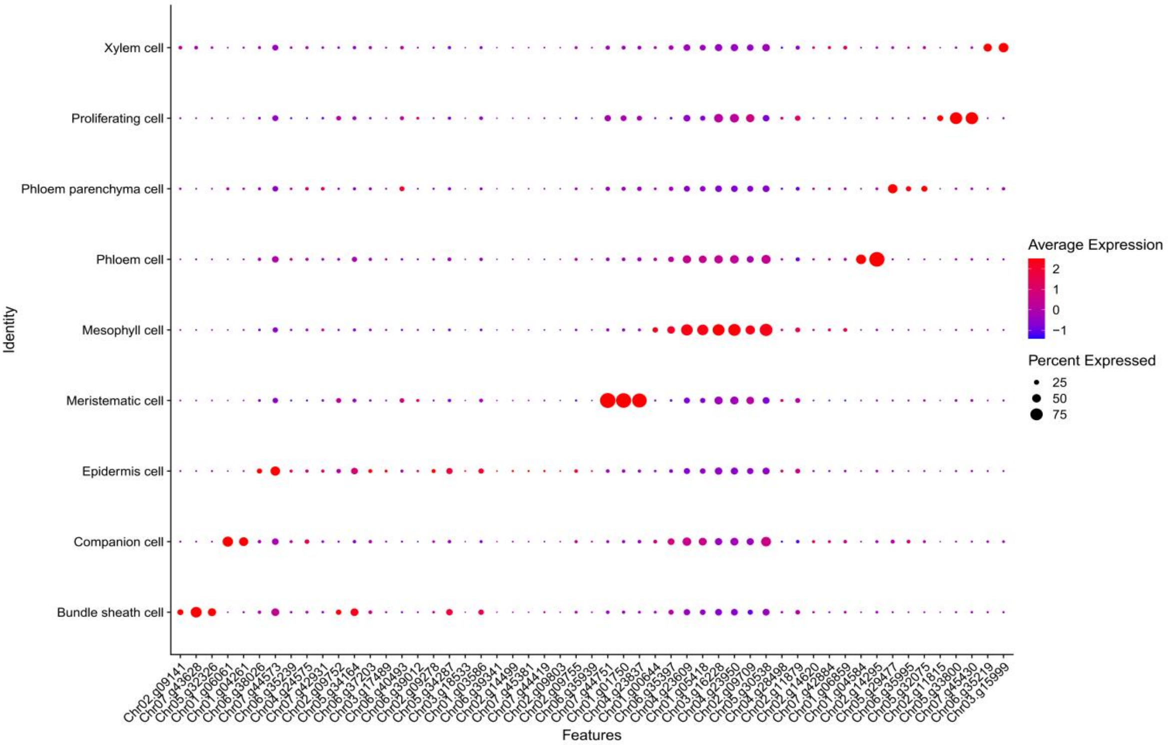

Figure S3-2 The expression of known marker genes across each cell type was shown, with dot size corresponding to the percentage of cells expressing the gene. The color of each dot reflected the relative expression level of the gene within each cell type, with warmer colors indicating higher expression levels

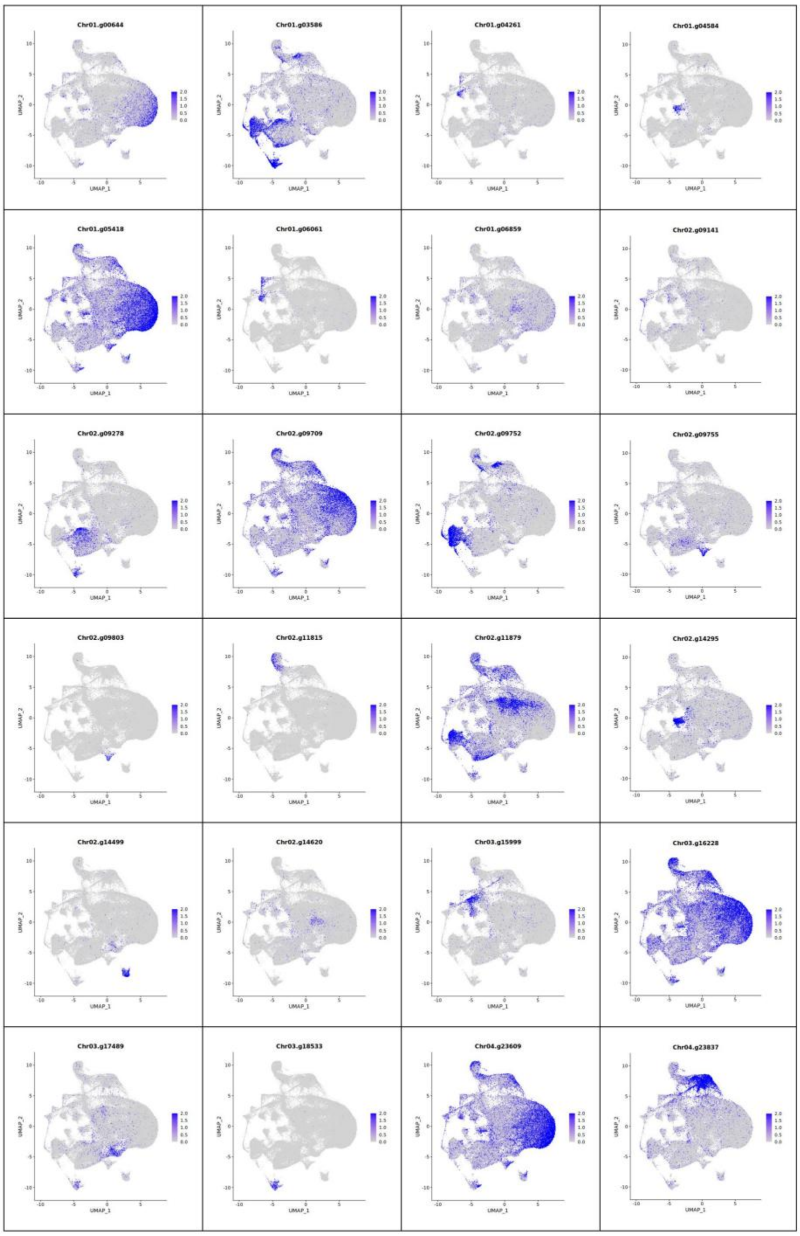

Figure S4-1 the UMAP dotplot of the first 24 marker genes for cell type annotation in *R. sericea* leaves

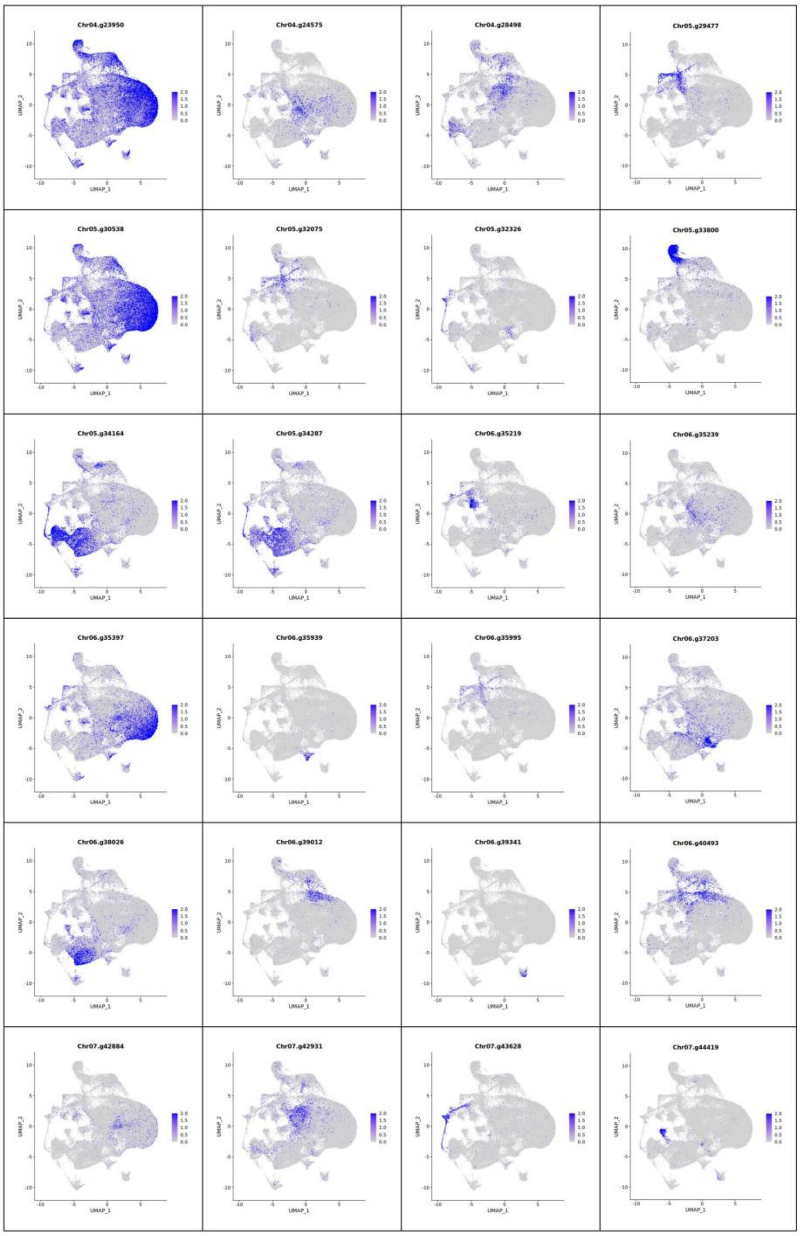

Figure S4-2 the UMAP dotplot of other 24 marker genes for cell type annotation in *R. sericea* leaves

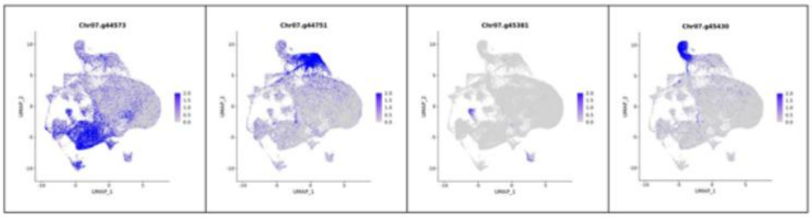

Figure S4-3 the UMAP dotplot of the last 4 marker genes for cell type annotation in *R. sericea* leaves

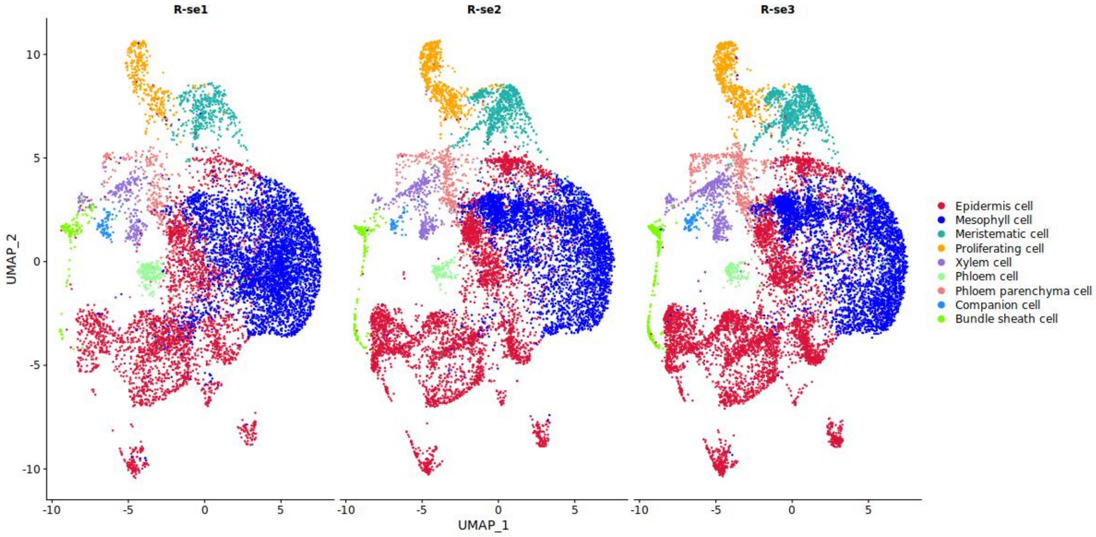

Figure S5 The classification of distinct cell clusters was illustrated in the UMAP plot, where each dot represented an individual cell, colored according to its respective cell type. R-se1, R-se2, and R-se3 represent biological replicate 1, biological replicate 2, and biological replicate 3, respectively.

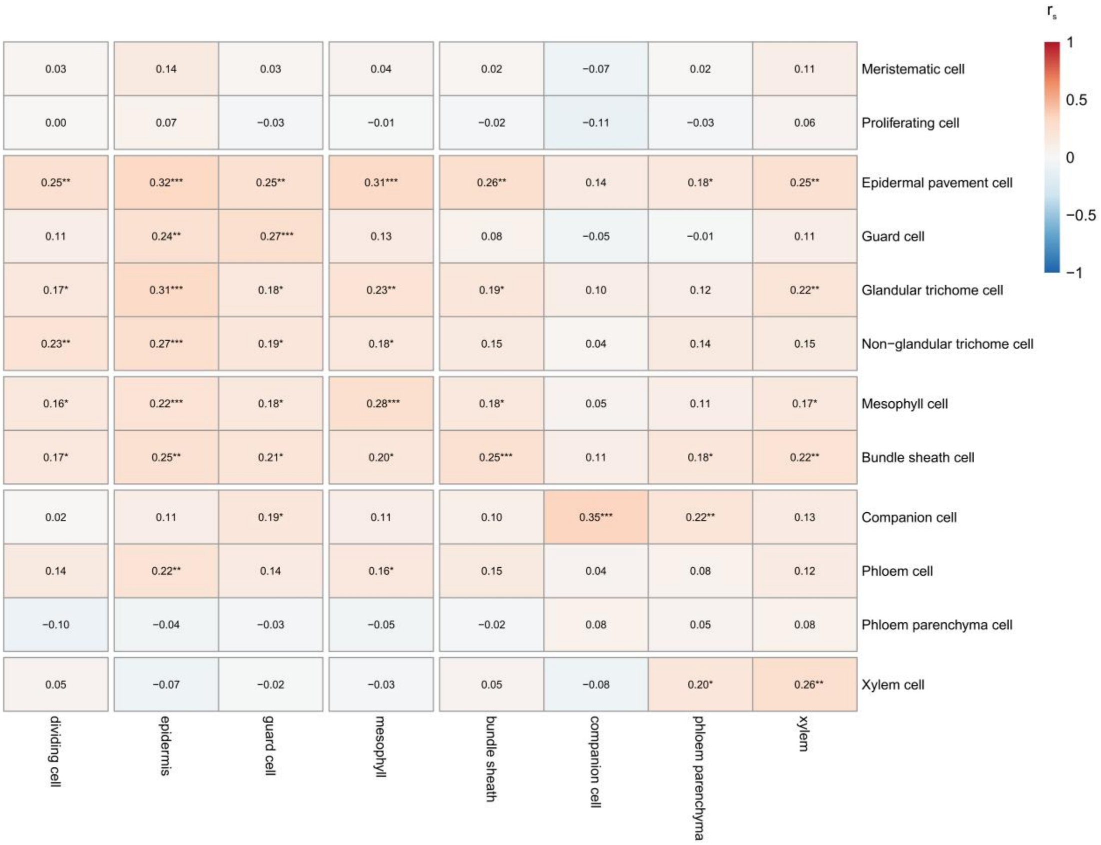

Figure S6 Heatmap showing cross-species comparison of single-cell type annotation profiles between *R. sericea* and *Arabidopsis thaliana*. The absolute Spearman correlation coefficients (r_s), together with their associated statistical significance (p values) are displayed on the map (* $p < 0.05$, ** $p < 0.01$, *** $p < 0.001$). The cell classifications on the right side of the map are from *R. sericea*, and the cell classifications below are from *A. thaliana* reference data.

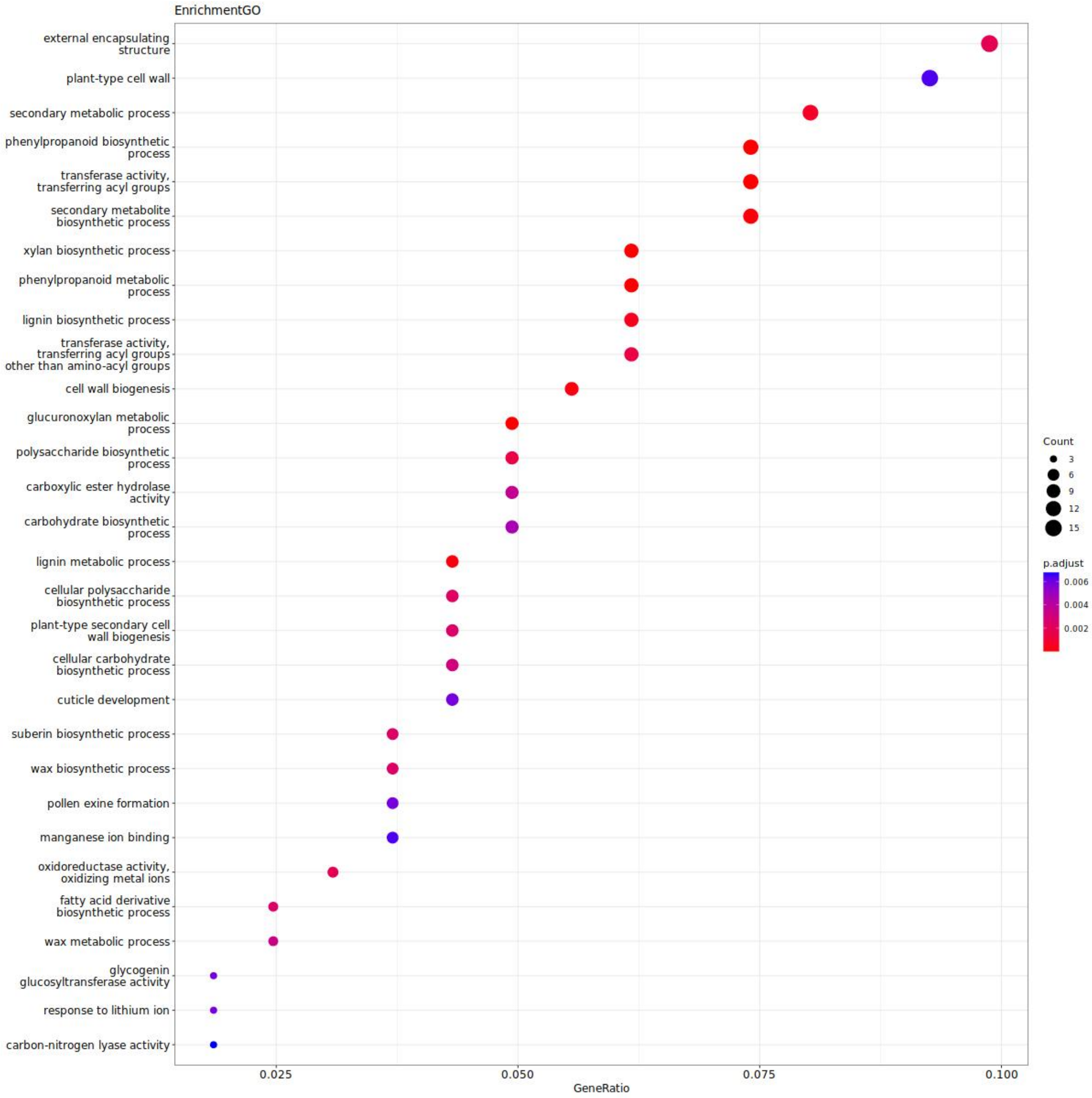

Figure S7-1 The GO enrichment dotpot of Bundle sheath cell of *R. sericea*

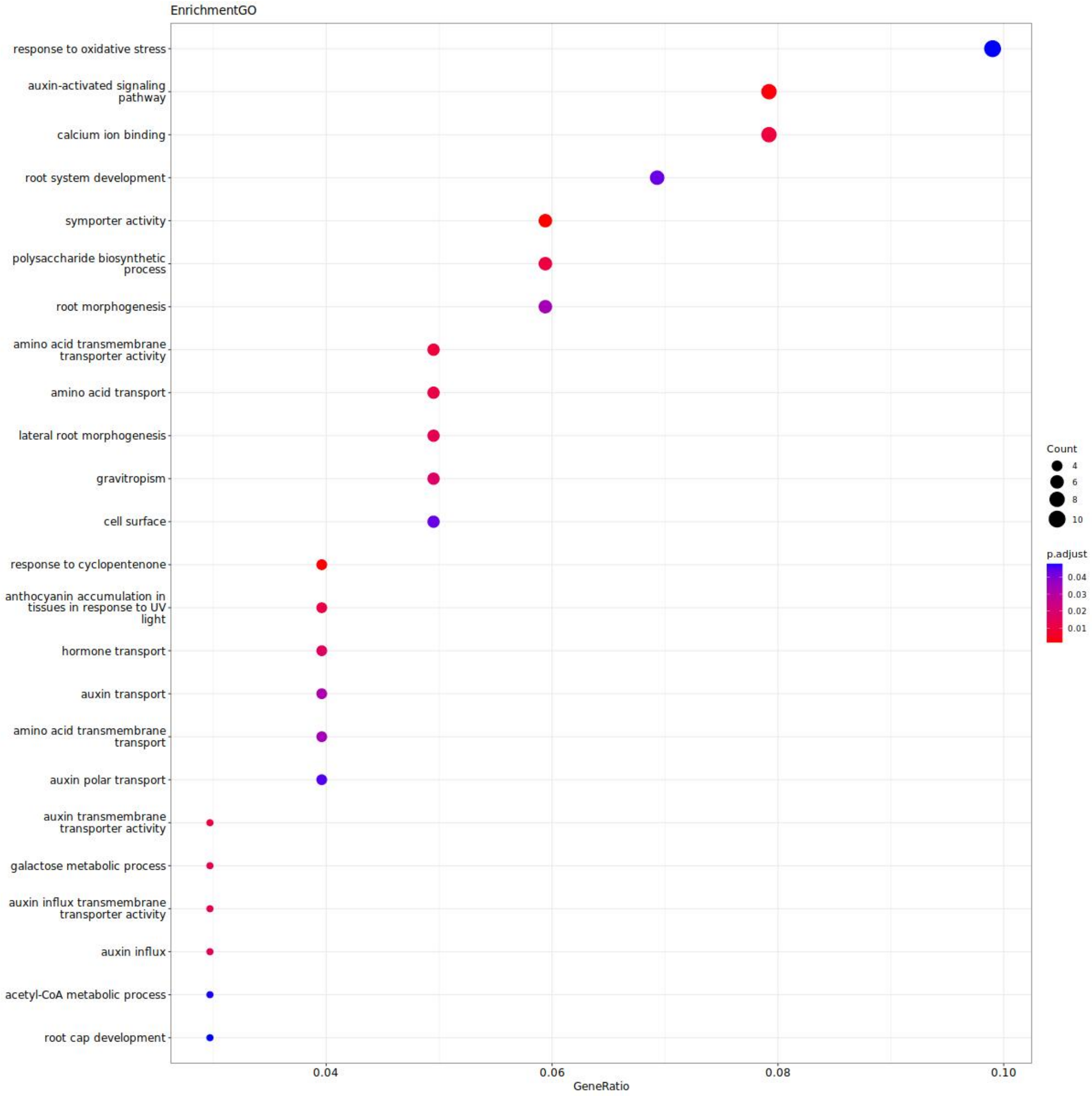

Figure S7-2 The GO enrichment dotpot of Companion cell of *R. sericea*

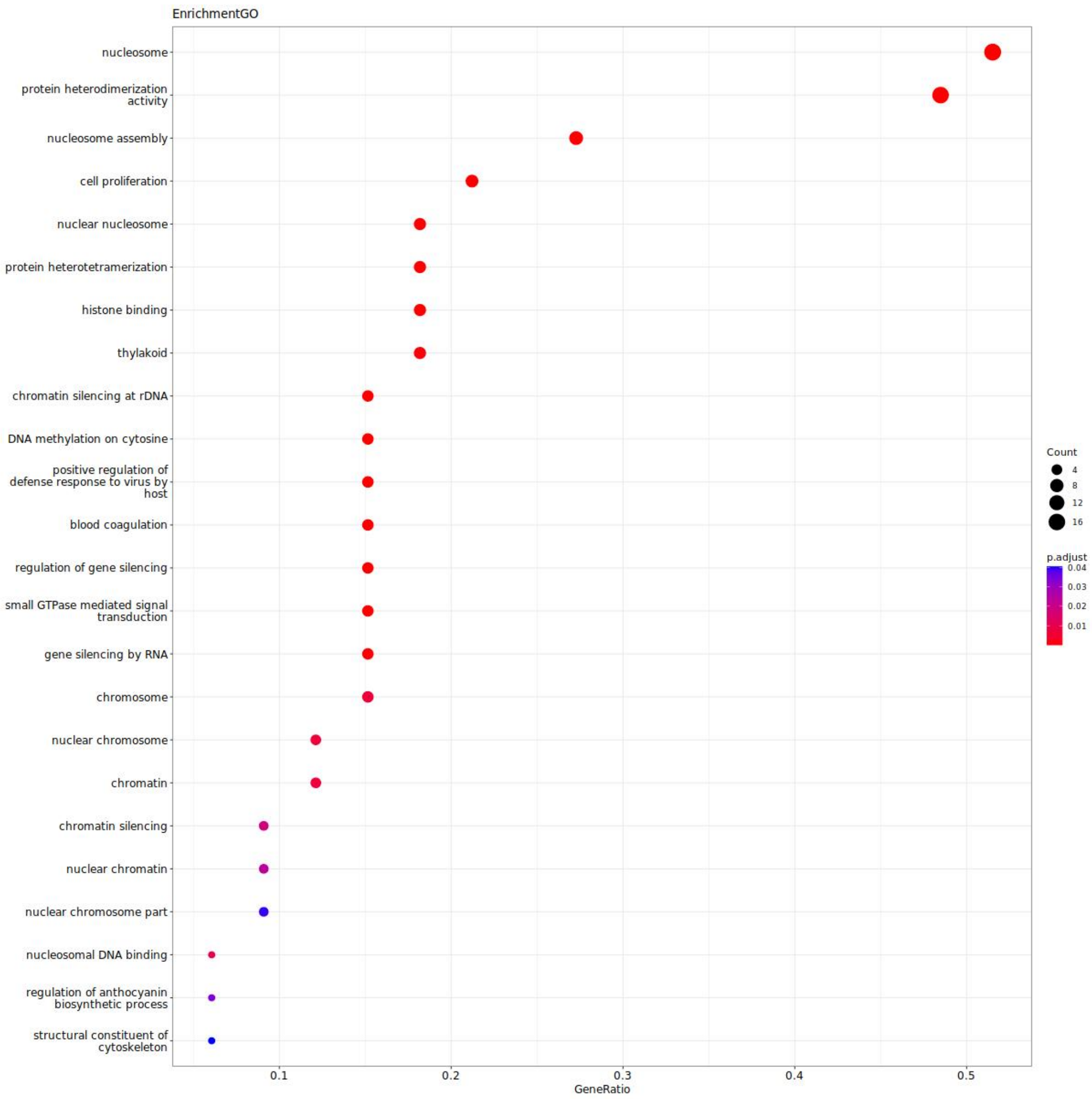

Figure S7-3 The GO enrichment dotpot of Meristematic cell of *R. sericea*

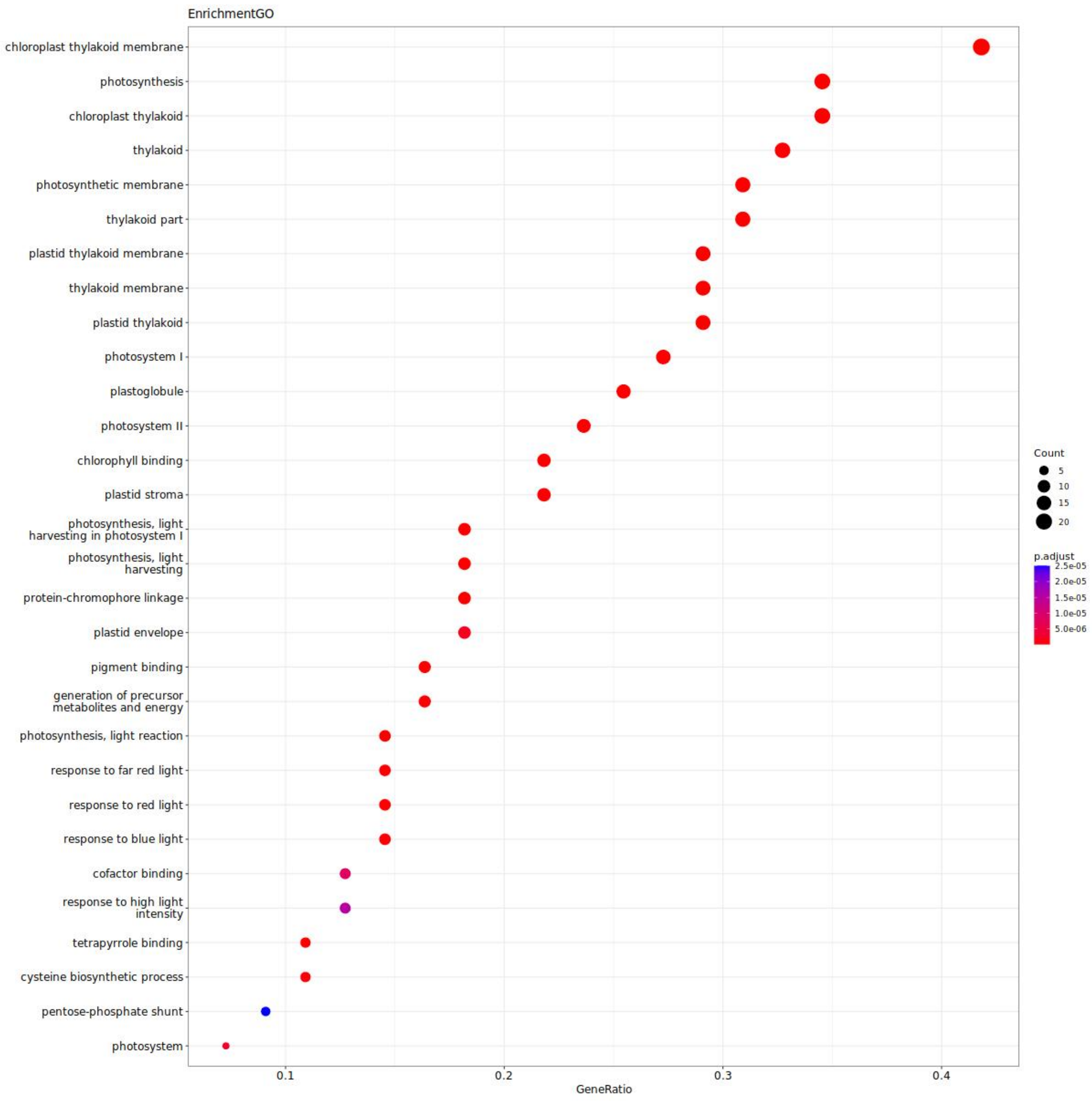

Figure S7-4 The GO enrichment dotpot of Mesophyll cell of *R. sericea*

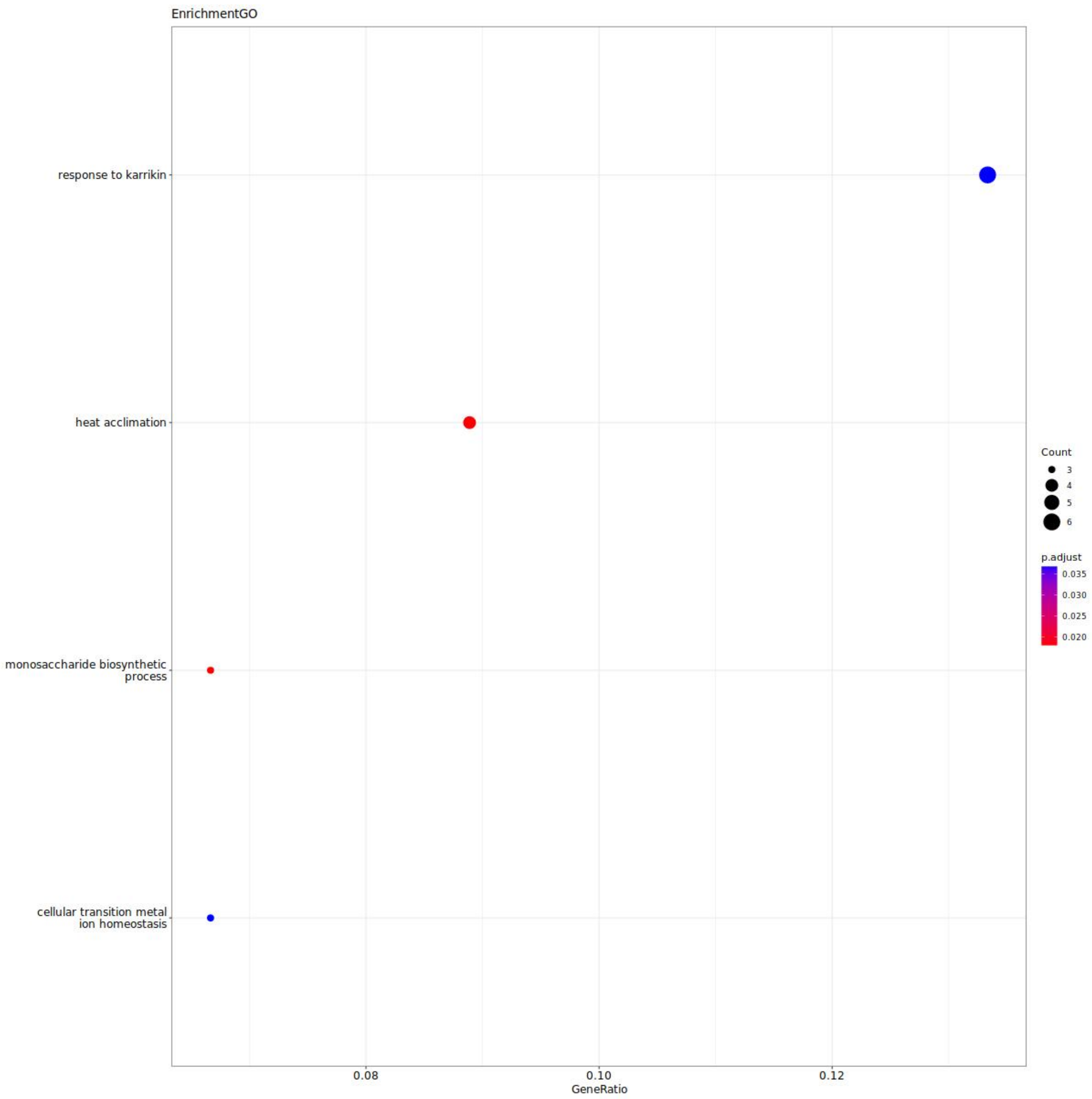

Figure S7-5 The GO enrichment dotpot of Phloem cell of *R. sericea*

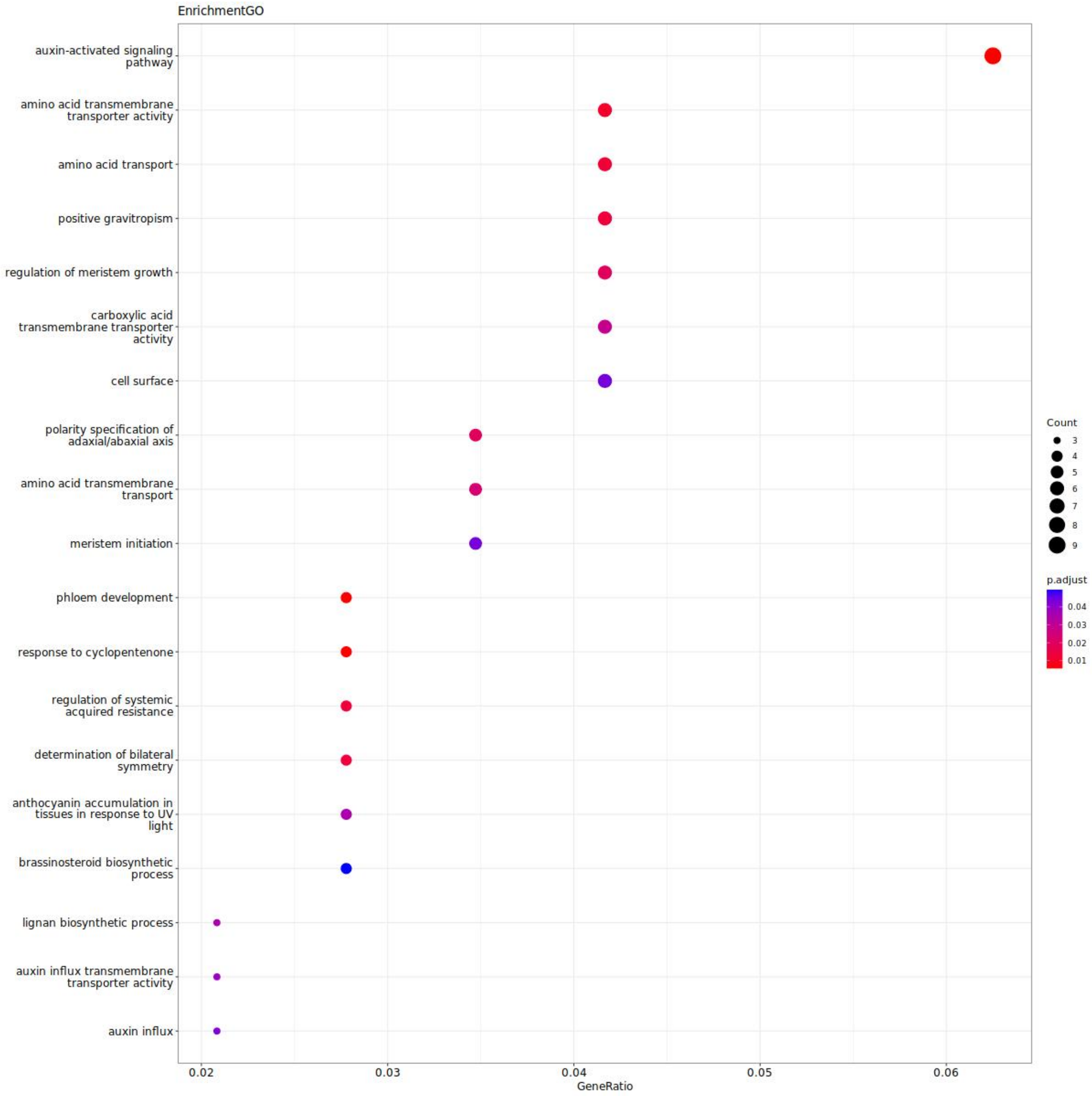

Figure S7-6 The GO enrichment dotpot of Phloem parenchyma cell of *R. sericea*

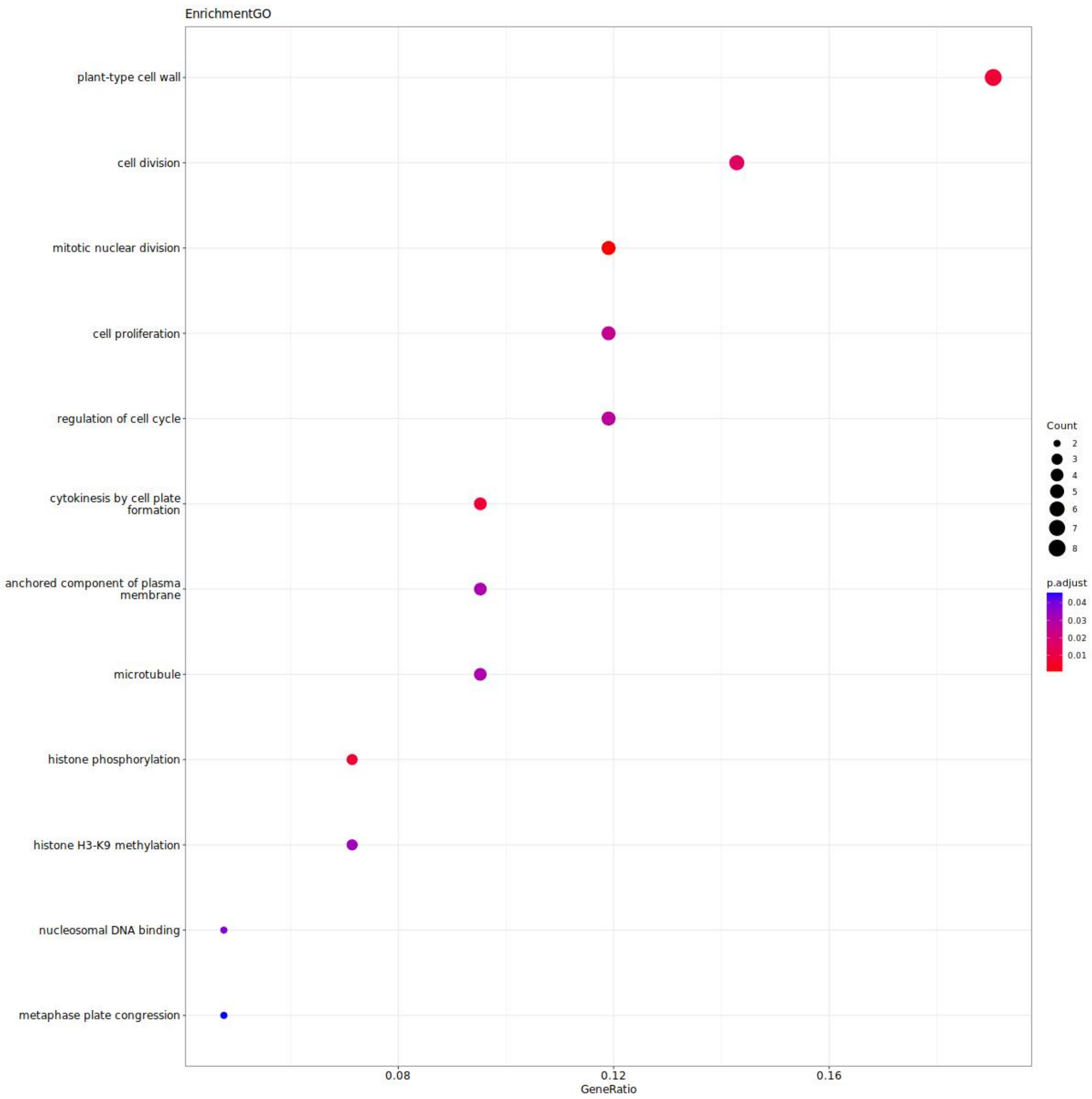

Figure S7-7 The GO enrichment dotpot of Proliferating cell of *R. sericea*

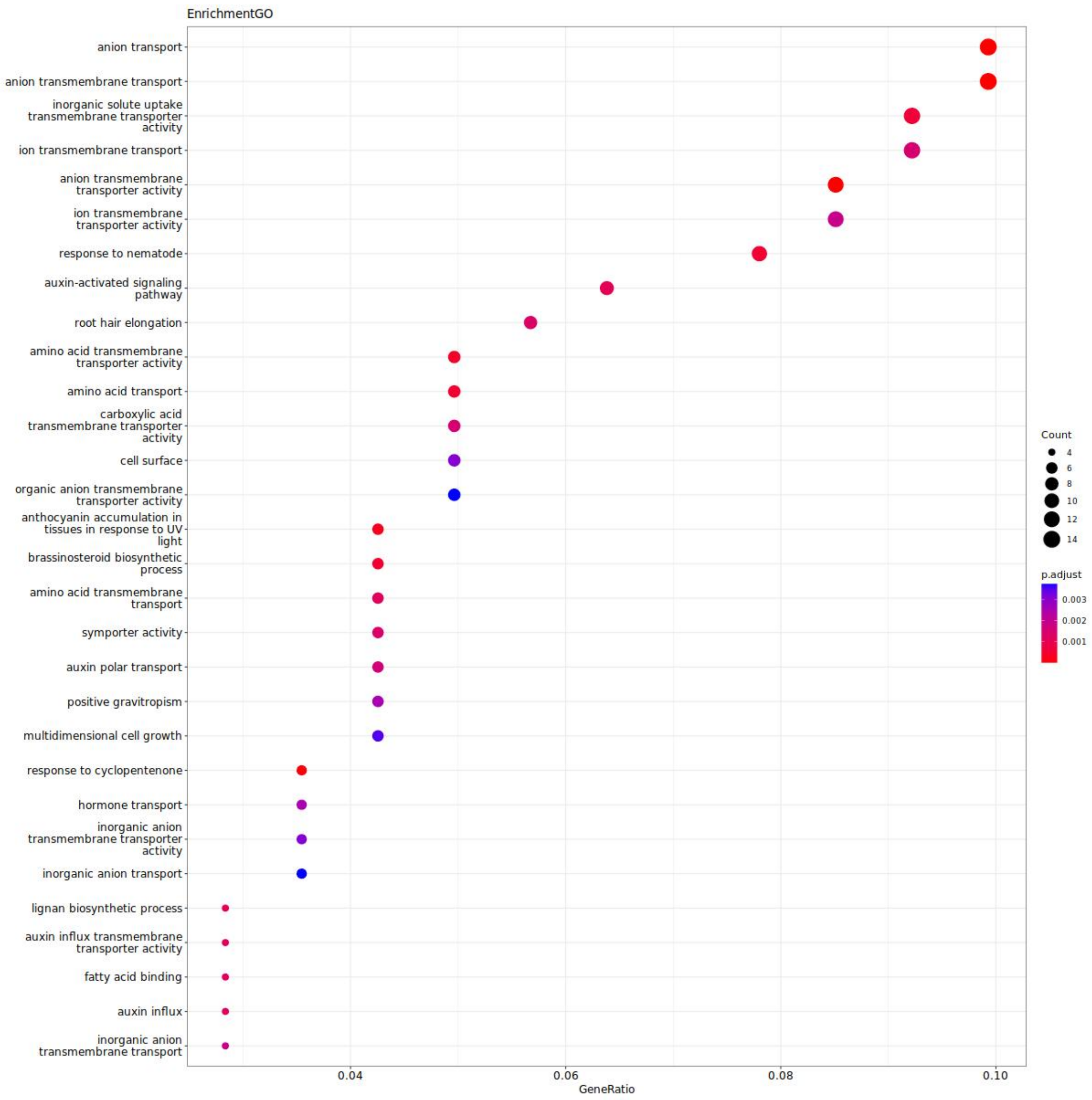

Figure S7-8 The GO enrichment dotpot of Xylem cell of *R. sericea*

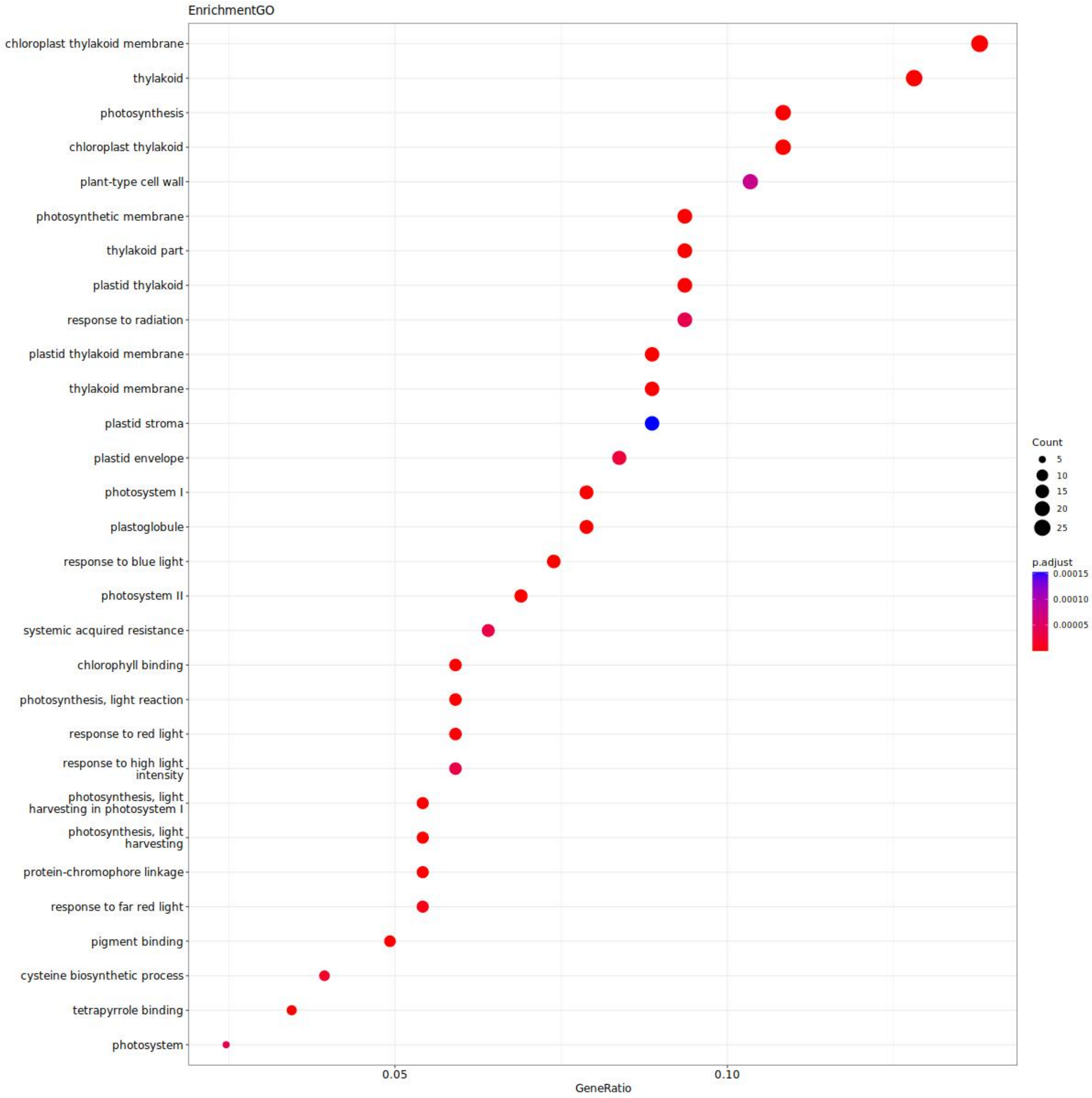

Figure S7-9 The GO enrichment dotpot of Epidermal-pavement-cell of *R. sericea*

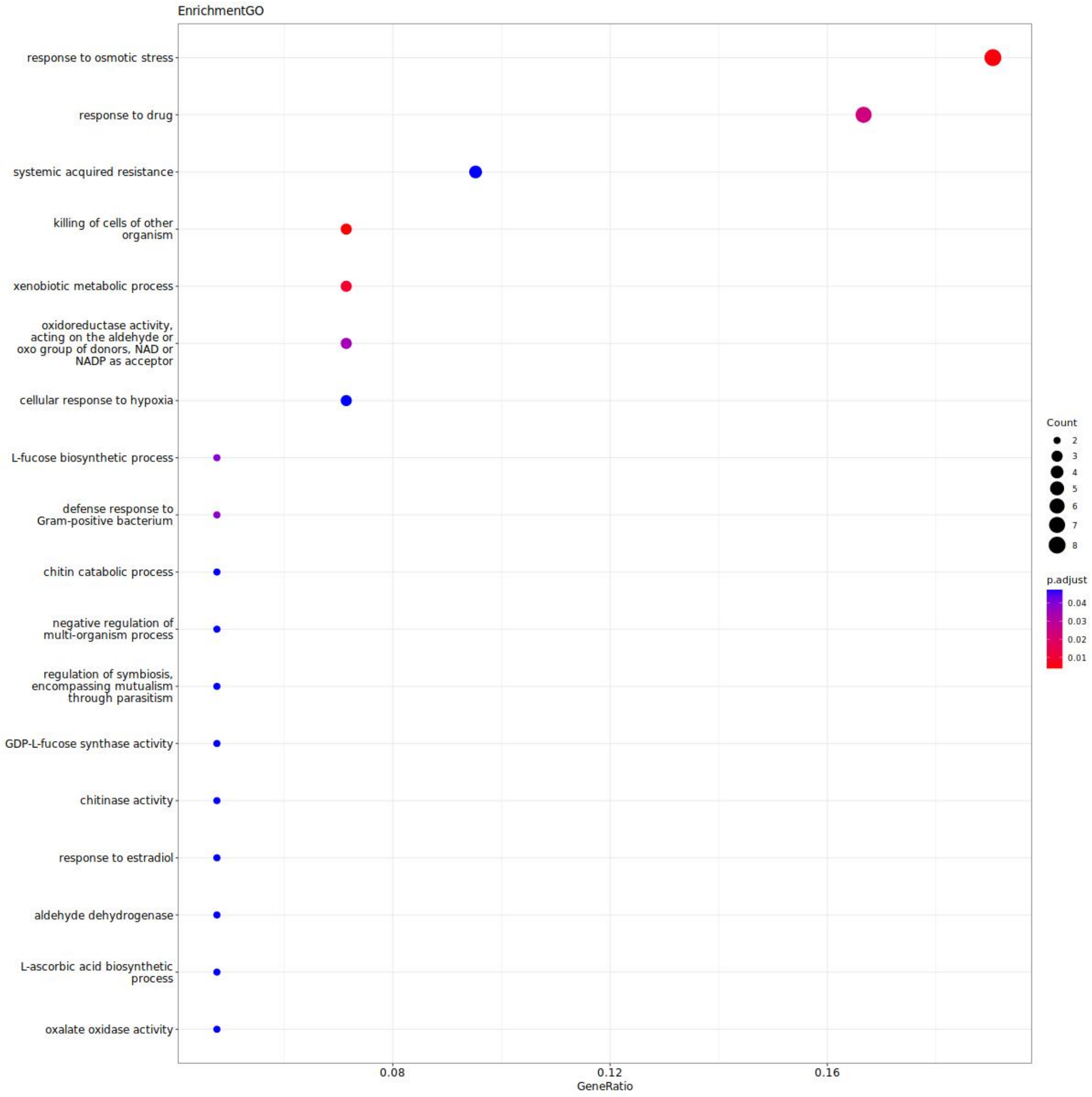

Figure S7-10 The GO enrichment dotpot of Trichome cell of *R. sericea*

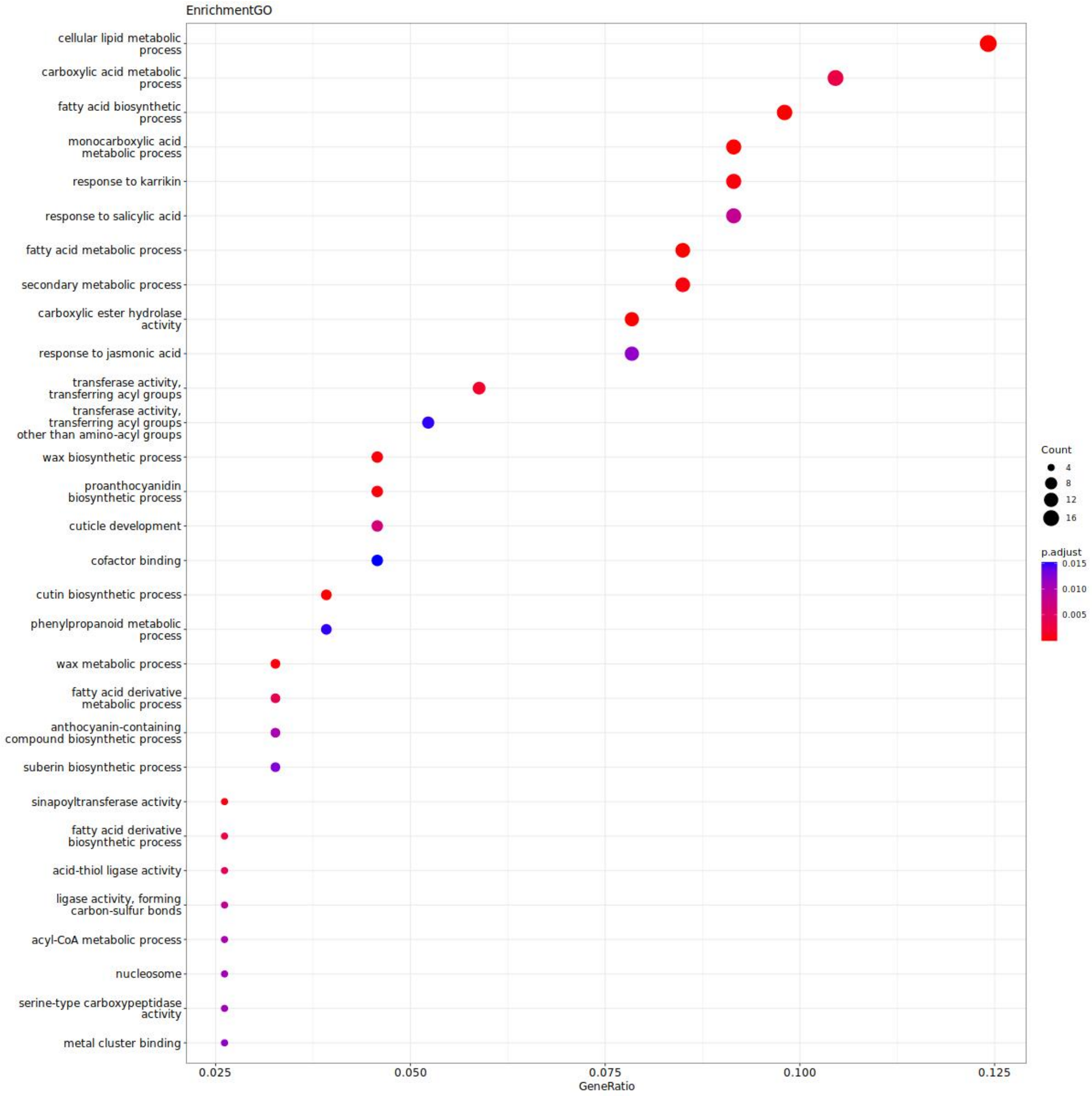

Figure S7-11 The GO enrichment dotpot of Guard cell of *R. sericea*

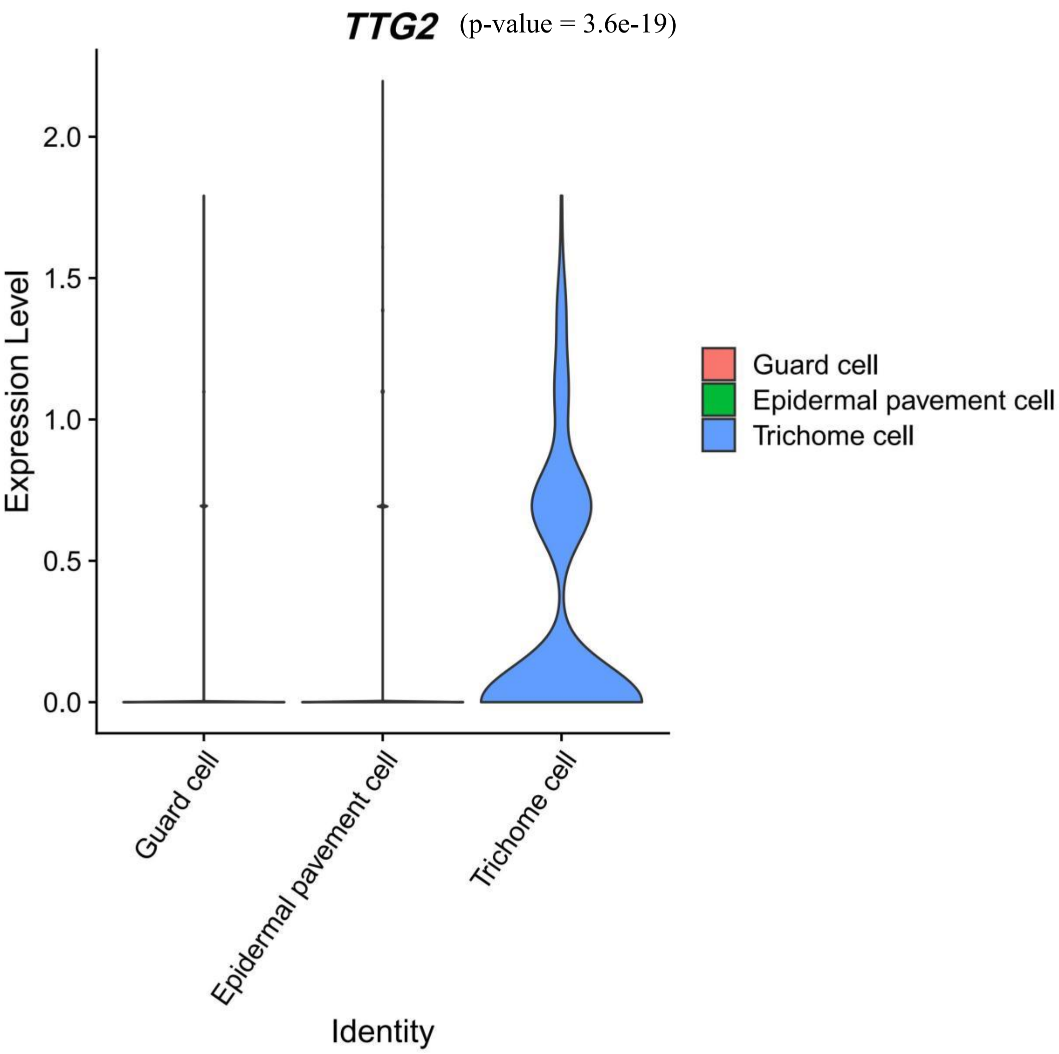

Supplementary Figure 8 The violin plot of *TTG2* gene expression in *R. sericea* leaf epidermal cell subtypes.

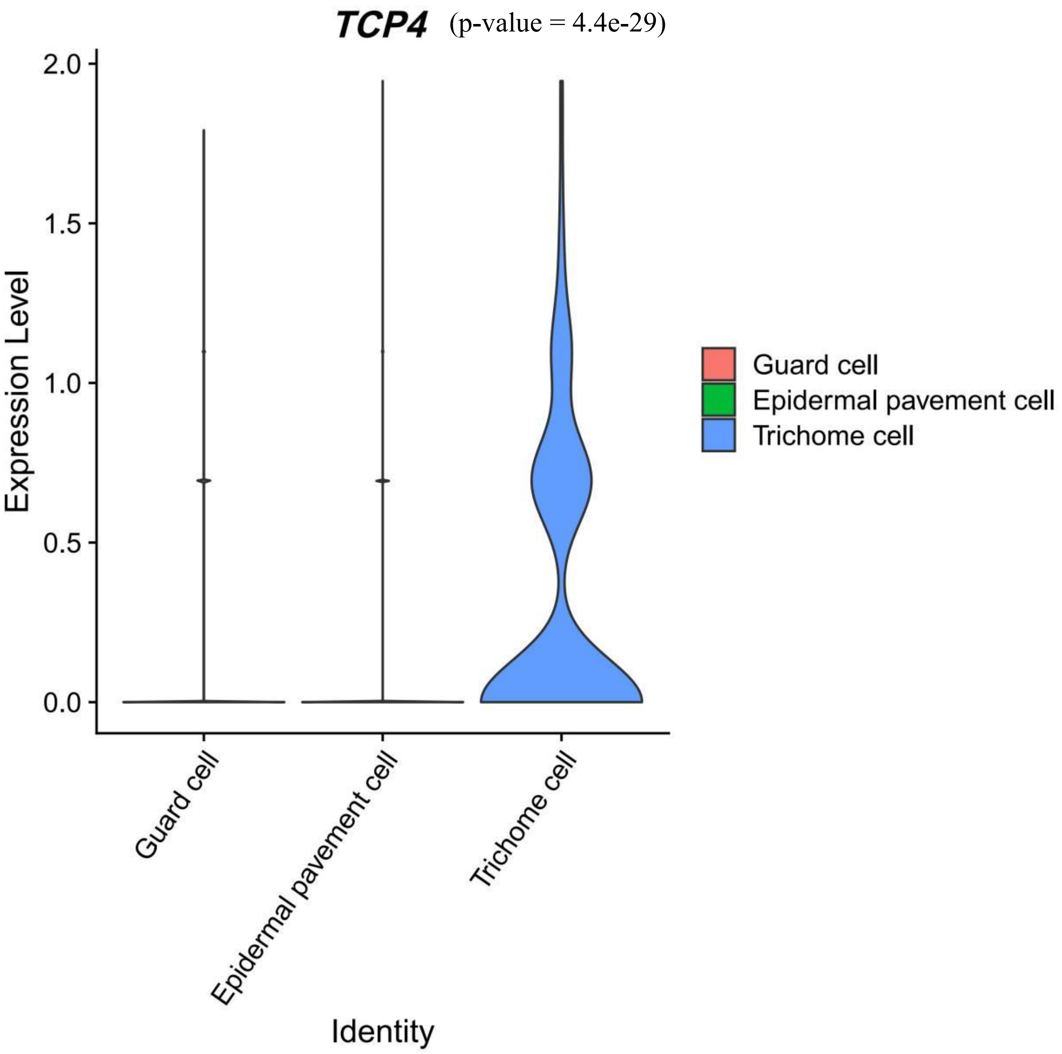

Supplementary Figure 9 The violin plot of *TCP4* gene expression in *R. sericea* leaf epidermal cell subtypes.

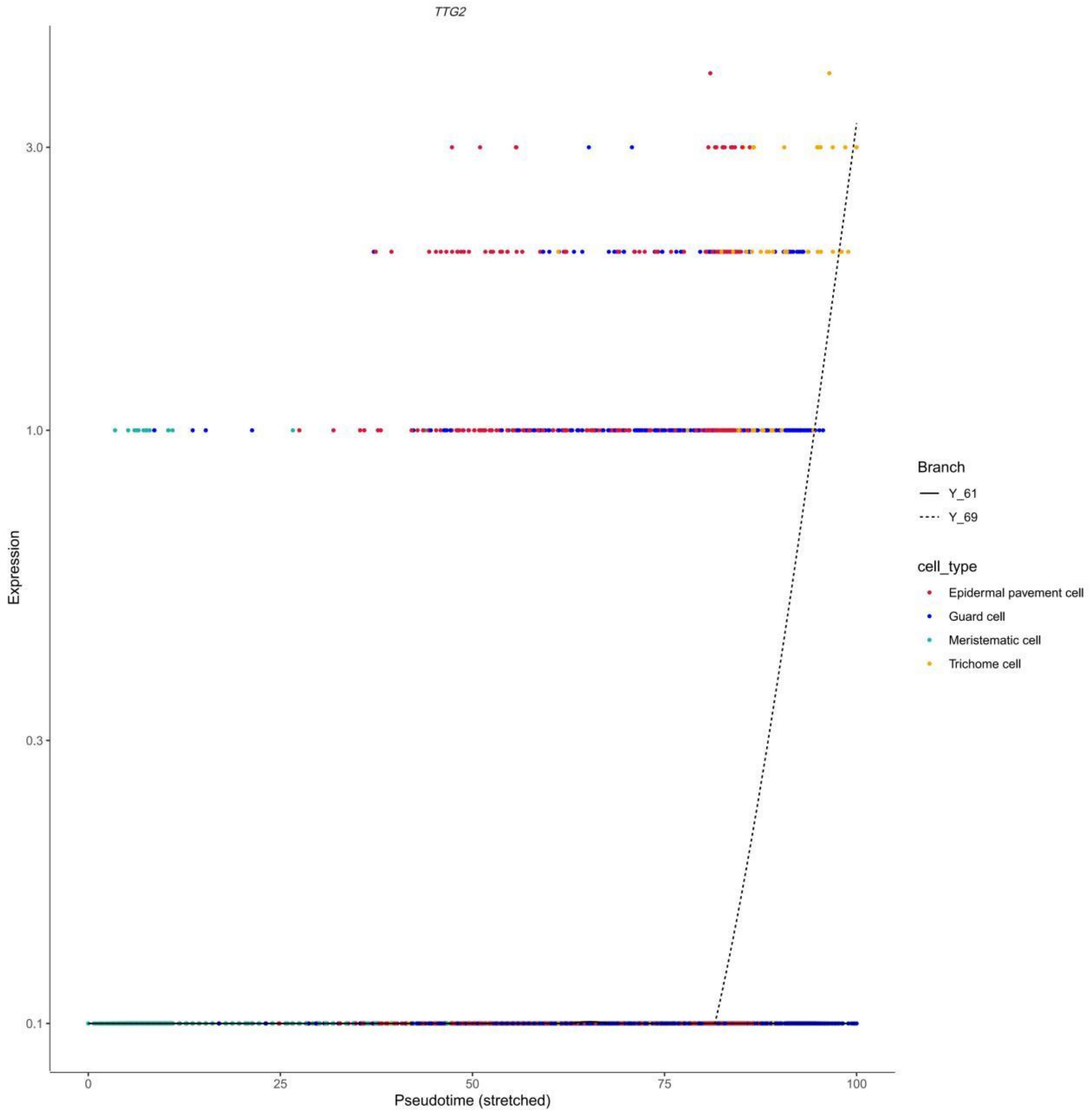

Supplementary Figure 10 Expression trends of *TTG2* in relation to trichome cell fate determination. The dotted line indicates the expression changes of *TTG2* along the pseudo-temporal differentiation trajectory of trichomes.

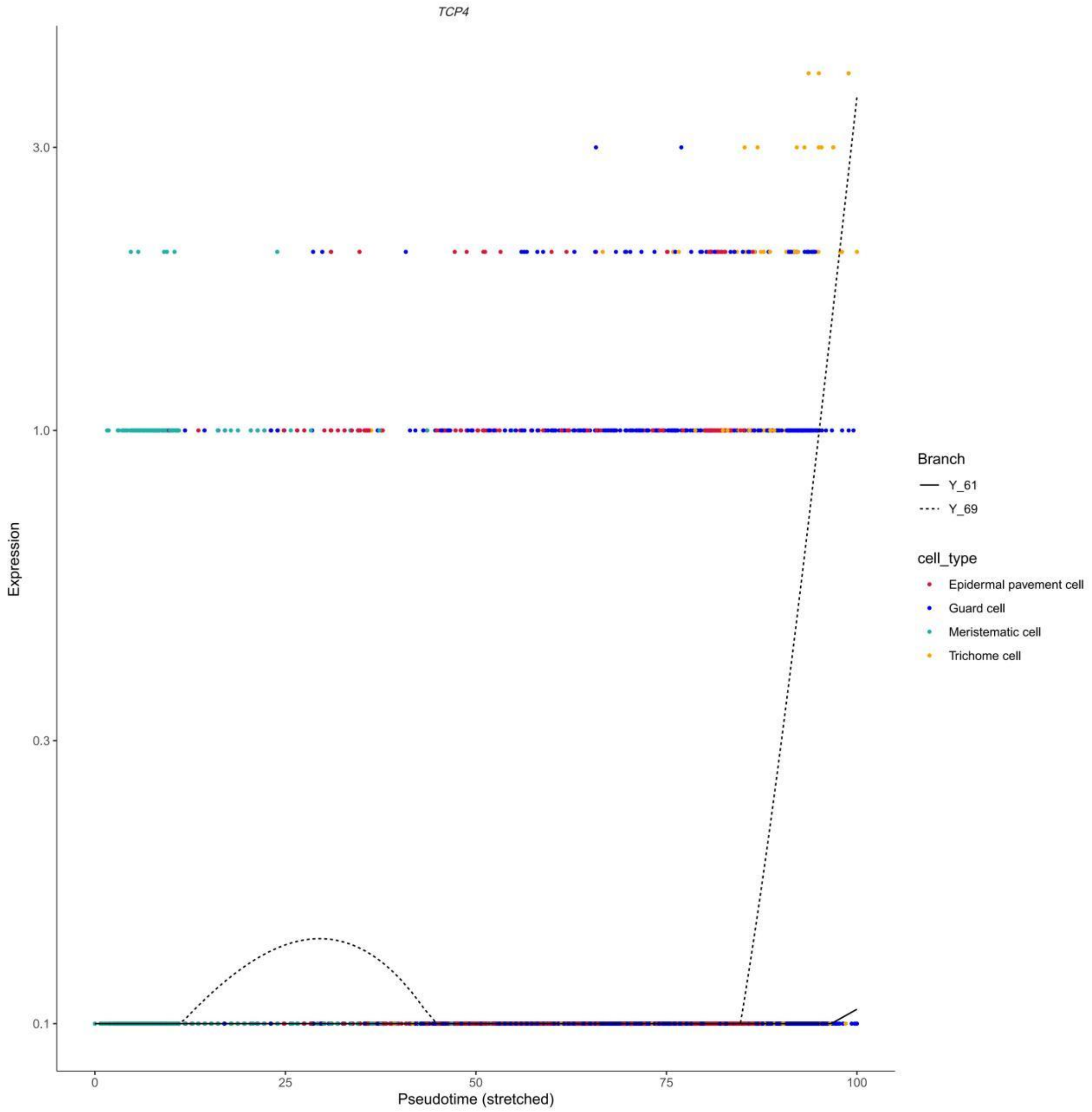

Supplementary Figure 11 Expression trends of *TCP4* in relation to trichome cell fate determination. The dotted line indicates the expression changes of *TCP4* along the pseudo-temporal differentiation trajectory of trichomes.
